# The clinical outcomes of imaging modalities for surgical management Cushing’s disease – A systematic review and meta-analysis

**DOI:** 10.3389/fendo.2022.1090144

**Published:** 2023-01-13

**Authors:** Chan Hee Koh, Danyal Z. Khan, Ronneil Digpal, Hugo Layard Horsfall, Ahmad M. S. Ali, Stephanie E. Baldeweg, Pierre-Marc Bouloux, Neil L. Dorward, William M. Drake, Jane Evanson, Joan Grieve, Danail Stoyanov, Márta Korbonits, Hani J. Marcus

**Affiliations:** ^1^ Victor Horsley Department of Neurosurgery, National Hospital for Neurology and Neurosurgery, London, United Kingdom; ^2^ Wellcome/EPSRC Centre for Interventional and Surgical Sciences, University College London, London, United Kingdom; ^3^ Department of Neurosurgery, Royal Stoke University Hospital, Stoke, United Kingdom; ^4^ Department of Neurosurgery, University Hospital Southampton, Southampton, United Kingdom; ^5^ Department of Neurosurgery, The Walton Centre, Liverpool, United Kingdom; ^6^ Department of Diabetes and Endocrinology, University College Hospital, London, United Kingdom; ^7^ Centre for Obesity & Metabolism, Department of Experimental & Translational Medicine, Division of Medicine, University College London, London, United Kingdom; ^8^ Centre for Neuroendocrinology University College London Medical School, London, United Kingdom; ^9^ Centre for Endocrinology, Barts and The London School of Medicine, Queen Mary University of London, London, United Kingdom; ^10^ Department of Radiology, Barts Health NHS Trust, London, United Kingdom

**Keywords:** cushing, imaging, pituitary, Cushing’s disease, MRI, PET, transsphenoidal, advanced imaging

## Abstract

**Introduction:**

Cushing’s disease presents major diagnostic and management challenges. Although numerous preoperative and intraoperative imaging modalities have been deployed, it is unclear whether these investigations have improved surgical outcomes. Our objective was to investigate whether advances in imaging improved outcomes for Cushing’s disease.

**Methods:**

Searches of PubMed and EMBASE were conducted. Studies reporting on imaging modalities and clinical outcomes after surgical management of Cushing’s disease were included. Multilevel multivariable meta-regressions identified predictors of outcomes, adjusting for confounders and heterogeneity prior to investigating the effects of imaging.

**Results:**

166 non-controlled single-arm studies were included, comprising 13181 patients over 44 years.

The overall remission rate was 77.0% [CI: 74.9%-79.0%]. Cavernous sinus invasion (OR: 0.21 [CI: 0.07-0.66]; p=0.010), radiologically undetectable lesions (OR: 0.50 [CI: 0.37–0.69]; p<0.0001), previous surgery (OR=0.48 [CI: 0.28–0.81]; p=0.008), and lesions ≥10mm (OR: 0.63 [CI: 0.35–1.14]; p=0.12) were associated with lower remission. Less stringent thresholds for remission was associated with higher reported remission (OR: 1.37 [CI: 1.1–1.72]; p=0.007). After adjusting for this heterogeneity, no imaging modality showed significant differences in remission compared to standard preoperative MRI.

The overall recurrence rate was 14.5% [CI: 12.1%-17.1%]. Lesion ≥10mm was associated with greater recurrence (OR: 1.83 [CI: 1.13–2.96]; p=0.015), as was greater duration of follow-up (OR: 1.53 (CI: 1.17–2.01); p=0.002). No imaging modality was associated with significant differences in recurrence.

Despite significant improvements in detection rates over four decades, there were no significant changes in the reported remission or recurrence rates.

**Conclusion:**

A lack of controlled comparative studies makes it difficult to draw definitive conclusions. Within this limitation, the results suggest that despite improvements in radiological detection rates of Cushing’s disease over the last four decades, there were no changes in clinical outcomes. Advances in imaging alone may be insufficient to improve surgical outcomes.

**Systematic Review Registration:**

https://www.crd.york.ac.uk/PROSPERO/, identifier CRD42020187751.

## Introduction

Cushing’s disease is a condition of hypercortisolaemia secondary to an adrenocorticotropic hormone (ACTH) releasing pituitary adenoma. Untreated hypercortisolaemia produces a spectrum of symptomatology which causes major morbidity with significant impact on quality of life and life expectancy (1, 2).

The diagnosis of Cushing’s disease can be difficult, requiring a carefully considered multidisciplinary approach involving endocrinology, diagnostic and interventional radiology, and neurosurgery. Although Cushing’s disease is the most likely cause of increased ACTH and of endogenous hypercortisolaemia, ectopic sources of ACTH are possible (2, 3).

Preoperative magnetic resonance imaging (MRI) is a well-established tool in the diagnostic workup for Cushing’s disease. However, ACTH adenomas are most often small at the time of presentation, with an estimated 40-60% undetectable on MRI (1, 2, 4). Inferior petrosal sinus sampling (IPSS) is an invasive investigation that can confirm a pituitary source of ACTH excess. While IPSS may also help lateralise adenomas in selected cases, the frequently central location of lesions and venous asymmetry renders this unreliable (5). Other preoperative and intraoperative imaging modalities have been deployed in an attempt to improve detection rates (4, 6, 7). However, impact of these on clinical outcomes is unclear.

We conducted a systematic review and meta-analysis to investigate whether advances in imaging has improved outcomes in Cushing’s disease.

## Methods

### Search strategy and selection criteria

The protocol was registered with PROSPERO (CRD42020187751) and uploaded *a priori* on medRxiv (8). This study is reported in accordance with MOOSE guidelines and PRISMA statement (9, 10).

Searches of PubMed and EMBASE were last updated on 1 June 2022, with no limits on the date of publication. The search strategy was created using a PICO approach (**Supplementary Table 1**), and further refined *post hoc* to increase sensitivity for advanced imaging methods.

Studies reporting both the type of imaging used and surgical outcomes in patients with Cushing’s disease were included. Conference abstracts and non-English articles were excluded due to resource constraints. Studies utilising only preoperative MRI were excluded if they had fewer than 10 patients both to maximise numerical stability and due to resource constraints. Conversely, studies utilising other imaging methods were excluded only if the study population was less than 3 to maximise sensitivity for non-standard imaging methods.

### Data extraction

Geographical (region, country, centre), demographic (age, proportion of males, follow-up, previous treatments), biochemical data (preoperative cortisol, ACTH, dexamethasone suppression test, urinary free cortisol, salivary cortisol) and characteristics of the pituitary lesions on preoperative imaging (≥10mm, <10mm, undetectable, cavernous sinus invasion) were extracted. The definitions and thresholds of remission were recorded. If no demographic data for the specific subgroups were available, then the values for the overall study population were taken for the subgroup. If there was potential overlap in datasets between included studies, only the largest of these was included.

Details of preoperative MRI, such as Tesla-strength, dynamic contrast (DMRI), or gradient echo sequences (GRE) were recorded. The relative proportions of other types of imaging were extracted, along with outcomes (remission and recurrence). For the purposes of this study, imaging modality refers to the technology used to obtain the images (e.g. ultrasound, MRI), whereas MRI sequences are used to refer to the specific sequence or protocol (e.g. DMRI, GRE, 3-Tesla).

For most variables, we extracted averages or proportions. The exception was for MRI sequences, where there was rarely sufficient detail to extract proportions. These were therefore simply coded TRUE/FALSE.

The primary outcome was remission after single surgery. Secondary outcome was recurrence rate.

### Quality assessment

Study quality was assessed using the National Institutes of Health case series quality assessment tool (11). This was a deviation from the protocol, as the ROBINS-1 is not designed to assess uncontrolled studies, which formed the majority in our review.

### Meta-analysis

Statistical analyses were conducted using *R* statistical programming (12) and *metafor* (13), with parallelisation using *doParallel* (14). Data were visualised using *ggplot2* (15). A calculator tool was created (http://tiny.cc/QS-CushingSR) to explore the complex interactions within the multivariable models, and to give an estimate of remission or recurrence (16).

Remission and recurrence rates were logit-transformed for analysis, and back-transformed to either rate or odd ratios (OR) for reporting. If the number of events was zero, 0.5 and 1 were added to the numerator and denominator respectively for numerical stability.

The study and its subgroups were taken as nested random effects in multilevel meta-analysis, to account for within-study correlation between subgroups. Both univariable and multivariable analyses were conducted, with the latter reported as the primary analysis.

The variables under investigation in multivariable modelling were baseline variables (radiological lesion ≥10mm, undetectable lesion, cavernous sinus invasion, previous surgery, cortisol remission threshold, age, sex), imaging modalities, MRI sequences (GRE, DMRI, 3-Tesla), year of publication, and length of follow-up for recurrence only.

### Adjustments for heterogeneity

The baseline variables were scrutinised and the heterogeneity subsequently adjusted for using multivariable modelling to investigate for the effect of imaging modalities on outcomes. For missing values in multivariable analyses were multiple imputed using *MICE v3.11.0* (17).

The Bayesian Information Criterion (BIC) was used for model selection, which favours model sparsity compared to Akaike Information Criterion (18). The effects of MRI sequences were investigated separately to imaging modalities due to differences in coding (TRUE/FALSE for the former, proportions for the latter). In assessing the impact of each variable, we considered their effects on overall model fit as measured by BIC, and their magnitudes and statistical significance (threshold α<0.05).

### Publication bias

For univariable analysis, we conducted visual inspection of funnel plots, Duval’s trim-and-fill and Egger’s regression to investigate potential publication bias.

## Results

### Study selection and characteristics

**Figure 1:**
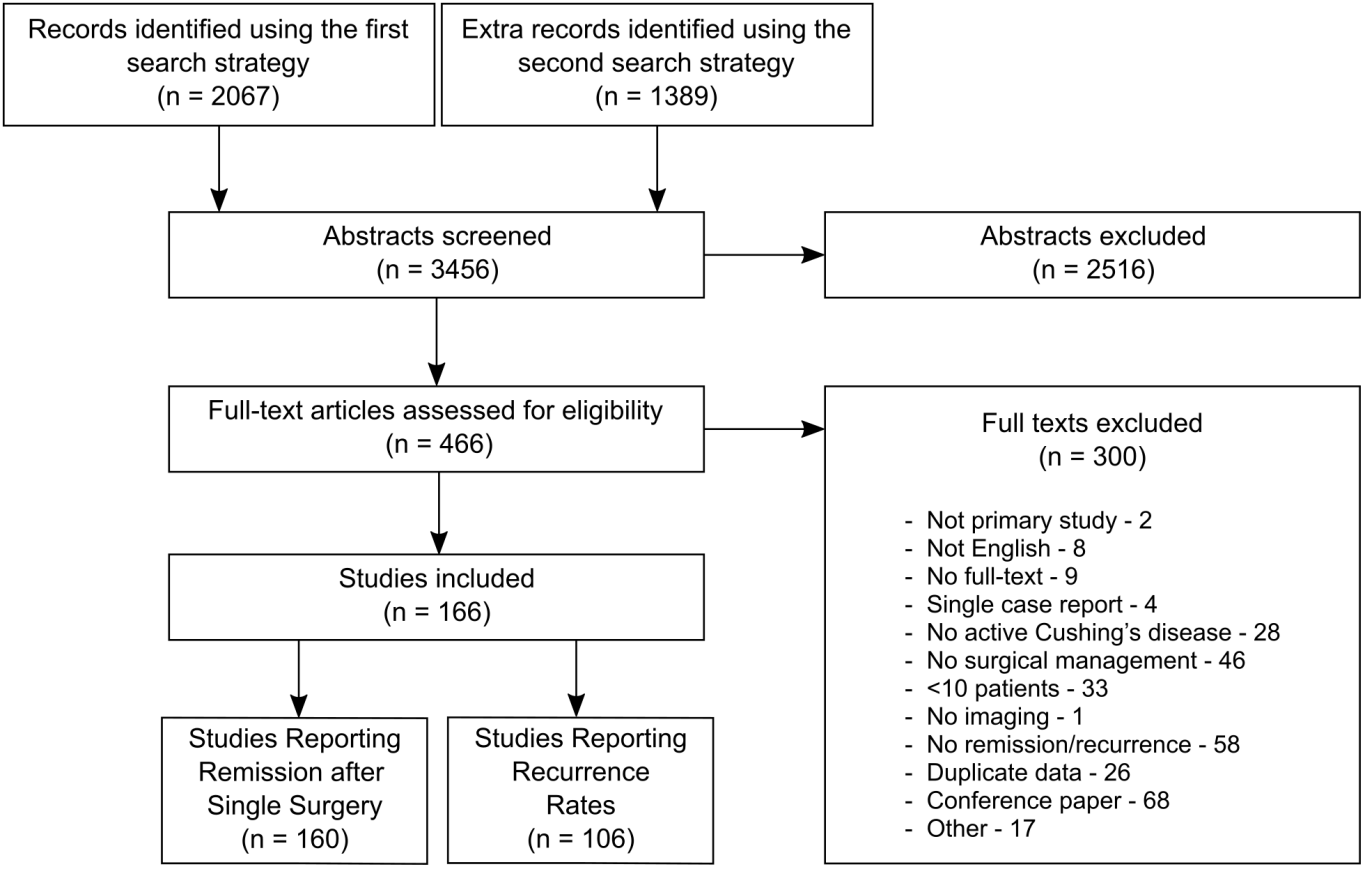
Study flow diagram.

The search returned 2962 records; 166 studies with 13181 patients were eligible for inclusion (**Figure 1**; **Supplementary Tables 2**-**4**). All included studies were of single-arm uncontrolled cohort designs as pertaining to imaging, and the overall quality of design and reporting within the literature was poor (quality assessment presented in **Supplementary Table 5**). Therefore, meta-analysis was conducted by pooling effect sizes from single-arm cohorts. Our search returned only one paper that directly compared remission rates between preoperative MRI only and another imaging modality (intraoperative ultrasound) (19). Statistical testing was not reported, although our own Fisher’s exact testing on the unpaired data showed no significant difference. This study was excluded from our meta-analysis as the number of operations undertaken to achieve remission was unclear.

### Overall estimated remission rates

**Figure 2:**
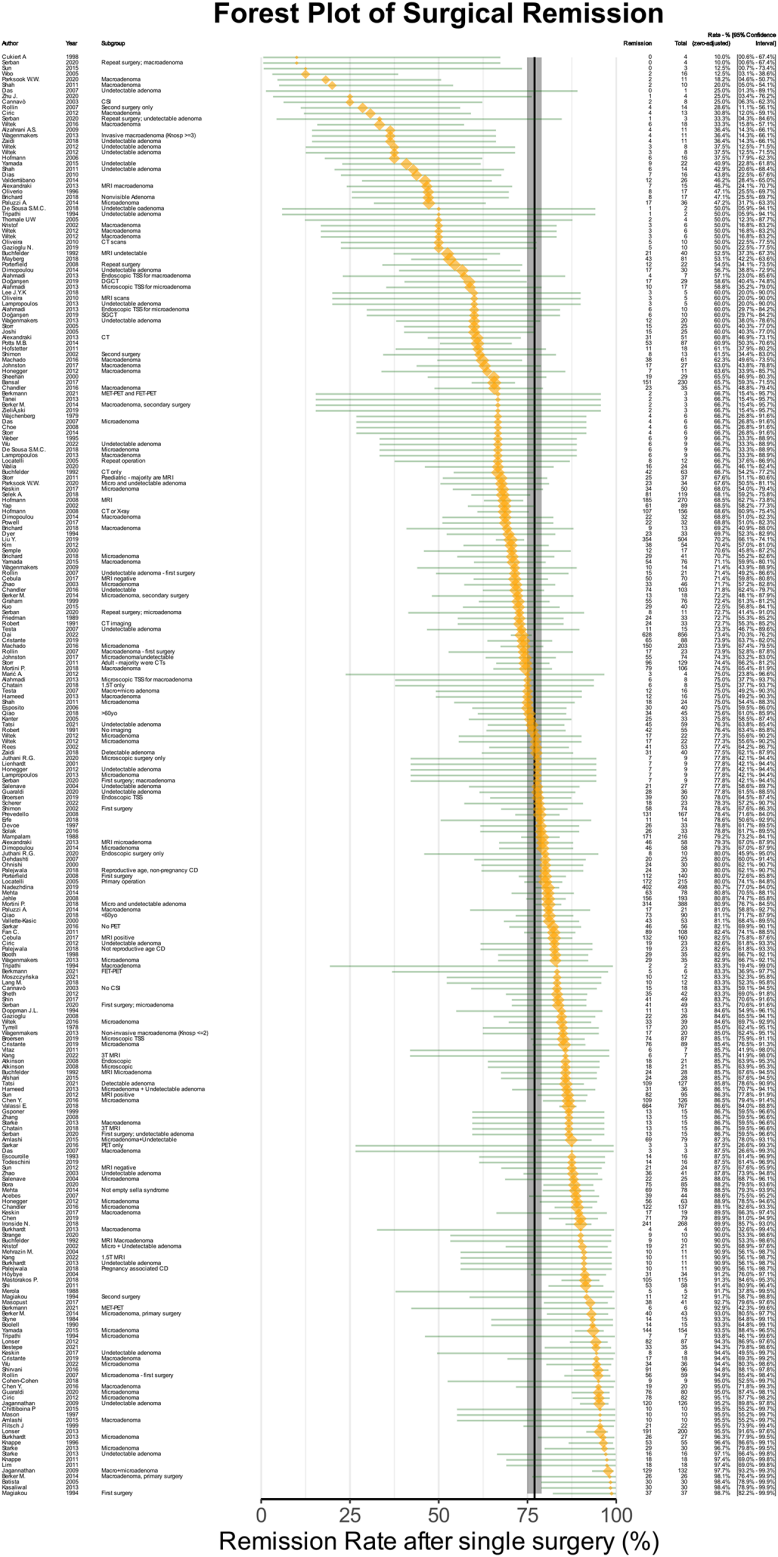
Forest plot of remission after single surgery. The orange points denote sample level effect, with the size proportional to the weight. The green horizontal lines denote the 95% confidence interval for the sample. The solid vertical black line denotes the summary estimate for the univariable meta-analysis, and the vertical shaded area denotes the 95% confidence interval of the summary estimate.

160 studies, comprising 12944 patients over 44 years, reported remission rates after single surgery. The pooled estimate of the remission rate by univariable analysis was 77.0% [CI: 74.9%-78.98%] (**Figure 2**). The funnel plot was symmetrical on visual inspection (**Supplementary Figure 1**), and Egger’s regression test was not significant (p=0.09). The non-parametric Duval’s trim-and-fill method showed an estimated 29 (of total 286) missing cohorts, which did not substantially affect the effect size (estimated bias of 2.8%).

### Baseline predictors of remission

**Figure 3:**
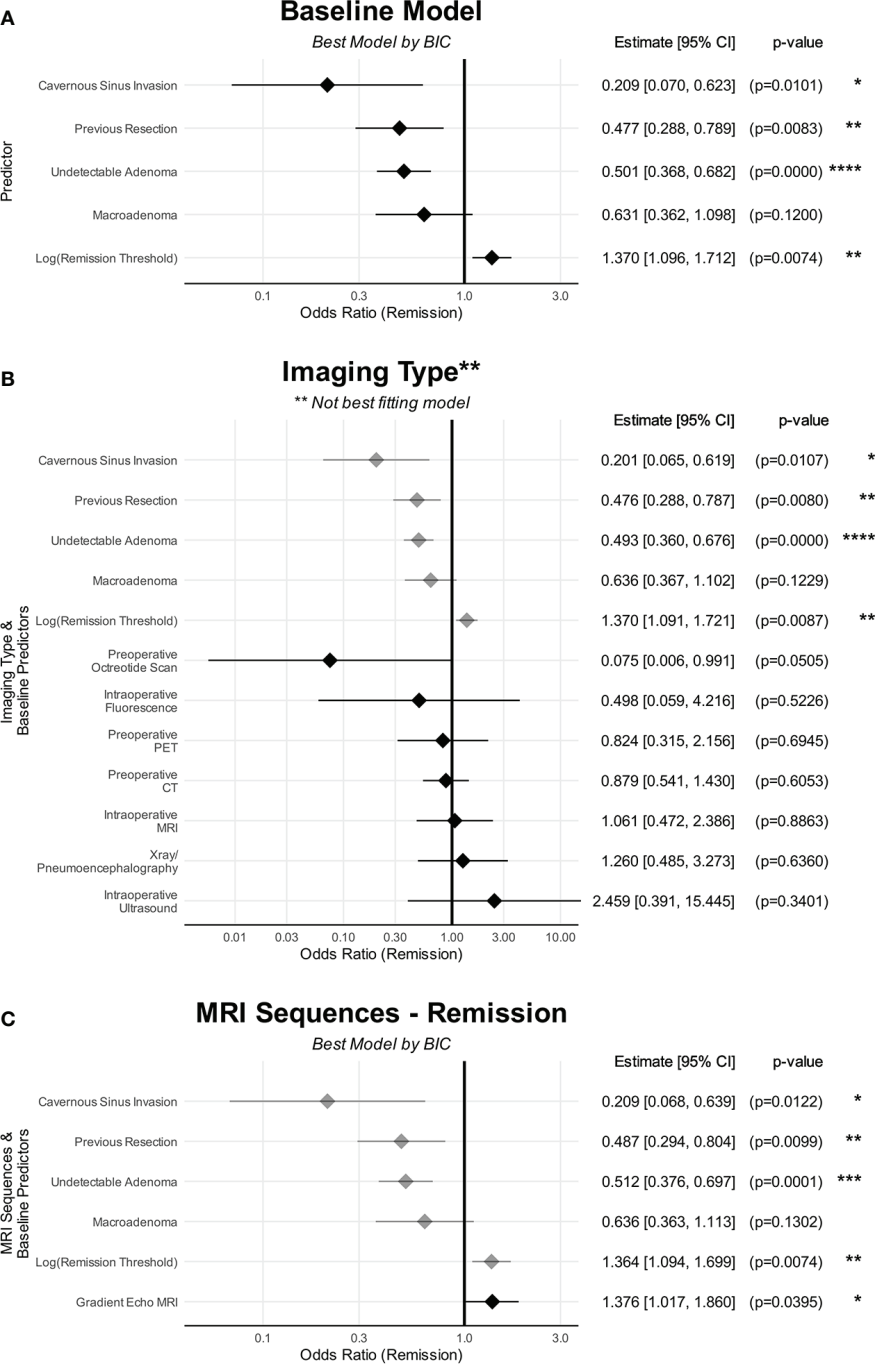
The effects of predictors on remission. **(A)** Baseline characteristics. **(B)** Imaging types. **(C)** MRI sequences. **(A)** shows the baseline characteristics that were predictive of remission. **(B)** No imaging modality was predictive of remission compared to preoperative MRI. **(C)** Only GRE was found to be predictive of remission compared to standard MRI sequences/protocols whereas DMRI and Tesla strength were not. The point denotes the estimated effect size, and the line denotes the 95% confidence interval of that estimate. Baseline variables are in grey The point denotes the estimated effect size, and the line denotes the 95% confidence interval of that estimate. CI, confidence interval. *p < 0.05, **p < 0.01, ***p < 0.001, ****p < 0.0001.

Multivariable meta-regression analysis was conducted using 26111 permutations of baseline variables, imaging modalities, MRI sequences and year of publication (**Supplementary Table 6**). The best-fitting baseline model indicated the variables that best predicted lower remission rates were: cavernous sinus invasion [OR(Remission)=0.209 (CI: 0.066-0.662), p=0.010], previous surgery [OR(Remission)=0.477 (CI: 0.280–0.811), p=0.008], radiologically undetectable lesion [OR(Remission)=0.501 (CI: 0.366–0.686), p<0.0001], and lesion ≥10mm [OR(Remission)=0.63 (CI: 0.35–1.14), p=0.12]. Cortisol threshold were a significant confounding variable, with less stringent cortisol remission thresholds being predictive of higher remission rates [OR(Remission)=1.37 (CI: 1.10–1.72), p=0.007] (**Figure 3A**; **Supplementary Table 7**). The effects of this multivariable model can be viewed using an online calculator created for this purpose (http://tiny.cc/QS-CushingSR) (16). Within this best baseline model, there was still significant heterogeneity (p<0.0001). This baseline model was then used for subsequent analyses discussed below (shown in **Figures 3B, C**).

**Figure 4:**
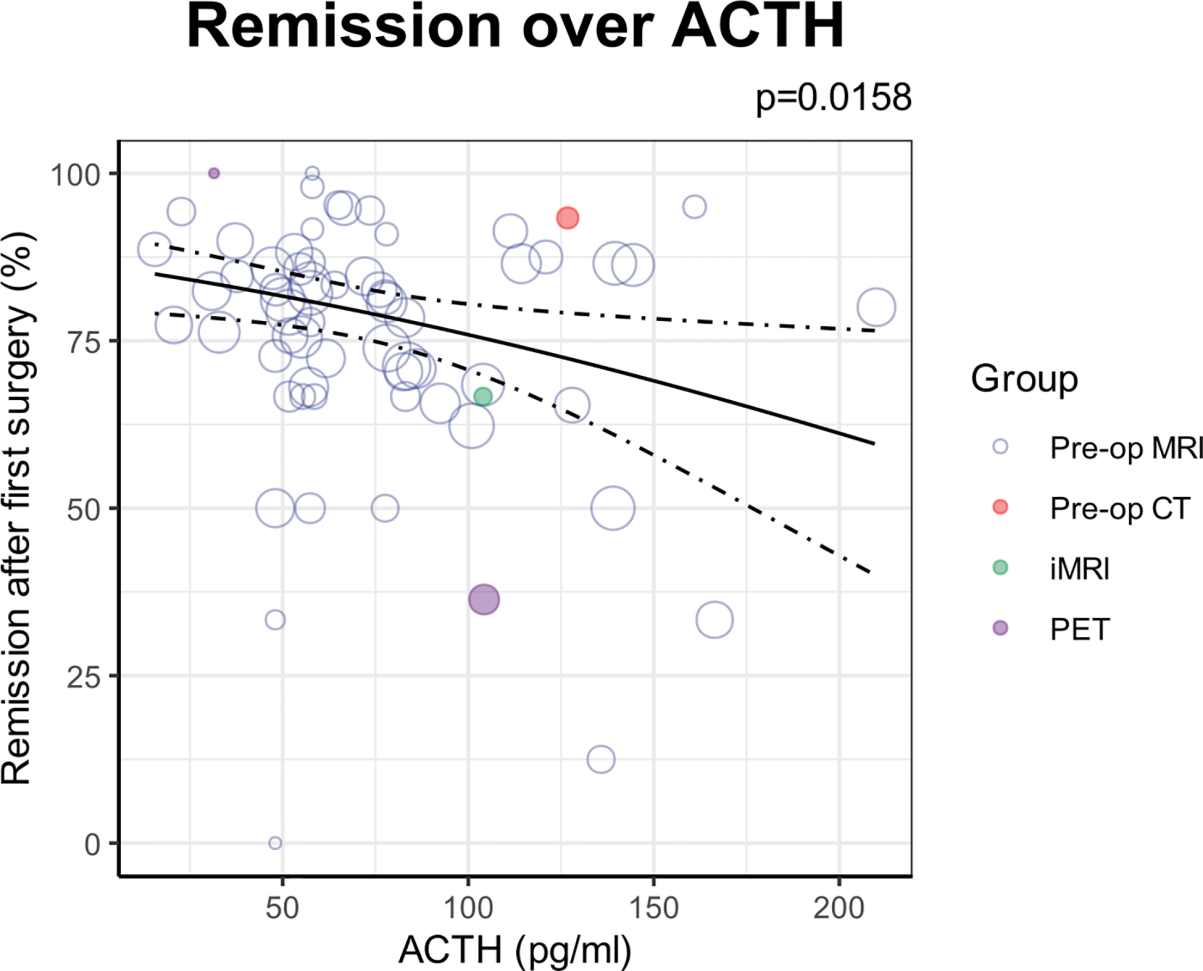
ACTH as an additional predictor of remission found on univariable analysis. Each circle denotes the sample level effect, with the size proportional to the weight. The solid line denotes the regression line, and the dotted lines denote the 95% confidence interval of the line.

Univariable analysis found preoperative ACTH level to also be significant negative predictors with remission rates (**Figure 4**). However, this was not included in the multivariable regression as this was not reported for 78.2% (237/303) of cohorts.

### Imaging modalities and remission

The studies reporting remission utilised the following preoperative non-standard imaging modalities (i.e. anything other than preoperative MRI):

• CT in 794 patients in 31 studies, and unknown numbers in 2 studies.
• Positron Emission Tomography (PET) in 63 patients in 5 studies (18F-FDG tracers in 2 studies, 68Ga-CRH in 1, 1 study using 11C-methionine and 18-F-Fluoroethyl-L-tyrosine separately, and unspecified in 1), and unknown numbers in 3 studies (tracer unspecified in all)
• Octreotide scan (111In-pentetreotide tracer) in 4 patients in 1 study.
• Either X-rays or pneumoencephalography in 358 patients in 8 studies

Intraoperative imaging modalities included:

• Intraoperative MRI in 160 patients in 8 studies.
• Intraoperative ultrasound in 31 patients in 2 studies.
• Intraoperative fluorescence in 5 patients in 1 study.

Of the non-standard imaging modalities, only inclusion of octreotide scan into the model marginally improved model fit [BIC 675.5 vs 676.4] (**Supplementary Table 8**). However, the number of studies and patients reporting octreotide scanning was small (4/4 in one study), and the variable did not meet the threshold for significance [OR(Remission)=0.075 (CI: 0.006 – 1.005), p=0.05] (**Supplementary Table 9**). No other imaging modality improved model fit or had significant associations with remission rates compared to preoperative MRI only (**Figure 3B**).

Removing either or both of lesion ≥10mm and undetectable lesion variables from the model did not change the results, reducing the likelihood that effects were attenuated by collinearity between these and imaging types (**Supplementary Tables 10**-**12**).

### MRI sequences and remission

Of the studies reporting remission, 36 reported use of GRE, 61 reported DMRI and 31 reported 3-Tesla MRI.

Incorporating the reported use of GRE was found to improve prediction of remission over baseline variables only [BIC 675.9 vs 676.4] (**Supplementary Table 13**), and was a significant predictor of increased remission rates compared to standard preoperative MRI [OR(Remission)=1.38 (CI: 1.02–1.86), p=0.040] (**Figure 3C**).

DMRI and 3-Tesla worsened model fit and were not significant predictors of remission.

Again, excluding the detectability and macroadenoma variables did not substantially change the results, although GRE then became a stronger predictor of remission [OR(Remission)=1.44 (CI: 1.07-1.95); p=0.018], likely due to the collinearity between GRE and lesion detectability (**Supplementary Figure 2**; **Supplementary Tables 14**-**16**).

### Overall estimated recurrence rates

**Figure 5:**
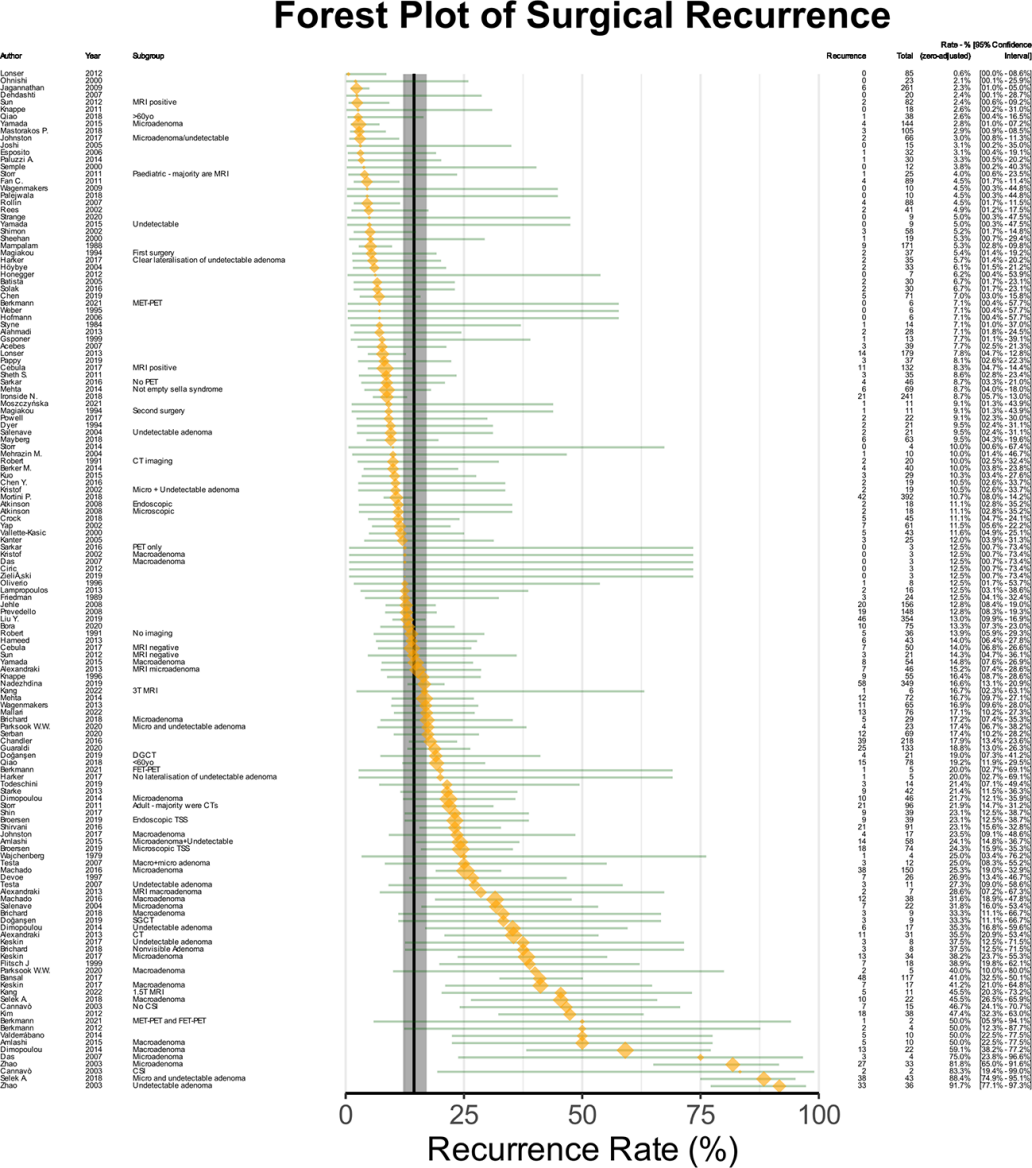
Forest plot of recurrence rates. The orange points denote sample level effect, with the size proportional to the weight. The green horizontal lines denote the 95% confidence interval for the sample. The solid vertical black line denotes the summary estimate for the univariable meta-analysis, and the vertical shaded area denotes the 95% confidence interval of the summary estimate.

106 studies, consisting of 7185 patients over 43 years, reported recurrence rates. The pooled estimate of the recurrence rate by univariable analysis was 14.5% [CI: 12.1%-17.1%] (**Figure 5**). The funnel plot was asymmetrical on visual inspection, and was significant on Egger’s regression test (p=0.004; **Supplementary Figure 3**). Duval’s trim-and-fill showed an estimated 33 (of total 175) missing cohorts causing estimated bias of 4.9%.

### Baseline predictors of recurrence

**Figure 6:**
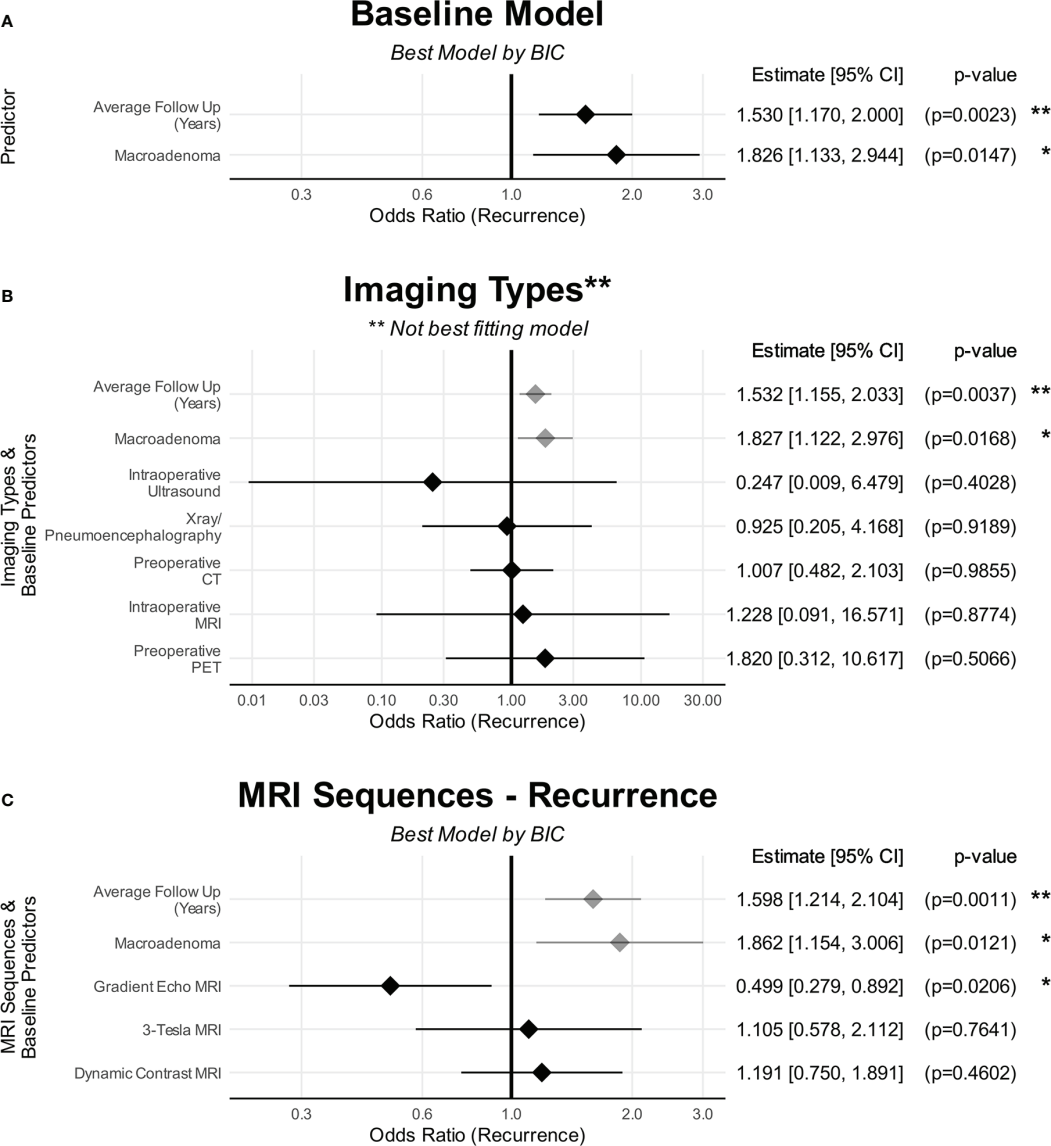
The effects of predictors on recurrence. **(A)** Baseline characteristics. **(B)** Imaging types. **(C)** MRI sequences. **(A)** shows the baseline characteristics that were predictive of recurrence. **(B)** No imaging modality was predictive of recurrence compared to preoperative MRI. **(C)** Only GRE was found to be predictive of recurrence compared to standard MRI sequences/protocols whereas DMRI and Tesla strength were not. The point denotes the estimated effect size, and the line denotes the 95% confidence interval of that estimate. Baseline variables are in grey The point denotes the estimated effect size, and the line denotes the 95% confidence interval of that estimate. CI, confidence interval. *p < 0.05, **p < 0.01, ***p < 0.001, ****p < 0.0001.

A multivariable analysis was conducted, with 52223 models created using all possible permutations of included variables (**Supplementary Table 17**). The best-fitted baseline model showed that lesion ≥10mm was predictive of higher reported recurrence rates [OR(Recurrence)=1.83 (CI: 1.13–2.96), p=0.015], with duration of follow-up being a significant confounding variable [OR(Recurrence)=1.53 per year (CI: 1.17–2.01), p=0.002] (**Figure 6A**; **Supplementary Table 18**). The effects of this multivariable model can be viewed using an online calculator created for this purpose (http://tiny.cc/QS-CushingSR) (16). The best-fitted baseline model for recurrence still showed significant heterogeneity (p<0.0001).

### Imaging modalities and recurrence

The following preoperative imaging modalities were utilised in studies reporting recurrence:

• CT in 1380 patients in 23 studies, and unknown numbers in 2 studies.
• X-rays or pneumoencephalography in 256 patients in 7 studies.
• PET in 16 patients in 2 studies (1 study using 11C-methionine and 18-F-Fluoroethyl-L-tyrosine separately; unspecified tracer in the other), and unknown numbers in 1 study (tracer unspecified).

Of the intraoperative imaging modalities:

• intraoperative MRI in 51patients in 3 studies.
• intraoperative ultrasound in 18 patients in 1 study.

No imaging modality resulted in an improvement in model fit for recurrence (**Figure 6B**; **Supplementary Table 19**). The same applied when lesion size was excluded from the model fit (**Supplementary Tables 20**-**22**).

### Advanced MRI sequences and recurrence

Of the studies reporting recurrence rates, 23 reported use of GRE, 38 reported DMRI and 18 utilised 3-Tesla MRI.

Incorporating GRE resulted in the best model fit for recurrence rates [BIC 431.4 vs 433.8] (**Supplementary Tables 23**-**26**), with studies reporting the use of GRE reporting significantly reduced recurrence rates compared to standard preoperative MRI [OR(Recurrence)=0.540 (CI: 0.315–0.924); p=0.025] (**Figure 6C**). Within this best performing model, there was still significant heterogeneity (p<0.0001). Tesla strength and DMRI did not result in better model fit.

### Detection rates and outcomes over time

**Figure 7:**
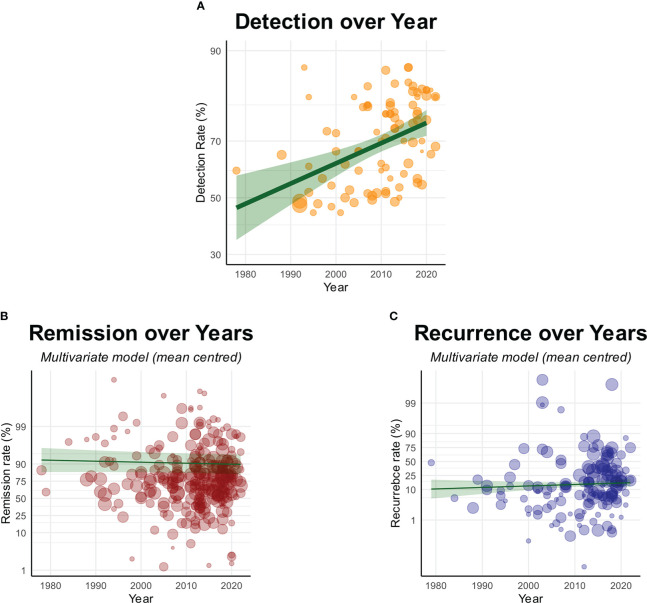
Graphs showing **(A)** Significant improvement in detection rates over time, but with **(B)** no improvements in remission or **(C)** recurrence over time. Each circle denotes the sample level effect, with the size proportional to the weight. The solid line denotes the regression line, and the grey area denotes the 95% confidence interval of the line.

There were significant improvements in detection rates of suspected adenomas in patients with Cushing’s disease over four decades [OR(Detection)=1.03 per year (CI: 1.02–1.05), p<0.0001] (**Figure 7A**). Although no single imaging modality was significantly associated with improved detection, there were non-significant trends toward improved detection with advanced imaging methods such as GRE and PET, and worse detection with older imaging modalities such as CT (**Supplementary Figure 4**).

Despite improved detection rates, there was no associated improvement in remission rates over 43 years [OR(Remission)=0.99 per year (CI: 0.98–1.01), p=0.41] (**Figure 7B**). Removing the sizes and detectability of lesions did not substantially change the result (**Supplementary Tables 28**-**30**).

Likewise, there were no changes in reported recurrence rates [OR(Recurrence)=1.01 per year (CI: 0.99–1.03), p=0.31] (**Figure 7C**; **Supplementary Table 31**). This was again true when sizes of radiological lesions were removed (**Supplementary Tables 32**-**34**).

Given the relative sparsity of studies at earlier time points, we conducted a sensitivity analysis with studies before 1990, 2000 and 2010 excluded from the analysis. We found that there was no substantial change to these results with these sensitivity analyses (**Supplementary Tables 35**-**40**).

## Discussion

This is, to our knowledge, the largest meta-analysis addressing the investigation and surgical management of Cushing’s disease. Here, we established several disease factors that are associated with clinical outcomes of remission and recurrence, with a calculator created to allow exploration of the complex interactions between these variables (http://tiny.cc/QS-CushingSR) (16). We found that there were no changes in the published remission rates and recurrence rates of Cushing’s disease within the literature over the last four decades. This is despite significant demonstrable improvements in radiological detection of suspected adenomas over the same period. The studies published to date suggest a lack of association between advances in imaging modalities and clinical outcomes, although there may be associations between MRI sequences (namely GRE) and clinical outcomes. However, the confidence in these findings are limited by the poor quality of evidence in the field, and the consequent risk of bias.

### Predictors of outcomes in Cushing’s disease

Our results demonstrate that radiological detectability, previous resections, cavernous sinus invasion, lesion ≥10mm, and the threshold of remission were the strongest baseline predictors of remission (**Figure 3A**). In addition, the multivariate approach allowed us to establish the complex interactions between these variables (16), which was not possible in previously published meta-analyses (20, 21). While our univariable analysis found preoperative ACTH level to be negatively associated with remission (**Figure 4B**), we were unable to include this in our multivariable analysis as the vast majority of studies failed to report this value. In context of the existing literature, one recent retrospective single-centre study found the number of operations and cavernous sinus invasion to be significant negative predictors of remission, in addition to disease duration, macroadenoma and preoperative ACTH level (22). Another single-centre study reported a significant association between microadenoma and improved surgical outcomes (23). A stratified subgroup meta-analysis found adenoma size to be a significant predictor of remission, whereas previous surgical resection was not (20). Another univariate subgroup meta-analysis reported previous resection, radiological detectability, adenoma size and cavernous sinus invasions as significantly associated with remission (21).

Our meta-analysis suggests that the strongest predictors of recurrence are radiological lesions ≥10mm and the duration of follow-up (**Figure 6A**). There is less consensus in the existing literature of the predictive factors for recurrence. One single-centre study reported that only the time to recovery of the hypothalamic-adrenal axis to be a significant predictor of recurrence, whereas postoperative cortisol levels, imaging detectability, macroadenoma, gender and age were not (23). The utility of postoperative biochemistry as predictors of recurrence has been particularly contradictory (23–28). Recently, machine learning approaches have found the most important predictors of recurrence to be postoperative ACTH, age, postoperative cortisol, disease duration, postoperative urinary free cortisol (29, 30). The aforementioned stratified meta-analysis found no significant predictors of recurrence (20).

Outside the scope of this review was the analysis of molecular markers as predictors of outcome. Moreno-Moreno et al. (2022) demonstrated how integrating molecular analysis with clinical and radiological information could predict remission of Cushing’s disease (31).

### Advanced and non-standard imaging modalities

There were no significant changes in reported outcomes amongst a small number of studies that utilised any non-standard preoperative or intraoperative imaging modality (**Figures 3B** and **6B**). The interpretation is limited by the low power given small number of studies reporting on non-standard imaging, and low quality of design and reporting within the published literature resulting in high risk of selection and publication biases and demonstrable heterogeneity.

While methionine-PET has generated considerable interest for investigation of pituitary tumours (32), our meta-analysis was notable for finding a relative lack of methionine-PET studies reporting clinical outcomes thereby making assessment impossible. There was only one included study with 6 patient undergoing methionine-PET/MRI, with remission being achieved in all (33). Although our screen returned three further studies reporting on the use of methionine-PET. these were excluded as either no clinical outcomes were reported (34, 35), or they were selected illustrative case series (36).

### Advanced MRI sequences

The evidence around MRI sequences was suggestive of improved outcomes with GRE imaging (**Figures 3C** and **6C**). GRE allows for thinner slices, and therefore higher resolutions, with multiplanar reformat. The reported use of GRE during the diagnostic workup was a significant predictor of reduced recurrence rates, and was a near-significant predictor of improved remission. There was a weak non-significant trend towards improved detection with GRE (**Supplementary Figure 4**), which presents a possible explanation for this observed effect. The possibility of a false positive finding must be considered, given the poor reporting standards of specific MRI sequences/protocols.

There was no evidence for DMRI or 3T MRI, and these were not predictive of changes in outcomes as given BIC. However, the same limitations apply here to advanced MRI sequences as with imaging modalities, namely poor quality of published literature giving rise to high risk of selection and publication biases.

### Detection rates and outcomes over time

Amongst the included studies, there were no changes in reported outcomes over the last four decades in remission or recurrence rates after surgical management of Cushing’s disease (**Figure 7**). This supports the findings in another recent meta-analysis which showed no difference in outcomes when comparing outcomes between 1980-1989 and 2010-2019 (21). Curiously, this is despite our finding of improvement in detection of suspected adenomas over the same time period. With the detectability of lesions being a significant positive predictor of remission, one would expect an associated increase in remission rates. The great heterogeneity amongst included studies (in population, study design and reporting), along with high risk of publication bias are significant confounding factors. With these limitations in mind, one possible explanation is that there has been increase in detection of lesions that are not ACTH-secreting adenomas. An alternative explanation is that there has been an increase in detection of more challenging adenomas. We speculate that in addition to preoperative visualisation, reliable intraoperative identification of residual disease, and surgical techniques to remove these, are also important (37).

### Strengths and limitations

This is, to our knowledge, the largest meta-analysis addressing the investigation and surgical management of Cushing’s disease. We were able to establish various factors that may act as confounding factors (**Figures 3B** and **6B**) using a robust and comprehensive methodology. In addition to establishing valuable clinical information regarding the prognosis of Cushing’s disease, this allowed for the adjustment for these confounding variables in our subsequent meta-analyses. Another strength of this meta-analysis over previous studies is the multivariate approach taken, which allows investigation of the complex interactions between multiple independent variables.

There are several limitations to this study which merit a cautious interpretation of our findings. First is that there is a low quality of published literature in the field, with all included studies being of uncontrolled single-arm designs, giving rise to high-risk of selection bias. Secondly, while we attempted to adjust for heterogeneity as stated above in conducting comparative meta-analysis between single-arm studies, any statistical correction is unlikely to be as robust as a well-conducted comparative study. In addition heterogeneity also makes investigation of publication bias difficult, as funnel plot asymmetry is likely to reflect heterogeneity rather than publication bias alone. This is particularly important as uncontrolled studies are highly susceptible to publication bias.

## Conclusions

We have established cavernous sinus invasion, radiologically undetectable lesions, lesion ≥10mm, and a history previous resections as the strongest predictors of worse remission, with remission threshold being a significant confounding variable in reported remission. Radiological macroadenoma was the strongest predictor of greater recurrence, with the duration of follow-up being a significant confounding variable.

There is a paucity of high-quality evidence in the published literature reporting on the effects of imaging on clinical outcomes. This, along with substantial heterogeneity, makes it difficult to draw firm conclusions from our results. There is a need for high-quality, prospective, comparative studies with a standardised framework for evaluation and reporting.

Within the limitations outlined above, our results show a lack of improvement in reported outcomes over the last four decades despite significant improvements in detection rates, and a lack of evidence that imaging is associated with changes in clinical outcomes. This may indicate a need for development and evaluation of new approaches beyond merely improving preoperative detection. These may include methods to improve intraoperative identification of tumours and new surgical techniques.

## Data availability statement

The original contributions presented in the study are included in the article/**Supplementary Material**. Further inquiries can be directed to the corresponding author.

## Author contributions

HM and MK were responsible for conception of this study. CK, HM, MK and JE designed the study. Data extraction by CK, DK, RD, HLH, AA. Analysis by CK. Draft manuscript by CK. All authors contributed to the article and approved the submitted version.

## Supplementary material

Supplementary Figure 1 Funnel plot of univariable remission with Duval’s trim-and-fill.

Supplementary Figure 2 Effect of removing macroadenoma from the MRI sequences model. Note that GRE is now a stronger predictor of remission.

Supplementary Figure 3 Funnel plot of univariable recurrence with Duval’s trim-and-fill.

Supplementary Figure 4 Detection rates by imaging modalities and MRI sequences.Detection rates of (A) Imaging modalities and (B) MRI sequences. Models incorporating all imaging modalities and MRI sequences shown regardless of performance (BIC) as none were significantly associated with changes in detection. The point denotes the estimated effect size, and the line denotes the 95% confidence interval of that estimate.

## References

[1] Lonser RR Nieman L Oldfield EH . Cushing’s disease: pathobiology, diagnosis, and management. J Neurosurg (2017) 126(2):404–17. doi: 10.3171/2016.1.JNS152119 27104844

[2] Pivonello R De Martino MC De Leo M Simeoli C Colao A . Cushing’s disease: the burden of illness. Endocrine (2017) 56(1):10–8. doi: 10.1007/s12020-016-0984-8 27189147

[3] Pivonello R De Leo M Cozzolino A Colao A . The treatment of cushing’s disease. Endocr Rev (2015) 36(4):385–486. doi: 10.1210/er.2013-1048 26067718 PMC4523083

[4] Marcus HJ Vercauteren T Ourselin S Dorward NL . Intraoperative ultrasound in patients undergoing transsphenoidal surgery for pituitary adenoma: Systematic review [corrected]. World Neurosurg (2017) 106:680–5. doi: 10.1016/j.wneu.2017.07.054 28736351

[5] Ghorbani M Akbari H Griessenauer CJ Wipplinger C Dastmalchi A Malek M . Lateralization of inferior petrosal sinus sampling in cushing’s disease correlates with cavernous sinus venous drainage patterns, but not tumor lateralization. Heliyon (2020) 6(10):e05299. doi: 10.1016/j.heliyon.2020.e05299 33134585 PMC7586104

[6] Chittiboina P . iMRI during transsphenoidal surgery. Neurosurg Clin N Am (2017) 28(4):499–512. doi: 10.1016/j.nec.2017.05.005 28917279 PMC5661990

[7] Zaidi HA Reyes KDL Barkhoudarian G Litvack ZN Bi WL Rincon-Torroella J . The utility of high-resolution intraoperative MRI in endoscopic transsphenoidal surgery for pituitary macroadenomas: early experience in the advanced multimodality image guided operating suite. Neurosurg Focus (2016) 40(3):E18. doi: 10.3171/2016.1.FOCUS15515 26926058 PMC4992957

[8] Koh CH Khan DZ Horsfall HL Ali A Digpal R Evanson J . Advanced imaging to improve remission rates in patients undergoing transsphenoidal surgery for cushings disease: A protocol for systematic review and meta-analysis. medRxiv (2020). 2020.07.04.20146498. doi: 10.1101/2020.07.04.20146498

[9] Brooke BS Schwartz TA Pawlik TM . MOOSE reporting guidelines for meta-analyses of observational studies (2021). Available at: https://jamanetwork.com/journals/jamasurgery/fullarticle/2778476. 10.1001/jamasurg.2021.0522 33825847

[10] Moher D Shamseer L Clarke M Ghersi D Liberati A Petticrew M . Preferred reporting items for systematic review and meta-analysis protocols (PRISMA-p) 2015 statement. Syst Rev (2015) 4(1):1. doi: 10.1186/2046-4053-4-1 25554246 PMC4320440

[11] National Institues of Health . Study quality assessment tools | NHLBI, NIH. Available at: https://www.nhlbi.nih.gov/health-topics/study-quality-assessment-tools.

[12] R Core Team . R: A language and environment for statistical computing. Vienna, Austria: R Foundation for Statistical Computing (2018). Available at: https://www.R-project.org/.

[13] Viechtbauer W . Metafor: Meta-analysis package for r (2020). Available at: https://CRAN.R-project.org/package=metafor.

[14] Wallig M Corporation M Weston S Tenenbaum D . doParallel: Foreach parallel adaptor for the ‘parallel’ package (2020). Available at: https://CRAN.R-project.org/package=doParallel.

[15] Wickham H . ggplot2: Elegant graphics for data analysis. Verlag New York: Springer (2016). Available at: https://ggplot2.tidyverse.org.

[16] Koh CH . Cushing’s disease and imaging SR calculator. In: Cushing’s disease and imaging SR calculator. Available at: http://tiny.cc/QS-CushingSR. cited 2022 Sep 1.

[17] van Buuren S Groothuis-Oudshoorn K Vink G Schouten R Robitzsch A Rockenschaub P . Mice: Multivariate imputation by chained equations (2020). Available at: https://CRAN.R-project.org/package=mice.

[18] Vrieze SI . Model selection and psychological theory: A discussion of the differences between the akaike information criterion (AIC) and the Bayesian information criterion (BIC). Psychol Methods (2012) 17(2):228–43. doi: 10.1037/a0027127 PMC3366160 22309957

[19] Watson JC Shawker TH Nieman LK DeVroom HL Doppman JL Oldfield EH . Localization of pituitary adenomas by using intraoperative ultrasound in patients with cushing’s disease and no demonstrable pituitary tumor on magnetic resonance imaging. J Neurosurg (1998) 89(6):927–32. doi: 10.3171/jns.1998.89.6.0927 9833817

[20] Dabrh AMA Singh Ospina NM Nofal AA Farah WH Barrionuevo P Sarigianni M . Predictors of biochemical remission and recurrence after surgical and radiation treatments of cushing disease: a systematic review and meta-analysis. Endocr Pract (2016) 22(4):466–75. doi: 10.4158/EP15922.RA 26789343

[21] Stroud A Dhaliwal P Alvarado R Winder MJ Jonker BP Grayson JW . Outcomes of pituitary surgery for cushing’s disease: a systematic review and meta-analysis. Pituitary (2020) 23(5):595–609. doi: 10.1007/s11102-020-01066-8 32691356

[22] Dai C Fan Y Liu X Bao X Yao Y Wang R . Predictors of immediate remission after surgery in cushing’s disease patients: a large retrospective study from a single center. Neuroendocrinology (2020) 111(11):1141–1150. doi: 10.1159/000509221 32512562

[23] Alexandraki KI Kaltsas GA Isidori AM Storr HL Afshar F Sabin I . Long-term remission and recurrence rates in cushing’s disease: predictive factors in a single-centre study. Eur J Endocrinol (2013) 168(4):639–48. doi: 10.1530/EJE-12-0921 23371975

[24] Chandler WF Barkan AL Hollon T Sakharova A Sack J Brahma B . Outcome of transsphenoidal surgery for cushing disease: A single-center experience over 32 years. Neurosurgery (2016) 78(2):216–23. doi: 10.1227/NEU.0000000000001011 26348007

[25] Ironside N Chatain G Asuzu D Benzo S Lodish M Sharma S . Earlier post-operative hypocortisolemia may predict durable remission from cushing’s disease. Eur J Endocrinol (2018) 178(3):255–63. doi: 10.1530/EJE-17-0873 PMC5812811 29330227

[26] Pendharkar AV Sussman ES Ho AL Gephart MGH Katznelson L . Cushing’s disease: predicting long-term remission after surgical treatment. Neurosurg Focus (2015) 38(2):E13. doi: 10.3171/2014.10.FOCUS14682 25639315

[27] Lindsay JR Oldfield EH Stratakis CA Nieman LK . The postoperative basal cortisol and CRH tests for prediction of long-term remission from cushing’s disease after transsphenoidal surgery. J Clin Endocrinol Metab (2011) 96(7):2057–64. doi: 10.1210/jc.2011-0456 PMC3135190 21508126

[28] Yap LB Turner HE Adams CBT Wass J a. H . Undetectable postoperative cortisol does not always predict long-term remission in cushing’s disease: a single centre audit*. Clin Endocrinol (Oxf) (2002) 56(1):25–31. doi: 10.1046/j.0300-0664.2001.01444.x 11849243

[29] Liu Y Liu X Hong X Liu P Bao X Yao Y . Prediction of recurrence after transsphenoidal surgery for cushing’s disease: The use of machine learning algorithms. Neuroendocrinology (2019) 108(3):201–10. doi: 10.1159/000496753 30630181

[30] Fan Y Li D Liu Y Feng M Chen Q Wang R . Toward better prediction of recurrence for cushing’s disease: a factorization-machine based neural approach. Int J Mach Learn Cybern (2020) 30(12):625–623. doi: 10.1007/s13042-020-01192-6

[31] Moreno-Moreno P Ibáñez-Costa A Venegas-Moreno E Fuentes-Fayos AC Alhambra-Expósito MR Fajardo-Montañana C . Integrative clinical, radiological, and molecular analysis for predicting remission and recurrence of cushing disease. J Clin Endocrinol Metab (2022) 107(7):e2938–51. doi: 10.1210/clinem/dgac172 35312002

[32] MacFarlane J Bashari WA Senanayake R Gillett D van der Meulen M Powlson AS . Advances in the imaging of pituitary tumors. Endocrinol Metab Clin (2020) 49(3):357–73. doi: 10.1016/j.ecl.2020.06.002 32741476

[33] Berkmann S Roethlisberger M Mueller B Christ-Crain M Mariani L Nitzsche E . Selective resection of cushing microadenoma guided by preoperative hybrid 18-fluoroethyl-L-tyrosine and 11-c-methionine PET/MRI. Pituitary (2021) 24(6):878–86. doi: 10.1007/s11102-021-01160-5 34155554

[34] Feng Z He D Mao Z Wang Z Zhu Y Zhang X . Utility of 11C-methionine and 18F-FDG PET/CT in patients with functioning pituitary adenomas. Clin Nucl Med (2016) 41(3):e130. doi: 10.1097/RLU.0000000000001085 26646998

[35] Sagan KP Andrysiak-Mamos E Sagan L Nowacki P Małkowski B Syrenicz A . Cushing’s syndrome in a patient with rathke’s cleft cyst and ACTH cell hyperplasia detected by 11C-methionine PET imaging–a case presentation. Front Endocrinol (2020) 11:460. doi: 10.3389/fendo.2020.00460/full PMC7388627 32774326

[36] Koulouri O Steuwe A Gillett D Hoole AC Powlson AS Donnelly NA . A role for 11C-methionine PET imaging in ACTH-dependent cushing’s syndrome. Eur J Endocrinol (2015) 173(4):M107–20. doi: 10.1530/EJE-15-0616 26245763

[37] Truong HQ Lieber S Najera E Alves-Belo JT Gardner PA Fernandez-Miranda JC . The medial wall of the cavernous sinus. part 1: Surgical anatomy, ligaments, and surgical technique for its mobilization and/or resection. J Neurosurg (2018) 131(1):122–30. doi: 10.3171/2018.3.JNS18596 30192192

