# Supplementary material for: The clinical outcomes of imaging modalities for surgical management Cushing’s disease – A systematic review and meta-analysis
Source: Front Endocrinol (Lausanne). 2023 Jan 13;13:1090144. doi: 10.3389/fendo.2022.1090144 (PMC9880448; doi:10.3389/fendo.2022.1090144)
Supplement: Supplementary file 1 [file DataSheet_1.docx]

Supplementary Tables

**Table of Contents**

[Supplementary Table 1. Search strategy. 3](#__RefHeading___Toc119877_1323829561)

[Supplementary Table 2. Characteristics of included studies that reported both remission and recurrence 4](#__RefHeading___Toc119879_1323829561)

[Supplementary Table 3. Characteristics of included studies that reported only remission. 11](#__RefHeading___Toc119881_1323829561)

[Supplementary Table 4. Characteristics of included studies that reported only recurrence. 15](#__RefHeading___Toc119883_1323829561)

[Supplementary Table 5. Quality Assessment 30](#__RefHeading___Toc119885_1323829561)

[Supplementary Table 6. All remission models for Remission 34](#__RefHeading___Toc119887_1323829561)

[Supplementary Table 7. Remission rates of the Octreotide best model 35](#__RefHeading___Toc119889_1323829561)

[Supplementary Table 8. Baseline models only for Remission 36](#__RefHeading___Toc119891_1323829561)

[Supplementary Table 9. Imaging Types for Remission 37](#__RefHeading___Toc119893_1323829561)

[Supplementary Table 10. MRI Sequences for Remission 38](#__RefHeading___Toc119895_1323829561)

[Supplementary Table 11. Year models for Remission 39](#__RefHeading___Toc119897_1323829561)

[Supplementary Table 12. All remission models for recurrence 40](#__RefHeading___Toc119899_1323829561)

[Supplementary Table 13. Baseline models only for recurrence 41](#__RefHeading___Toc119901_1323829561)

[Supplementary Table 14. Imaging Types in Recurrence 42](#__RefHeading___Toc119903_1323829561)

[Supplementary Table 15. MRI Sequences in Recurrence 43](#__RefHeading___Toc119905_1323829561)

[Supplementary Table 16. Year models for Recurrence 44](#__RefHeading___Toc119907_1323829561)

[Supplementary Table 17. Imaging Types without Macro for Remission 45](#__RefHeading___Toc119909_1323829561)

[Supplementary Table 18. Imaging Types without Undetect for Remission 46](#__RefHeading___Toc119911_1323829561)

[Supplementary Table 19. Imaging Types without Macro or Undetect for Remission 47](#__RefHeading___Toc119913_1323829561)

[Supplementary Table 20. MRI Sequences without Macro for Remission 48](#__RefHeading___Toc119915_1323829561)

[Supplementary Table 21. MRI Sequences without Undetect for Remission 49](#__RefHeading___Toc119917_1323829561)

[Supplementary Table 22. MRI Sequences without Macro or Undetect for Remission 50](#__RefHeading___Toc119919_1323829561)

[Supplementary Table 23. Year without Macro for Remission 51](#__RefHeading___Toc119921_1323829561)

[Supplementary Table 24. Year without Undetect for Remission 52](#__RefHeading___Toc119923_1323829561)

[Supplementary Table 25. Year without Macro or Undetect for Remission 53](#__RefHeading___Toc119925_1323829561)

[Supplementary Table 26. Imaging Types without Macro for Recurrence 54](#__RefHeading___Toc119927_1323829561)

[Supplementary Table 27. Imaging Types without Undetect for Recurrence 55](#__RefHeading___Toc119929_1323829561)

[Supplementary Table 28. Imaging Types without Macro or Undetect for Recurrence 56](#__RefHeading___Toc119931_1323829561)

[Supplementary Table 29. MRI Sequences without Macro for Recurrence 57](#__RefHeading___Toc119933_1323829561)

[Supplementary Table 30. MRI Sequences without Undetect for Recurrence 58](#__RefHeading___Toc119935_1323829561)

[Supplementary Table 31. MRI Sequences without Macro or Undetect for Recurrence 59](#__RefHeading___Toc119937_1323829561)

[Supplementary Table 32. Year without Macro for Recurrence 60](#__RefHeading___Toc119939_1323829561)

[Supplementary Table 33. Year without Undetect for Recurrence for Recurrence 61](#__RefHeading___Toc119941_1323829561)

[Supplementary Table 34. Year without Macro or Undetect for Recurrence 62](#__RefHeading___Toc119943_1323829561)

#### Supplementary Table 1. Search strategy.

| PICO | Terms |
| --- | --- |
| Population | (Cushing OR Cushing* OR ACTH OR ACTHoma OR ACTH* OR hypercortisolism OR hypercortisol*) AND  (surgery OR surgical OR surg* OR operation OR  operative OR transsphenoidal OR transsphenoid* OR  endonasal OR endoscopic OR endoscop*) |
| Intervention | (IPSS OR "inferior petrosal sinus sampling" OR  imaging OR MRI OR ultrasound OR CT OR PET OR  "positron emission tomography" OR hyperspectral  OR HSI OR fluorescence OR spectrometry OR spectroscopy  OR iMRI OR intraoperative MRI OR CT OR PET OR  "positron emission tomography" OR hyperspectral  OR HSI OR fluorescence OR spectrometry OR spectroscopy) |
| Control | Not searched |
| Outcome | Not searched |

#### Supplementary Table 2. Characteristics of included studies that reported both remission and recurrence

| Author | Year | Reference | Subgroup Description | Total N | Average follow-up (months) | Age (Years) | Male | Previous Surgery | Macroadenoma | Undetectable adenoma | CSI | Advanced MRI Sequences | Non-MRI Imaging |
| --- | --- | --- | --- | --- | --- | --- | --- | --- | --- | --- | --- | --- | --- |
| Kang | 2022 | 1 | 1.5T MRI | 11 | 87.3 | 13.6 | 55% | 0% | 9% | 18% | ? |  |  |
|  |  |  | 3T MRI | 7 | 53.1 | 14.9 | 43% | 0% | 0% | 14% | 0% | 3T |  |
| Berkmann | 2021 | 2 | FET-PET | 6 |  | 44 | 17% | 33% | 0% | 17% | 0% | GRE DMRI 3T | PET (100%) |
|  |  |  | MET-PET | 6 |  | 51.1 | 0% | 17% | 0% | 33% | 0% | GRE DMRI 3T | PET (100%) |
|  |  |  | MET-PET and FET-PET | 3 |  | 45.7 | 0% | 0% | 0% | 33% | 0% | GRE DMRI 3T | PET (100%) |
| Moszczyńska | 2021 | 3 |  | 12 | 10.1 | 11 | 50% | 0% | 0% | 83% | 0% | DMRI |  |
| Bora | 2020 | 4 |  | 85 | 23.5 | 28 | 29% | 0% | 26% | 7% | 2% | DMRI |  |
| Guaraldi | 2020 | 5 | Microadenoma | 80 | 92.3 | 39.7 | 29% | 11% | 0% | 0% | 0% | 3T |  |
|  |  |  | Undetectable adenoma | 36 | 92.3 | 39.7 | 29% | 11% | 0% | 100% | 0% | 3T |  |
| Parksook W.W. | 2020 | 6 | Macroadenoma | 11 | 42 | 33 | 20% | 0% | 100% | 0% | ? |  |  |
|  |  |  | Micro and undetectable adenoma | 34 | 42 | 33 | 20% | 0% | 0% | ? | ? |  |  |
| Serban | 2020 | 7 | First surgery; macroadenoma | 9 | 67.2 | 40.4 | 20% | 0% | 100% | 0% | 22% |  |  |
|  |  |  | First surgery; microadenoma | 49 | 67.2 | 40.4 | 20% | 0% | 0% | 0% | 0% |  |  |
|  |  |  | First surgery; undetectable adenoma | 15 | 67.2 | 40.4 | 20% | 0% | 0% | 100% | 0% |  |  |
|  |  |  | Repeat surgery; macroadenoma | 4 | 24 | 36.3 | 6% | 100% | 100% | 0% | ? |  |  |
|  |  |  | Repeat surgery; microadenoma | 11 | 24 | 36.3 | 6% | 100% | 0% | 0% | 0% |  |  |
|  |  |  | Repeat surgery; undetectable adenoma | 3 | 24 | 36.3 | 6% | 100% | 0% | 100% | ? |  |  |
| Strange | 2020 | 8 |  | 10 | 68.5 | 41 | 0% | 0% | 30% | ? | 10% |  | Intraop MRI (100%) |
| Broersen | 2019 | 9 | Microscopic TSS | 87 | 202.8 | 38.9 | 21% | 5% | 21% | 36% | 6% |  | Pre-CT modality (4%) CT (30%) N |
|  |  |  | Endoscopic TSS | 50 | 56.4 | 44.4 | 32% | 12% | 32% | ? | 16% | 3T | CT (2%) N |
| Chen | 2019 | 10 |  | 79 | 26 | 44 | 27% | 0% | 18% | 16% | ? | DMRI | CT (3%) |
| Doğanşen | 2019 | 11 | DGCT | 29 | 96 | 34 | 21% | ? | 34% | ? | ? |  |  |
|  |  |  | SGCT | 10 | 74 | 38 | 20% | ? | 70% | ? | ? |  |  |
| Liu Y. | 2019 | 12 |  | 504 | *43.8 | ? | ? | ? | ? | ? | ? | DMRI |  |
| Nadezhdina | 2019 | 13 |  | 498 | ? | 38 | 15% | 15% | 27% | 12% | ? |  |  |
| Todeschini | 2019 | 14 |  | 16 | 52 | 34.5 | 12% | 0% | 38% | 6% | 12% |  |  |
| ZieliÅ„ski | 2019 | 15 |  | 3 | 74.2 | 41 | 0% | ? | 33% | 33% | 0% | GRE 3T |  |
| Brichard | 2018 | 16 | Macroadenoma | 13 | 101.5 | 38 | 20% | ? | 100% | 0% | 23% |  | NAIntraop MRI (15%) |
|  |  |  | Microadenoma | 41 | 82 | 43 | 20% | ? | 0% | 0% | 0% |  | NAIntraop MRI (10%) |
|  |  |  | Nonvisible Adenoma | 17 | 69 | 46 | 20% | ? | 0% | 100% | 0% |  | N |
| Ironside N. | 2018 | 17 |  | 268 | 14 | ? | ? | 9% | ? | ? | ? |  |  |
| Mastorakos P. | 2018 | 18 |  | 115 | ? | 39.4 | 21% | ? | 27% | 0% | 0% | GRE |  |
| Mayberg | 2018 | 19 |  | 81 | 49.3 | 38.1 | 17% | 25% | 4% | 44% | 0% |  |  |
| Mortini P. | 2018 | 20 | Macroadenoma | 106 | 50.5 | 40.1 | 21% | 0% | 100% | 0% | 24% |  | N |
|  |  |  | Micro and undetectable adenoma | 388 | 50.5 | 40.1 | 21% | 0% | 0% | ? | 0% |  | N |
| Palejwala | 2018 | 21 | Not reproductive age CD | 23 | 50 | 55 | 0% | 4% | 17% | 17% | 26% | GRE DMRI |  |
|  |  |  | Reproductive age, non-pregnancy CD | 30 | 50 | 35 | 0% | 43% | 20% | 23% | 37% | GRE |  |
|  |  |  | Pregnancy associated CD | 11 | 29 | 34 | 0% | 36% | 36% | 0% | 27% | GRE |  |
| Qiao | 2018 | 22 | >60yo | 45 | 38.5 | 67.4 | 27% | 0% | 13% | 44% | 2% |  |  |
|  |  |  | <60yo | 90 | 46.9 | 40.8 | 12% | 0% | 17% | 42% | 7% |  |  |
| Selek A. | 2018 | 23 | Overall | 119 | 32.5 | 39.8 | 19% | ? | 45% | ? | 18% |  |  |
| Bansal | 2017 | 24 |  | 230 | 74 | 26.8 | 27% | ? | 27% | 17% | 10% | GRE DMRI |  |
| Cebula | 2017 | 25 | MRI positive | 160 | 21 | 43 | 15% | 16% | 34% | 0% | 31% |  |  |
|  |  |  | MRI negative | 70 | 21 | 41.1 | 26% | 7% | 0% | 100% | 13% |  |  |
| Johnston | 2017 | 26 | Macroadenoma | 27 | 51.6 | 47 | 28% | ? | 100% | 0% | 22% | GRE 3T |  |
|  |  |  | Microadenoma/undetectable | 74 | 51.6 | 47 | 28% | ? | 0% | 34% | 0% | GRE 3T |  |
| Keskin | 2017 | 27 | Macroadenoma | 19 | 90 | 44.3 | 23% | 30% | 100% | 0% | 47% |  |  |
|  |  |  | Microadenoma | 50 | 90 | 44.3 | 23% | 30% | 0% | 0% | 0% |  |  |
|  |  |  | Undetectable adenoma | 8 | 90 | 44.3 | 23% | 30% | 0% | 100% | 0% |  |  |
| Powell | 2017 | 28 |  | 32 | ? | ? | ? | 16% | ? | ? | ? |  |  |
| Shin | 2017 | 29 |  | 49 | 50 | 50 | 22% | 18% | 26% | 20% | 12% |  |  |
| Chandler | 2016 | 30 | Macroadenoma | 35 | 80.4 | 39.9 | 22% | 0% | 100% | 0% | ? |  |  |
|  |  |  | Microadenoma | 137 | 80.4 | 39.9 | 22% | 0% | 0% | 0% | ? |  |  |
|  |  |  | Undetectable | 103 | 80.4 | 39.9 | 22% | 0% | 0% | 100% | ? |  | CT (27%) |
| Chen Y. | 2016 | 31 | Macroadenoma | 20 | 20.8 | 38.8 | 10% | 0% | 100% | 0% | 35% | DMRI |  |
|  |  |  | Microadenoma | 126 | 20.8 | 36.9 | ? | 0% | 0% | 0% | 0% | DMRI |  |
| Machado | 2016 | 32 | Microadenoma | 203 | 68 | 31 | 22% | ? | 0% | ? | 0% | GRE DMRI |  |
|  |  |  | Macroadenoma | 61 | 54 | 31 | 9% | ? | 100% | 0% | 23% | GRE DMRI |  |
| Sarkar | 2016 | 33 | No PET | 56 | 20 | 31.9 | 20% | 9% | 17% | 12% | ? | GRE DMRI 3T |  |
|  |  |  | PET only | 3 |  | 31.9 | 20% | 9% | 17% | 12% | ? | GRE DMRI 3T | PET (100%) |
| Shirvani | 2016 | 34 |  | 96 | 44 | 31.4 | 24% | ? | 19% | 12% | 2% | DMRI |  |
| Solak | 2016 | 35 |  | 33 | 28 | 38 | 18% | ? | 30% | 15% | 12% |  |  |
| Amlashi | 2015 | 36 | Macroadenoma | 10 | 53.5 | 42.4 | 17% | ? | 100% | 0% | ? |  |  |
|  |  |  | Microadenoma+Undetectable | 79 | 53.5 | 42.4 | 17% | ? | 0% | ? | ? |  |  |
| Kuo | 2015 | 37 |  | 40 | 40.2 | 41 | 20% | 0% | 45% | 0% | 20% |  |  |
| Yamada | 2015 | 38 | Macroadenoma | 76 | 72.5 | 42.7 | 15% | ? | 100% | 0% | ? | GRE DMRI 3T |  |
|  |  |  | Microadenoma | 154 | 72.5 | 42.7 | 15% | ? | 0% | 0% | ? | GRE DMRI 3T |  |
|  |  |  | Undetectable | 22 | 72.5 | 42.7 | 15% | ? | 0% | 100% | ? | GRE DMRI 3T |  |
| Berker M. | 2014 | 39 | Macroadenoma, primary surgery | 26 | 32 | 38.7 | ? | 0% | 100% | 0% | ? |  |  |
|  |  |  | Microadenoma, primary surgery | 43 | 32 | 38.7 | ? | 0% | 0% | ? | ? |  |  |
|  |  |  | Macroadenoma, secondary surgery | 3 | 32 | 38.7 | ? | 100% | 5% | 0% | ? |  |  |
|  |  |  | Microadenoma, secondary surgery | 18 | 32 | 38.7 | ? | 100% | 0% | ? | ? |  |  |
| Dimopoulou | 2014 | 40 | Macroadenoma | 32 | 62 | 50 | 20% | 0% | 100% | 0% | 25% | DMRI |  |
|  |  |  | Microadenoma | 58 | 62 | 50 | 20% | 0% | 0% | 0% | 9% | DMRI |  |
|  |  |  | Undetectable adenoma | 30 | 62 | 50 | 20% | 0% | 0% | 100% | 0% | DMRI |  |
| Mehta | 2014 | 41 |  | 78 | 49.2 | 45.5 | 19% | 0% | 0% | 56% | 10% | GRE |  |
|  |  |  | Not empty sella syndrome | 78 | 57.6 | 45 | 21% | 0% | 0% | 56% | 15% | GRE |  |
| Paluzzi A. | 2014 | 42 | Macroadenoma | 21 | 37.2 | ? | ? | ? | 100% | 0% | 9% |  |  |
|  |  |  | Microadenoma | 36 | 37.2 | ? | ? | ? | 0% | ? | 0% |  |  |
| Storr | 2014 | 43 |  | 6 | 55 | 14.9 | 83% | 33% | 17% | 50% | ? | DMRI |  |
| Valderrábano | 2014 | 44 |  | 26 | 139 | 37.1 | 19% | 100% | 15% | 46% | ? |  | CT (12%) |
| Alahmadi | 2013 | 45 | Endoscopic TSS for microadenoma | 10 | 33 | 50 | 26% | ? | 0% | ? | ? | DMRI |  |
|  |  |  | Endoscopic TSS for macroadenoma | 7 | 33 | 50 | 26% | ? | 100% | 0% | ? | DMRI |  |
|  |  |  | Microscopic TSS for microadenoma | 17 | 33 | 50 | 26% | ? | 0% | ? | ? | DMRI |  |
|  |  |  | Microscopic TSS for macroadenoma | 8 | 33 | 50 | 26% | ? | 100% | 0% | ? | DMRI |  |
| Alexandraki | 2013 | 46 | MRI macroadenoma | 15 | 184.8 | 39.2 | 22% | ? | 100% | ? | ? |  |  |
|  |  |  | MRI microadenoma | 58 | 184.8 | 39.2 | 22% | ? | 0% | ? | ? |  |  |
|  |  |  | CT | 51 |  | 39.2 | 22% | 0% | 12% | ? | ? |  | CT (100%) |
| Hameed | 2013 | 47 | Microadenoma + Undetectable adenoma | 36 | 16.5 | 45 | 27% | ? | 0% | 22% | ? |  |  |
|  |  |  | Macroadenoma | 16 | 16.5 | 45 | 27% | ? | 100% | 0% | 0% |  |  |
| Lampropoulos | 2013 | 48 | Macroadenoma | 9 | 43.2 | 46.6 | 9% | ? | 100% | 0% | ? |  |  |
|  |  |  | Microadenoma | 9 | 43.2 | 46.6 | 9% | ? | 0% | 0% | 0% |  |  |
|  |  |  | Undetectable adenoma | 5 | 43.2 | 46.6 | 9% | ? | 0% | 100% | 0% |  |  |
| Lonser | 2013 | 49 |  | 200 | 81.6 | 13.7 | 47% | 14% | ? | 52% | 4% | GRE | CT (4%) |
| Starke | 2013 | 50 | Macroadenoma | 15 | 21 | 39 | 15% | ? | 100% | 0% | 40% |  |  |
|  |  |  | Microadenoma | 30 | 21 | 39 | 15% | ? | 0% | 0% | 0% |  |  |
|  |  |  | Undetectable adenoma | 16 | 21 | 39 | 15% | ? | 0% | 100% | 0% |  |  |
| Wagenmakers | 2013 | 51 | Invasive macroadenoma (Knosp >=3) | 11 | 71 | 42.3 | 28% | 0% | 100% | 0% | 100% |  |  |
|  |  |  | Non-invasive macroadenoma (Knosp <=2) | 20 | 71 | 42.3 | 28% | 0% | 100% | 0% | 0% |  |  |
|  |  |  | Microadenoma | 35 | 71 | 42.3 | 28% | 0% | 0% | 0% | 0% |  |  |
|  |  |  | Undetectable adenoma | 20 | 71 | 42.3 | 28% | 0% | 0% | 100% | 0% |  |  |
| Ciric | 2012 | 52 | Macroadenoma | 13 | 67 | 41 | 21% | ? | 100% | 0% | ? |  |  |
|  |  |  | Microadenoma | 82 | 70 | 41 | 21% | ? | 0% | 0% | ? |  |  |
|  |  |  | Undetectable adenoma | 23 | 70 | 41 | 21% | ? | 0% | 100% | ? |  |  |
| Honegger | 2012 | 53 | Microadenoma | 63 | 38.2 | 46 | 27% | ? | 0% | 0% | ? |  |  |
|  |  |  | Undetectable adenoma | 9 | 38.2 | 46 | 27% | ? | 0% | 100% | ? |  |  |
|  |  |  | Macroadenoma | 11 | 38.2 | 46 | 27% | ? | 100% | 0% | 64% |  |  |
| Kim | 2012 | 54 |  | 54 | 102.4 | 35.7 | 24% | 0% | ? | ? | ? |  | CT (4%) |
| Lonser | 2012 | 55 |  | 87 | 20.4 | 31.7 | 33% | ? | ? | ? | 7% | GRE |  |
| Sun | 2012 | 56 | MRI positive | 95 | 37.4 | 36 | 27% | ? | 6% | 0% | ? | DMRI |  |
|  |  |  | MRI negative | 24 | 40.6 | 36.5 | 25% | ? | 0% | 100% | ? | DMRI |  |
| Fan C. | 2011 | 57 |  | 108 | 36.3 | 35.1 | 72% | 0% | 7% | 13% | ? | DMRI |  |
| Knappe | 2011 | 58 |  | 18 |  | 42 | 22% | 11% | 6% | 22% | ? | DMRI | Intraop US (100%) |
| Storr | 2011 | 59 | Paediatric - majority are MRI | 37 | ? | 12.3 | 37% | ? | 2% | 48% | ? |  | CT (5%) |
|  |  |  | Adult - majority were CTs | 129 |  | 40 | 21% | ? | 15% | 57% | ? |  | CT (64%) |
| Jagannathan | 2009 | 60 | Macro+microadenoma | 132 | 84 | 32.5 | 24% | 0% | 15% | 0% | ? | GRE |  |
|  |  |  | Undetectable adenoma | 126 | 84 | 32.5 | 24% | 0% | 0% | 100% | ? | GRE |  |
| Wagenmakers | 2009 | 61 |  | 14 | 37.8 | ? | ? | 100% | ? | ? | ? |  |  |
| Atkinson | 2008 | 62 | Microscopic | 21 | 12 | 45 | 19% | 0% | 0% | 0% | 0% |  |  |
|  |  |  | Endoscopic | 21 | 30 | 43.8 | 24% | 0% | 0% | 0% | 0% |  |  |
| Jehle | 2008 | 63 |  | 193 | 57.6 | 40.9 | 18% | 0% | 16% | 51% | ? | DMRI |  |
| Prevedello | 2008 | 64 |  | 167 | 39 | 42.3 | 18% | 0% | 0% | 0% | 0% |  |  |
| Acebes | 2007 | 65 |  | 44 | 49 | 41.5 | 11% | 0% | 23% | 16% | 9% |  |  |
| Das | 2007 | 66 | Macroadenoma | 3 | 64 | 16.7 | 67% | ? | 100% | 0% | 0% | DMRI |  |
|  |  |  | Microadenoma | 6 | 64 | 15.2 | 60% | ? | 0% | 0% | 0% | DMRI |  |
|  |  |  | Undetectable adenoma | 1 | 64 | 13.5 | 100% | ? | 0% | 0% | 0% | DMRI |  |
| Dehdashti | 2007 | 67 |  | 25 | 17 | 42 | 24% | 12% | 28% | 20% | 8% | DMRI 3T |  |
| Rollin | 2007 | 68 | Macroadenoma - first surgery | 23 | 72 | 37 | 23% | 0% | 100% | 0% | ? |  |  |
|  |  |  | Microadenoma - first surgery | 59 | 72 | 37 | 23% | 0% | 0% | 0% | ? |  |  |
|  |  |  | Undetectable adenoma - first surgery | 21 | 72 | 37 | 23% | 0% | 0% | 100% | ? |  |  |
|  |  |  | Second surgery only | 14 | 72 | 37 | 23% | 100% | ? | ? | ? |  |  |
| Testa | 2007 | 69 | Macro+micro adenoma | 16 | 38.4 | 40.1 | 19% | ? | 6% | 0% | ? | DMRI |  |
|  |  |  | Undetectable adenoma | 15 | 38.4 | 40.1 | 19% | ? | 0% | 100% | ? | DMRI |  |
| Esposito | 2006 | 70 | Overall | 40 | 33 | 39 | 8% | 28% | 21% | 21% | ? | GRE DMRI |  |
| Hofmann | 2006 | 71 |  | 16 | 23 | 38.2 | 12% | 100% | 12% | 88% | ? |  |  |
| Batista | 2005 | 72 |  | 30 | ? | 12 | 57% | ? | 7% | 33% | ? | GRE |  |
| Joshi | 2005 | 73 |  | 25 | 59.5 | 13.4 | 60% | ? | ? | ? | ? |  | CT (8%) |
| Kanter | 2005 | 74 |  | 33 | 44 | 13 | 48% | 21% | 12% | 33% | ? |  |  |
| Höybye | 2004 | 75 |  | 34 | 72 | 40 | 24% | 0% | 6% | 65% | ? |  |  |
| Mehrazin M. | 2004 | 76 |  | 11 |  | 21.5 | 27% | ? | 36% | 18% | ? |  | CT (100%) |
| Salenave | 2004 | 77 | Undetectable adenoma | 27 | 21 | 38.4 | 29% | 0% | 0% | 100% | 0% | DMRI |  |
|  |  |  | Microadenoma | 25 | 18.1 | 40.4 | 8% | 0% | 0% | 0% | 0% | DMRI |  |
| Cannavò | 2003 | 78 | No CSI | 18 | 78 | 43.4 | 44% | 0% | 100% | 0% | 0% |  |  |
|  |  |  | CSI | 8 | 78 | 43.4 | 100% | 0% | 100% | 0% | 100% |  |  |
| Zhao | 2003 | 79 | Microadenoma | 46 | 100 | 33 | 23% | ? | 0% | 0% | ? |  | CT (24%) |
|  |  |  | Undetectable adenoma | 41 | 100 | 33 | 23% | ? | 0% | 100% | ? |  | CT (24%) |
| Kristof | 2002 | 80 | Macroadenoma | 6 | ? | 32 | 26% | ? | 100% | 0% | 50% |  |  |
|  |  |  | Micro + Undetectable adenoma | 21 | ? | 32 | 26% | ? | 0% | ? | ? |  |  |
| Rees | 2002 | 81 |  | 53 | 72 | 41.3 | 22% | 0% | 19% | 38% | ? |  | Pre-CT modality (7%) CT (20%) |
| Shimon | 2002 | 82 | First surgery | 74 | 50.4 | 39 | 13% | 0% | 4% | ? | ? |  |  |
|  |  |  | Second surgery | 13 | 50.4 | 39 | 13% | 100% | 0% | ? | ? |  |  |
| Yap | 2002 | 83 |  | 89 |  | 39.1 | 20% | ? | 10% | 50% | ? |  | Pre-CT modality (5%) CT (60%) |
| Ohnishi | 2000 | 84 | Overall | 30 | 115 | 34 | 23% | ? | ? | 33% | ? | DMRI | CT (37%) |
| Semple | 2000 | 85 |  | 17 | 21.6 | 47.8 | 28% | 0% | 0% | 100% | 0% |  |  |
| Sheehan | 2000 | 86 |  | 29 | 38 | ? | ? | 10% | ? | 28% | ? |  |  |
| Vallette-Kasic | 2000 | 87 |  | 53 | 38.4 | 39 | 8% | ? | 0% | 6% | 13% |  | CT (4%) |
| Flitsch J | 1999 | 88 |  | 22 | ? | ? | ? | ? | 23% | 45% | ? |  |  |
| Gsponer | 1999 | 89 |  | 15 | ? | 44.6 | 7% | ? | 20% | 53% | ? |  |  |
| Devoe | 1997 | 90 |  | 33 |  | 13.1 | 40% | 0% | ? | 43% | ? |  | Pre-CT modality (5%) CT (52%) |
| Knappe | 1996 | 91 |  | 55 | 54.5 | 14.4 | 47% | 24% | ? | 52% | 4% |  | CT (39%) |
| Oliverio | 1996 | 92 |  | 17 | ? | 31 | 41% | 6% | ? | 59% | ? |  |  |
| Weber | 1995 | 93 |  | 9 |  | 13.7 | 56% | 0% | 0% | 56% | ? |  | CT (100%) |
| Dyer | 1994 | 94 |  | 33 |  | ? | 58% | ? | 0% | 86% | ? |  | CT (75%) |
| Magiakou | 1994 | 95 | First surgery | 37 | 22 | 14 | 38% | 0% | ? | 48% | 4% |  |  |
|  |  |  | Second surgery | 12 | 22 | 14 | 38% | 100% | ? | 48% | 4% |  |  |
| Robert | 1991 | 96 | No imaging | 55 |  | 29.1 | 27% | 9% | 0% | 100% | ? |  | Pre-CT modality (100%) |
|  |  |  | CT imaging | 33 |  | 34.2 | 27% | 0% | 9% | 33% | ? |  | CT (100%) |
| Friedman | 1989 | 97 |  | 33 |  | 37.2 | 27% | 94% | ? | 91% | ? |  | CT (100%) |
| Mampalam | 1988 | 98 |  | 216 |  | 35 | 19% | 4% | ? | 35% | ? |  | CT (100%) |
| Styne | 1984 | 99 |  | 15 |  | 13.1 | 53% | ? | ? | ? | ? |  | Pre-CT modality (20%) CT (80%) |
| Wajchenberg | 1979 | 100 |  | 6 |  | 36 | 0% | ? | 0% | 100% | ? |  | Pre-CT modality (100%) |

#### Supplementary Table 3. Characteristics of included studies that reported only remission.

| Author | Year | Reference | Subgroup Description | Total N | Average follow-up (months) | Age (Years) | Male | Previous Surgery | Macroadenoma | Undetectable adenoma | CSI | Advanced MRI Sequences | Non-MRI Imaging |
| --- | --- | --- | --- | --- | --- | --- | --- | --- | --- | --- | --- | --- | --- |
| Dai | 2022 | 101 |  | 856 | ? | 40 | 18% | 10% | ? | 9% | 15% | DMRI 3T | NANAN |
| Scherer | 2022 | 102 |  | 23 |  | 42.5 | ? | 9% | 30% | 0% | ? | 3T | Intraop MRI (100%) |
| Wu | 2022 | 103 | Microadenoma | 36 |  | 33.1 | 22% | ? | 0% | 0% | 0% | 3T |  |
|  |  |  | Undetectable adenoma | 9 | ? | 40 | 22% | ? | 0% | 100% | 0% | 3T |  |
| Bestepe | 2021 | 104 |  | 35 | ? | 46.7 | 17% | 0% | 0% | 34% | 0% |  |  |
| Tatsi | 2021 | 105 | Undetectable adenoma | 59 | ? | 11.1 | 51% | 0% | 0% | 100% | 0% | GRE DMRI 3T |  |
|  |  |  | Detectable adenoma | 127 | ? | 14.4 | 40% | 0% | ? | 0% | ? | GRE DMRI 3T |  |
| Juthani R.G. | 2020 | 106 | Endoscopic surgery only | 10 |  | ? | ? | ? | ? | ? | ? |  | Intraop MRI (100%) |
|  |  |  | Microscopic surgery only | 9 |  | ? | ? | ? | ? | ? | ? |  | Intraop MRI (100%) |
| Walia | 2020 | 107 |  | 24 |  | 37.4 | 38% | ? | 25% | 17% | ? | GRE 3T | PET (100%) |
| Zhu J. | 2020 | 108 |  | 4 |  | 26.8 | 50% | ? | 25% | 0% | ? | DMRI | PET (0%) |
| Cristante | 2019 | 109 | Microadenoma | 89 | ? | 41 | 20% | 3% | 0% | 0% | ? | DMRI 3T |  |
|  |  |  | Macroadenoma | 18 | ? | 41 | 20% | 3% | 100% | 0% | ? | DMRI 3T |  |
|  |  |  |  | 88 | ? | 41 | 20% | 3% | 0% | 100% | ? | DMRI 3T |  |
| Gazioglu N. | 2019 | 110 |  | 10 | 132 | 14.8 | 43% | ? | 40% | 30% | ? | DMRI |  |
| Chatain | 2018 | 111 | 1.5T only | 8 | ? | 36.9 | 12% | 0% | 25% | 0% | ? | GRE |  |
|  |  |  | 3T MRI | 15 | ? | 25.9 | 40% | 0% | 7% | 0% | ? | GRE 3T |  |
| Cohen-Cohen | 2018 | 112 | pituitary adenoma with isolated invasion of the medial wall | 9 | 30 | ? | ? | 10% | ? | ? | ? |  |  |
| De Sousa S.M.C. | 2018 | 113 | Undetectable oadenoma | 2 | ? | 49.5 | 100% | ? | 0% | 100% | ? |  |  |
|  |  |  | Microadenoma | 9 | ? | 41.8 | 0% | ? | 0% | 0% | ? |  |  |
| Erfe | 2018 | 114 |  | 14 | 33.4 | 48.3 | 29% | 0% | 14% | ? | ? | DMRI 3T |  |
| Lang M. | 2018 | 115 |  | 12 | ? | 47.2 | 8% | ? | ? | 0% | ? | GRE |  |
| Lee J.Y.K | 2018 | 116 |  | 5 |  | 44.2 | 20% | ? | 80% | 0% | 20% |  | Intraop Fluorescence (100%) |
| Valassi E. | 2018 | 117 |  | 767 | 3.2 | ? | ? | ? | 23% | 22% | ? |  |  |
| Zaidi | 2018 | 118 | Detectable adenoma | 40 | 18.3 | 38.5 | 33% | 0% | ? | 0% | ? |  |  |
|  |  |  | Undetectable adenoma | 11 | 18.3 | 38.5 | 33% | 0% | 0% | 100% | ? |  |  |
| Masopust | 2017 | 119 |  | 41 |  | 40 | 73% | ? | 12% | ? | ? | GRE 3T | Intraop MRI (100%) |
| Witek | 2016 | 120 | Macroadenoma | 18 | ? | 46.6 | 25% | 30% | 100% | 0% | 50% |  |  |
|  |  |  | Microadenoma | 39 | ? | 43.2 | 18% | 3% | 0% | 0% | 0% |  |  |
| Afshari | 2015 | 121 |  | 28 | ? | 16.7 | 29% | 7% | ? | ? | ? |  |  |
| Chittiboina P | 2015 | 122 |  | 10 |  | 30.8 | 30% | ? | ? | 30% | ? | GRE | PET (100%) |
| Sun | 2015 | 123 | Undetectable | 3 |  | 41 | 22% | ? | 0% | 100% | 0% | DMRI | Intraop MRI (100%) |
| Potts M.B. | 2014 | 124 |  | 87 | 12 | 40.4 | 12% | 21% | ? | ? | ? | DMRI 3T |  |
| Burkhardt | 2013 | 125 | Macroadenoma | 4 | ? | 42 | 24% | 0% | 100% | 0% | ? |  |  |
|  |  |  | Microadenoma | 27 | ? | 42 | 24% | 19% | 0% | 0% | ? |  |  |
|  |  |  | Undetectable adenoma | 11 | ? | 42 | 24% | 19% | 0% | 100% | 0% |  |  |
| Kasaliwal | 2013 | 126 |  | 30 | ? | 27.8 | 29% | ? | 20% | 7% | ? | GRE DMRI |  |
| Tanei | 2013 | 127 |  | 3 |  | 39.3 | 0% | ? | 0% | 0% | 33% |  | Intraop MRI (100%) |
| Marić A. | 2012 | 128 |  | 4 | ? | 57 | 50% | 0% | 25% | ? | ? | DMRI |  |
| Sheth | 2012 | 129 |  | 42 | 30 | 41.5 | 20% | ? | 0% | 100% | 0% |  |  |
| Witek | 2012 | 130 | Macroadenoma | 6 | 18 | 36.3 | 17% | ? | 100% | 0% | ? |  |  |
|  |  |  | Microadenoma | 22 | 18 | 36.3 | 17% | ? | 0% | 0% | 0% |  |  |
|  |  |  | Undetectable adenoma | 8 | 18 | 36.3 | 17% | ? | 0% | 100% | ? |  |  |
| Witek | 2012 | 131 | Macroadenoma | 6 | 31 | 41.3 | 33% | 17% | 100% | 0% | ? |  |  |
|  |  |  | Microadenoma | 22 | 31 | 35.7 | 14% | 9% | 0% | 0% | ? |  |  |
|  |  |  | Undetectable adenoma | 8 | 27.8 | 34.3 | 12% | 62% | 0% | 100% | ? |  |  |
| Hofstetter | 2011 | 132 |  | 18 | 24.8 | 53.8 | 28% | ? | 39% | 22% | 17% |  |  |
| Lim | 2011 | 133 |  | 18 | 28.6 | 38.6 | 6% | 17% | ? | 39% | ? | DMRI 3T |  |
| Shah | 2011 | 134 | Macroadenoma | 10 | 59.2 | 14.8 | 60% | ? | 100% | 0% | ? |  |  |
|  |  |  | Microadenoma | 24 | 59.2 | 14.8 | 60% | ? | 0% | 0% | ? |  |  |
|  |  |  | Undetectable adenoma | 14 | 59.2 | 14.8 | 60% | ? | 0% | 100% | ? |  |  |
| Shi | 2011 | 135 |  | 58 | 29.1 | 36 | 25% | ? | ? | 48% | ? |  | N |
| Vitaz | 2011 | 136 |  | 7 |  | ? | ? | ? | ? | ? | ? |  | Intraop MRI (100%) |
| Dias | 2010 | 137 |  | 16 | 38.4 | 9.9 | 75% | 0% | 6% | 38% | ? | GRE DMRI |  |
| Oliveira | 2010 | 138 | MRI scans | 5 | 33.6 | 12.8 | 60% | ? | 0% | 40% | 0% |  |  |
|  |  |  | CT scans | 10 |  | 13.2 | 40% | ? | 20% | 30% | 20% |  | CT (100%) |
| Alzahrani A.S. | 2009 | 139 |  | 11 |  | 40 | 33% | 45% | 27% | 27% | ? |  | PET (100%) |
| Choe | 2008 | 140 |  | 6 | ? | 41.7 | 0% | ? | 33% | ? | ? | DMRI |  |
| Gazioglu | 2008 | 141 |  | 26 | ? | 39.6 | 12% | ? | 0% | 59% | ? | DMRI |  |
| Hofmann | 2008 | 142 | MRI | 270 | 72.3 | 39.4 | 24% | 0% | ? | 49% | ? |  |  |
|  |  |  | CT or X-ray | 156 |  | 39.4 | 24% | 0% | ? | ? | ? |  |  |
| Porterfield | 2008 | 143 | First surgery | 140 | ? | ? | ? | 0% | ? | ? | ? |  |  |
|  |  |  | Repeat surgery | 22 | ? | ? | ? | ? | ? | ? | ? |  |  |
| Zhang | 2008 | 144 |  | 15 | ? | ? | ? | ? | ? | ? | ? | DMRI |  |
| Locatelli | 2005 | 145 | Repeat operation | 12 | 24.5 | 34.6 | 25% | 100% | 0% | 92% | ? |  |  |
|  |  |  | Primary operation | 215 | ? | ? | ? | ? | ? | ? | ? |  |  |
| Storr | 2005 | 146 |  | 25 | 85.2 | 12.8 | 60% | ? | 4% | 40% | 0% |  |  |
| Thomale UW | 2005 | 147 |  | 4 | 0.2 | 43 | 50% | 25% | 0% | ? | ? | DMRI |  |
| Woo | 2005 | 148 |  | 16 | ? | 47.3 | 21% | 0% | 100% | 0% | 44% |  | CT (39%) |
| Lienhardt | 2001 | 149 |  | 9 | ? | 13.5 | 44% | 0% | ? | 56% | ? |  | CT (44%) |
| Graham | 1999 | 150 |  | 76 | 37 | 40.5 | 81% | 15% | ? | ? | ? |  |  |
| Booth | 1998 | 151 |  | 35 | ? | 36 | 16% | 6% | 0% | 27% | ? |  | CT (20%) |
| Cukiert A | 1998 | 152 |  | 4 | ? | ? | ? | ? | ? | ? | 100% |  |  |
| Mason | 1997 | 153 |  | 10 | 16.1 | 32.5 | 50% | 10% | 10% | 10% | ? |  |  |
| Doppman J.L. | 1994 | 154 |  | 13 |  | 40 | 8% | 0% | 23% | 38% | ? |  | Intraop US (100%) |
| Tripathi | 1994 | 155 | Macroadenoma | 2 | ? | 21 | 0% | ? | 100% | 0% | ? |  | CT (50%) |
|  |  |  | Microadenoma | 7 | ? | 31.1 | 0% | ? | 0% | 0% | ? |  |  |
|  |  |  | Undetectable adenoma | 2 | ? | 22.5 | 0% | ? | 0% | 100% | ? |  | CT (50%) |
| Escourolle | 1993 | 156 |  | 16 | ? | 42 | 14% | 12% | 38% | 12% | 6% |  |  |
| Buchfelder | 1992 | 157 | MRI Macroadenoma | 10 | ? | ? | ? | ? | 100% | 0% | 20% |  |  |
|  |  |  | MRI Microadenoma | 28 | ? | ? | ? | ? | 0% | 0% | 0% |  |  |
|  |  |  | MRI undetectable | 40 | ? | ? | ? | ? | 0% | 100% | 0% |  |  |
|  |  |  | CT only | 63 |  | ? | ? | ? | 8% | 53% | 1% |  | CT (100%) |
| Boolell | 1990 | 158 |  | 15 |  | 35.5 | 15% | ? | 0% | 73% | ? |  | CT (100%) |
| Merola | 1988 | 159 |  | 5 |  | 43.5 | 0% | ? | 0% | 100% | ? |  | CT (100%) |
| Tyrrell | 1978 | 160 |  | 20 |  | 32.8 | 15% | 0% | 0% | 40% | ? |  | Pre-CT modality (100%) |

#### Supplementary Table 4. Characteristics of included studies that reported only recurrence.

| Author | Year | Reference | Subgroup Description | Total N | Average follow-up (months) | Age (Years) | Male | Previous Surgery | Macroadenoma | Undetectable adenoma | CSI | Advanced MRI Sequences | Non-MRI Imaging |
| --- | --- | --- | --- | --- | --- | --- | --- | --- | --- | --- | --- | --- | --- |
| Mallari | 2022 | 161 |  | 76 |  | 43.6 | 14% | 29% | ? | 18% | 26% | 3T |  |
| Pappy | 2019 | 162 |  | 37 | 20.4 | 44.7 | 21% | ? | 36% | ? | 17% |  |  |
| Crock | 2018 | 163 | Series 2 only | 45 | ? | ? | ? | ? | ? | ? | ? |  |  |
| Harker | 2017 | 164 | Clear lateralisation of undetectable adenoma | 35 | 12 | 33.8 | 13% | 0% | 0% | 100% | 0% | DMRI 3T |  |
|  |  |  | No lateralisation of undetectable adenoma | 5 | 12 | 35.7 | 29% | 0% | 0% | 100% | 0% | DMRI 3T |  |
| Berkmann | 2012 | 165 |  | 4 |  | 35.9 | ? | ? | 14% | 43% | ? |  | Intraop MRI (57%) |
| Sheth S. | 2011 | 166 |  | 35 | 30 | 41.5 | 20% | ? | 0% | ? | ? |  |  |

**REFERENCES**

[1–100] [101–160] [161–165]

1. Kang KM, Muralidharan K, Knowlton H, et al (2022) Utility of bilateral inferior petrosal sinus sampling for diagnosis and lateralization of Cushing’s disease in the pediatric population: case series and review of the literature. J Endocrinol Invest 45:617–627. https://doi.org/10.1007/s40618-021-01680-8

2. Berkmann S, Roethlisberger M, Mueller B, et al (2021) Selective resection of cushing microadenoma guided by preoperative hybrid 18-fluoroethyl-L-tyrosine and 11-C-methionine PET/MRI. Pituitary 24:878–886. https://doi.org/10.1007/s11102-021-01160-5

3. Moszczyńska E, Marczak E, Szalecki M, et al (2021) The Effects of Sampling Lateralization on Bilateral Inferior Petrosal Sinus Sampling for Pediatric Cushing’s Disease—A Single Endocrinology Centre Experience and Review of the Literature. Front Endocrinol 12:

4. Bora SK, Suri A, Khadgawat R, et al (2020) Management of Cushing’s Disease: Changing Trend from Microscopic to Endoscopic Surgery. World Neurosurg 134:e46–e54. https://doi.org/10.1016/j.wneu.2019.08.165

5. Guaraldi F, Zoli M, Asioli S, et al (2020) Results and predictors of outcome of endoscopic endonasal surgery in Cushing’s disease: 20-year experience of an Italian referral Pituitary Center. J Endocrinol Invest 43:1463–1471. https://doi.org/10.1007/s40618-020-01225-5

6. Parksook WW, Laichuthai N, Sunthornyothin S (2020) Clinical Characteristics and Treatment Outcomes in Endogenous Cushing’s Syndrome: A 15-Year Experience from Thailand. Case Rep Endocrinol 2020:2946868. https://doi.org/10.1155/2020/2946868

7. Serban AL, Del Sindaco G, Sala E, et al (2020) Determinants of outcome of transsphenoidal surgery for Cushing disease in a single-centre series. J Endocrinol Invest 43:631–639. https://doi.org/10.1007/s40618-019-01151-1

8. Strange F, Remonda L, Schütz P, et al (2020) 10 Years’ Experience of Using Low-Field Intraoperative MRI in Transsphenoidal Surgery for Pituitary Adenoma: Results of the Swiss Pituitary Registry (SwissPit). World Neurosurg 136:e284–e293. https://doi.org/10.1016/j.wneu.2019.12.146

9. Broersen LHA, van Haalen FM, Biermasz NR, et al (2019) Microscopic versus endoscopic transsphenoidal surgery in the Leiden cohort treated for Cushing’s disease: surgical outcome, mortality, and complications. Orphanet J Rare Dis 14:64. https://doi.org/10.1186/s13023-019-1038-0

10. Chen S, Xu S, Lin F, et al (2019) Endoscopic surgical treatment of Cushing’s disease: A single-center experience of cauterization of peritumoral tissues. Exp Ther Med 18:4420–4426. https://doi.org/10.3892/etm.2019.8075

11. Doğanşen SÇ, Bilgiç B, Yalin GY, et al (2019) Clinical Significance of Granulation Pattern in Corticotroph Pituitary Adenomas. Turk Patoloji Derg 35:9–14. https://doi.org/10.5146/tjpath.2018.01434

12. Liu Y, Liu X, Hong X, et al (2019) Prediction of Recurrence after Transsphenoidal Surgery for Cushing’s Disease: The Use of Machine Learning Algorithms. Neuroendocrinology 108:201–210. https://doi.org/10.1159/000496753

13. Nadezhdina EY, Rebrova OYu, Grigoriev AY, et al (2019) Prediction of recurrence and remission within 3 years in patients with Cushing disease after successful transnasal adenomectomy. Pituitary 22:574–580. https://doi.org/10.1007/s11102-019-00985-5

14. Todeschini AB, Santos ARLD, Dolci RLL, et al (2019) Long Term Follow-up after Endoscopic Endonasal Approach for the Treatment of Cushing’s Disease. J Neurol Surg Part B Skull Base 80:306–309. https://doi.org/10.1055/s-0038-1669956

15. Zieliński G, Sajjad EA, Maksymowicz M, et al (2019) Double pituitary adenomas in a large surgical series. Pituitary 22:620–632. https://doi.org/10.1007/s11102-019-00996-2

16. Brichard C, Costa E, Fomekong E, et al (2018) Outcome of Transsphenoidal Surgery for Cushing Disease: A Single-Center Experience over 20 Years. World Neurosurg 119:e106–e117. https://doi.org/10.1016/j.wneu.2018.07.055

17. Ironside N, Chatain G, Asuzu D, et al (2018) Earlier post-operative hypocortisolemia may predict durable remission from Cushing’s disease. Eur J Endocrinol 178:255–263. https://doi.org/10.1530/EJE-17-0873

18. Mastorakos P, Taylor DG, Chen C-J, et al (2018) Prediction of cavernous sinus invasion in patients with Cushing’s disease by magnetic resonance imaging. J Neurosurg 1–6. https://doi.org/10.3171/2018.2.JNS172704

19. Mayberg M, Reintjes S, Patel A, et al (2018) Dynamics of postoperative serum cortisol after transsphenoidal surgery for Cushing’s disease: implications for immediate reoperation and remission. J Neurosurg 129:1268–1277. https://doi.org/10.3171/2017.6.JNS17635

20. Mortini P, Barzaghi LR, Albano L, et al (2018) Microsurgical therapy of pituitary adenomas. Endocrine 59:72–81. https://doi.org/10.1007/s12020-017-1458-3

21. Palejwala SK, Conger AR, Eisenberg AA, et al (2018) Pregnancy-associated Cushing’s disease? An exploratory retrospective study. Pituitary 21:584–592. https://doi.org/10.1007/s11102-018-0910-6

22. Qiao N, Swearingen B, Tritos NA (2018) Cushing’s disease in older patients: Presentation and outcome. Clin Endocrinol (Oxf) 89:444–453. https://doi.org/10.1111/cen.13799

23. Selek A, Cetinarslan B, Canturk Z, et al (2018) The Utility of Preoperative ACTH/Cortisol Ratio for the Diagnosis and Prognosis of Cushing’s Disease. J Neurosci Rural Pract 9:106–111. https://doi.org/10.4103/jnrp.jnrp_308_17

24. Bansal P, Lila A, Goroshi M, et al (2017) Duration of post-operative hypocortisolism predicts sustained remission after pituitary surgery for Cushing’s disease. Endocr Connect 6:625–636. https://doi.org/10.1530/EC-17-0175

25. Cebula H, Baussart B, Villa C, et al (2017) Efficacy of endoscopic endonasal transsphenoidal surgery for Cushing’s disease in 230 patients with positive and negative MRI. Acta Neurochir (Wien) 159:1227–1236. https://doi.org/10.1007/s00701-017-3140-1

26. Johnston PC, Kennedy L, Hamrahian AH, et al (2017) Surgical outcomes in patients with Cushing’s disease: the Cleveland clinic experience. Pituitary 20:430–440. https://doi.org/10.1007/s11102-017-0802-1

27. Keskin FE, Ozkaya HM, Bolayirli M, et al (2017) Outcomes of Primary Transsphenoidal Surgery in Cushing Disease: Experience of a Tertiary Center. World Neurosurg 106:374–381. https://doi.org/10.1016/j.wneu.2017.07.014

28. Powell MP, Narimova GJ, Halimova ZJ (2017) The Results of Surgical Treatment of Cushing Tumors in the Republic of Uzbekistan: Establishing Transsphenoidal Surgery in A Developing Nation. World Neurosurg 97:213–220. https://doi.org/10.1016/j.wneu.2016.09.067

29. Shin SS, Gardner PA, Ng J, et al (2017) Endoscopic Endonasal Approach for Adrenocorticotropic Hormone-Secreting Pituitary Adenomas: Outcomes and Analysis of Remission Rates and Tumor Biochemical Activity with Respect to Tumor Invasiveness. World Neurosurg 102:651-658.e1. https://doi.org/10.1016/j.wneu.2015.07.065

30. Chandler WF, Barkan AL, Hollon T, et al (2016) Outcome of Transsphenoidal Surgery for Cushing Disease: A Single-Center Experience Over 32 Years. Neurosurgery 78:216–223. https://doi.org/10.1227/NEU.0000000000001011

31. Chen Y, Tian S, Jian F, et al (2016) Clinical characteristics and surgical treatment of pituitary adrenocorticotropin-secreting macroadenomas: experience from a single-centre study. Int J Clin Exp Med 9:11147–11156

32. Machado MC, Alcantara AEE, Pereira ACL, et al (2016) Negative correlation between tumour size and cortisol/ACTH ratios in patients with Cushing’s disease harbouring microadenomas or macroadenomas. J Endocrinol Invest 39:1401–1409. https://doi.org/10.1007/s40618-016-0504-y

33. Sarkar S, Rajaratnam S, Chacko G, et al (2016) Pure endoscopic transsphenoidal surgery for functional pituitary adenomas: outcomes with Cushing’s disease. Acta Neurochir (Wien) 158:77–86; discussion 86. https://doi.org/10.1007/s00701-015-2638-7

34. Shirvani M, Motiei-Langroudi R, Sadeghian H (2016) Outcome of Microscopic Transsphenoidal Surgery in Cushing Disease: A Case Series of 96 Patients. World Neurosurg 87:170–175. https://doi.org/10.1016/j.wneu.2015.11.046

35. Solak M, Kraljevic I, Dusek T, et al (2016) Management of Cushing’s disease: a single-center experience. Endocrine 51:517–523. https://doi.org/10.1007/s12020-015-0695-6

36. Amlashi FG, Swearingen B, Faje AT, et al (2015) Accuracy of Late-Night Salivary Cortisol in Evaluating Postoperative Remission and Recurrence in Cushing’s Disease. J Clin Endocrinol Metab 100:3770–3777. https://doi.org/10.1210/jc.2015-2107

37. Kuo C-H, Yen Y-S, Wu J-C, et al (2015) Primary Endoscopic Transnasal Transsphenoidal Surgery for Magnetic Resonance Image–Positive Cushing Disease: Outcomes of a Series over 14 Years. World Neurosurg 84:772–779. https://doi.org/10.1016/j.wneu.2015.04.059

38. Yamada S, Inoshita N, Fukuhara N, et al (2015) Therapeutic outcomes in patients undergoing surgery after diagnosis of Cushing’s disease: A single-center study. Endocr J 62:1115–1125. https://doi.org/10.1507/endocrj.15-0463

39. Berker M, Işikay I, Berker D, et al (2013) Early promising results for the endoscopic surgical treatment of Cushing’s disease. Neurosurg Rev. https://doi.org/10.1007/s10143-013-0506-6

40. Dimopoulou C, Schopohl J, Rachinger W, et al (2014) Long-term remission and recurrence rates after first and second transsphenoidal surgery for Cushing’s disease: care reality in the Munich Metropolitan Region. Eur J Endocrinol 170:283–292. https://doi.org/10.1530/EJE-13-0634

41. Mehta GU, Bakhtian KD, Oldfield EH (2014) Effect of primary empty sella syndrome on pituitary surgery for Cushing’s disease. J Neurosurg 121:518–526. https://doi.org/10.3171/2014.3.JNS132012

42. Paluzzi A, Fernandez-Miranda JC, Tonya Stefko S, et al (2014) Endoscopic endonasal approach for pituitary adenomas: a series of 555 patients. Pituitary 17:307–319. https://doi.org/10.1007/s11102-013-0502-4

43. Storr HL, Drake WM, Evanson J, et al (2014) Endonasal endoscopic transsphenoidal pituitary surgery: early experience and outcome in paediatric Cushing’s disease. Clin Endocrinol (Oxf) 80:270–276. https://doi.org/10.1111/cen.12275

44. Valderrábano P, Aller J, García-Valdecasas L, et al (2014) Results of repeated transsphenoidal surgery in Cushing’s disease. Long-term follow-up. Endocrinol Nutr Organo Soc Espanola Endocrinol Nutr 61:176–183. https://doi.org/10.1016/j.endonu.2013.10.008

45. Alahmadi H, Cusimano MD, Woo K, et al (2013) Impact of technique on cushing disease outcome using strict remission criteria. Can J Neurol Sci J Can Sci Neurol 40:334–341. https://doi.org/10.1017/s031716710001427x

46. Alexandraki KI, Kaltsas GA, Isidori AM, et al (2013) Long-term remission and recurrence rates in Cushing’s disease: predictive factors in a single-centre study. Eur J Endocrinol 168:639–648. https://doi.org/10.1530/EJE-12-0921

47. Hameed N, Yedinak CG, Brzana J, et al (2013) Remission rate after transsphenoidal surgery in patients with pathologically confirmed Cushing’s disease, the role of cortisol, ACTH assessment and immediate reoperation: a large single center experience. Pituitary 16:452–458. https://doi.org/10.1007/s11102-012-0455-z

48. Lampropoulos KI, Samonis G, Nomikos P (2013) Factors influencing the outcome of microsurgical transsphenoidal surgery for pituitary adenomas: a study on 184 patients. Horm Athens Greece 12:254–264. https://doi.org/10.14310/horm.2002.1409

49. Lonser RR, Wind JJ, Nieman LK, et al (2013) Outcome of surgical treatment of 200 children with Cushing’s disease. J Clin Endocrinol Metab 98:892–901. https://doi.org/10.1210/jc.2012-3604

50. Starke RM, Reames DL, Chen C-J, et al (2013) Endoscopic transsphenoidal surgery for cushing disease: techniques, outcomes, and predictors of remission. Neurosurgery 72:240–247; discussion 247. https://doi.org/10.1227/NEU.0b013e31827b966a

51. Wagenmakers M a. EM, Boogaarts HD, Roerink SHPP, et al (2013) Endoscopic transsphenoidal pituitary surgery: a good and safe primary treatment option for Cushing’s disease, even in case of macroadenomas or invasive adenomas. Eur J Endocrinol 169:329–337. https://doi.org/10.1530/EJE-13-0325

52. Ciric I, Zhao J-C, Du H, et al (2012) Transsphenoidal surgery for Cushing disease: experience with 136 patients. Neurosurgery 70:70–80; discussion 80-81. https://doi.org/10.1227/NEU.0b013e31822dda2c

53. Honegger J, Schmalisch K, Beuschlein F, et al (2012) Contemporary microsurgical concept for the treatment of Cushing’s disease: endocrine outcome in 83 consecutive patients. Clin Endocrinol (Oxf) 76:560–567. https://doi.org/10.1111/j.1365-2265.2011.04268.x

54. Kim JH, Shin CS, Paek SH, et al (2012) Recurrence of Cushing’s disease after primary transsphenoidal surgery in a university hospital in Korea. Endocr J 59:881–888. https://doi.org/10.1507/endocrj.ej12-0109

55. Lonser RR, Ksendzovsky A, Wind JJ, et al (2012) Prospective evaluation of the characteristics and incidence of adenoma-associated dural invasion in Cushing disease. J Neurosurg 116:272–279. https://doi.org/10.3171/2011.8.JNS11456

56. Sun Y, Sun Q, Fan C, et al (2012) Diagnosis and therapy for Cushing’s disease with negative dynamic MRI finding: a single-centre experience. Clin Endocrinol (Oxf) 76:868–876. https://doi.org/10.1111/j.1365-2265.2011.04279.x

57. Fan C, Shi X, Sun Q, et al (2011) Bilateral inferior petrosal sinus sampling for the treatment of Cushing’s disease: Data from 52 cases from one institute over an eight-year period. Neural Regen Res 6:2203

58. Knappe UJ, Engelbach M, Konz K, et al (2011) Ultrasound-assisted microsurgery for Cushing’s disease. Exp Clin Endocrinol Diabetes Off J Ger Soc Endocrinol Ger Diabetes Assoc 119:191–200. https://doi.org/10.1055/s-0029-1241207

59. Storr HL, Alexandraki KI, Martin L, et al (2011) Comparisons in the epidemiology, diagnostic features and cure rate by transsphenoidal surgery between paediatric and adult-onset Cushing’s disease. Eur J Endocrinol 164:667–674. https://doi.org/10.1530/EJE-10-1120

60. Jagannathan J, Smith R, DeVroom HL, et al (2009) Outcome of using the histological pseudocapsule as a surgical capsule in Cushing disease. J Neurosurg 111:531–539. https://doi.org/10.3171/2008.8.JNS08339

61. Wagenmakers M a. EM, Netea‐Maier RT, Lindert EJV, et al (2009) Repeated transsphenoidal pituitary surgery (TS) via the endoscopic technique: a good therapeutic option for recurrent or persistent Cushing’s disease (CD). Clin Endocrinol (Oxf) 70:274–280. https://doi.org/10.1111/j.1365-2265.2008.03334.x

62. Atkinson JLD, Young WF, Meyer FB, et al (2008) Sublabial transseptal vs transnasal combined endoscopic microsurgery in patients with Cushing disease and MRI-depicted microadenomas. Mayo Clin Proc 83:550–553. https://doi.org/10.4065/83.5.550

63. Jehle S, Walsh JE, Freda PU, Post KD (2008) Selective use of bilateral inferior petrosal sinus sampling in patients with adrenocorticotropin-dependent Cushing’s syndrome prior to transsphenoidal surgery. J Clin Endocrinol Metab 93:4624–4632. https://doi.org/10.1210/jc.2008-0979

64. Prevedello DM, Pouratian N, Sherman J, et al (2008) Management of Cushing’s disease: outcome in patients with microadenoma detected on pituitary magnetic resonance imaging. J Neurosurg 109:751–759. https://doi.org/10.3171/JNS/2008/109/10/0751

65. Acebes JJ, Martino J, Masuet C, et al (2007) Early post-operative ACTH and cortisol as predictors of remission in Cushing’s disease. Acta Neurochir (Wien) 149:471–477; discussion 477-479. https://doi.org/10.1007/s00701-007-1133-1

66. Das NK, Lyngdoh BT, Bhakri BK, et al (2007) Surgical management of pediatric Cushing’s disease. Surg Neurol 67:251–257; discussion 257. https://doi.org/10.1016/j.surneu.2006.05.067

67. Dehdashti AR, Gentili F (2007) Current state of the art in the diagnosis and surgical treatment of Cushing disease: early experience with a purely endoscopic endonasal technique. Neurosurg Focus 23:1–8. https://doi.org/10.3171/foc.2007.23.3.11

68. Rollin G, Ferreira NP, Czepielewski MA (2007) Prospective evaluation of transsphenoidal pituitary surgery in 108 patients with Cushing’s disease. Arq Bras Endocrinol Metabol 51:1355–1361. https://doi.org/10.1590/s0004-27302007000800022

69. Testa RM, Albiger N, Occhi G, et al (2007) The usefulness of combined biochemical tests in the diagnosis of Cushing’s disease with negative pituitary magnetic resonance imaging. Eur J Endocrinol 156:241–248. https://doi.org/10.1530/eje.1.02332

70. Esposito F, Dusick JR, Cohan P, et al (2006) Clinical review: Early morning cortisol levels as a predictor of remission after transsphenoidal surgery for Cushing’s disease. J Clin Endocrinol Metab 91:7–13. https://doi.org/10.1210/jc.2005-1204

71. Hofmann BM, Hlavac M, Kreutzer J, et al (2006) Surgical treatment of recurrent Cushing’s disease. Neurosurgery 58:1108–1118; discussion 1108-1118. https://doi.org/10.1227/01.NEU.0000215945.26764.92

72. Batista D, Courkoutsakis NA, Oldfield EH, et al (2005) Detection of Adrenocorticotropin-Secreting Pituitary Adenomas by Magnetic Resonance Imaging in Children and Adolescents with Cushing Disease. J Clin Endocrinol Metab 90:5134–5140. https://doi.org/10.1210/jc.2004-1778

73. Joshi SM, Hewitt RJD, Storr HL, et al (2005) Cushing’s Disease in Children and Adolescents: 20 Years of Experience in a Single Neurosurgical Center. Neurosurgery 57:281–285. https://doi.org/10.1227/01.NEU.0000166580.94215.53

74. Kanter AS, Diallo AO, Jane JA, et al (2005) Single-center experience with pediatric Cushing’s disease. J Neurosurg 103:413–420. https://doi.org/10.3171/ped.2005.103.5.0413

75. Höybye C, Grenbäck E, Thorén M, et al (2004) Transsphenoidal surgery in Cushing disease: 10 years of experience in 34 consecutive cases. J Neurosurg 100:634–638. https://doi.org/10.3171/jns.2004.100.4.0634

76. Mehrazin M (2004) CUSHING’S DISEASE, TRANSSPHENOIDAL SURGICAL RESULTS OF 11 CASES. Arch Iran Med 7:53–56

77. Salenave S, Gatta B, Pecheur S, et al (2004) Pituitary magnetic resonance imaging findings do not influence surgical outcome in adrenocorticotropin-secreting microadenomas. J Clin Endocrinol Metab 89:3371–3376. https://doi.org/10.1210/jc.2003-031908

78. Cannavò S, Almoto B, Dall’Asta C, et al (2003) Long-term results of treatment in patients with ACTH-secreting pituitary macroadenomas. Eur J Endocrinol 149:195–200. https://doi.org/10.1530/eje.0.1490195

79. Zhao W, Zhan S, Cai Y, et al (2003) Surgical treatment and follow-up results of pituitary ACTH microadenoma: 18 years’ experience. Asian J Surg 26:22–25. https://doi.org/10.1016/S1015-9584(09)60210-4

80. Kristof RA, Schramm J, Redel L, et al (2002) Endocrinological outcome following first time transsphenoidal surgery for GH-, ACTH-, and PRL-secreting pituitary adenomas. Acta Neurochir (Wien) 144:555–561; discussion 561. https://doi.org/10.1007/s00701-002-0938-1

81. Rees DA, Hanna FWF, Davies JS, et al (2002) Long-term follow-up results of transsphenoidal surgery for Cushing’s disease in a single centre using strict criteria for remission. Clin Endocrinol (Oxf) 56:541–551. https://doi.org/10.1046/j.1365-2265.2002.01511.x

82. Shimon I, Ram Z, Cohen ZR, Hadani M (2002) Transsphenoidal surgery for Cushing’s disease: endocrinological follow-up monitoring of 82 patients. Neurosurgery 51:57–61; discussion 61-62. https://doi.org/10.1097/00006123-200207000-00008

83. Yap LB, Turner HE, Adams CBT, Wass J a. H (2002) Undetectable postoperative cortisol does not always predict long-term remission in Cushing’s disease: a single centre audit*. Clin Endocrinol (Oxf) 56:25–31. https://doi.org/10.1046/j.0300-0664.2001.01444.x

84. Ohnishi T, Arita N, Yoshimine T, Mori S (2000) Intracavernous sinus ectopic adrenocorticotropin-secreting tumours causing therapeutic failure in transsphenoidal surgery for Cushing’s disease. Acta Neurochir (Wien) 142:855–864. https://doi.org/10.1007/s007010070070

85. Semple PL, Vance ML, Findling J, Laws ER (2000) Transsphenoidal surgery for Cushing’s disease: outcome in patients with a normal magnetic resonance imaging scan. Neurosurgery 46:553–558; discussion 558-559. https://doi.org/10.1097/00006123-200003000-00005

86. Sheehan JM, Lopes MB, Sheehan JP, et al (2000) Results of transsphenoidal surgery for Cushing’s disease in patients with no histologically confirmed tumor. Neurosurgery 47:33–36; discussion 37-39. https://doi.org/10.1097/00006123-200007000-00008

87. Vallette-Kasic S, Dufour H, Mugnier M, et al (2000) Markers of tumor invasion are major predictive factors for the long-term outcome of corticotroph microadenomas treated by transsphenoidal adenomectomy. Eur J Endocrinol 143:761–768. https://doi.org/10.1530/eje.0.1430761

88. Flitsch J, Knappe UJ, Lüdecke DK (1999) Direct intraoperative micromethod for hormone measurements of pituitary tissue in Cushing’s disease. Surg Neurol 52:585–590; discussion 590-591. https://doi.org/10.1016/s0090-3019(99)00119-6

89. Gsponer J, De Tribolet N, Déruaz JP, et al (1999) Diagnosis, treatment, and outcome of pituitary tumors and other abnormal intrasellar masses. Retrospective analysis of 353 patients. Medicine (Baltimore) 78:236–269. https://doi.org/10.1097/00005792-199907000-00004

90. Devoe DJ, Miller WL, Conte FA, et al (1997) Long-term outcome in children and adolescents after transsphenoidal surgery for Cushing’s disease. J Clin Endocrinol Metab 82:3196–3202. https://doi.org/10.1210/jcem.82.10.4290

91. Knappe UJ, Lüdecke DK (1996) Transnasal microsurgery in children and adolescents with Cushing’s disease. Neurosurgery 39:484–492; discussion 492-493. https://doi.org/10.1097/00006123-199609000-00010

92. Oliverio PJ, Monsein LH, Wand GS, Debrun GM (1996) Bilateral simultaneous cavernous sinus sampling using corticotropin-releasing hormone in the evaluation of Cushing disease. Am J Neuroradiol 17:1669–1674

93. Weber A, Trainer PJ, Grossman AB, et al (1995) Investigation, management and therapeutic outcome in 12 cases of childhood and adolescent Cushing’s syndrome. Clin Endocrinol (Oxf) 43:19–28. https://doi.org/10.1111/j.1365-2265.1995.tb01888.x

94. Dyer EH, Civit T, Visot A, et al (1994) Transsphenoidal surgery for pituitary adenomas in children. Neurosurgery 34:207–212; discussion 212. https://doi.org/10.1227/00006123-199402000-00001

95. Magiakou MA, Mastorakos G, Oldfield EH, et al (1994) Cushing’s syndrome in children and adolescents. Presentation, diagnosis, and therapy. N Engl J Med 331:629–636. https://doi.org/10.1056/NEJM199409083311002

96. Robert F, Hardy J (1991) Cushing’s Disease: A Correlation of Radiological, Surgical and Pathological Findings with Therapeutic Results. Pathol - Res Pract 187:617–621. https://doi.org/10.1016/S0344-0338(11)80157-3

97. Friedman RB, Oldfield EH, Nieman LK, et al (1989) Repeat transsphenoidal surgery for Cushing’s disease. J Neurosurg 71:520–527. https://doi.org/10.3171/jns.1989.71.4.0520

98. Mampalam TJ, Tyrrell JB, Wilson CB (1988) Transsphenoidal microsurgery for Cushing disease. A report of 216 cases. Ann Intern Med 109:487–493. https://doi.org/10.7326/0003-4819-109-6-487

99. Styne DM, Grumbach MM, Kaplan SL, et al (1984) Treatment of Cushing’s disease in childhood and adolescence by transsphenoidal microadenomectomy. N Engl J Med 310:889–893. https://doi.org/10.1056/NEJM198404053101405

100. Wajchenberg BL, Silveira AA, Goldman J, et al (1979) Evaluation of resection of pituitary microadenoma for the treatment of Cushing’s disease in patients with radiologically normal sella turcica. Clin Endocrinol (Oxf) 11:323–331. https://doi.org/10.1111/j.1365-2265.1979.tb03081.x

101. Dai C, Feng M, Sun B, et al (2022) Surgical outcome of transsphenoidal surgery in Cushing’s disease: a case series of 1106 patients from a single center over 30 years. Endocrine 75:219–227. https://doi.org/10.1007/s12020-021-02848-1

102. Scherer M, Zerweck P, Becker D, et al (2022) The value of intraoperative MRI for resection of functional pituitary adenomas—a critical assessment of a consecutive single-center series of 114 cases. Neurosurg Rev 45:2895–2907. https://doi.org/10.1007/s10143-022-01810-7

103. Wu Y, Cai Y, Rui W, et al (2022) Contrast-enhanced 3D-T2-weighted SPACE sequence for MRI detection and localization of adrenocorticotropin (ACTH)-secreting pituitary microadenomas. Clin Endocrinol (Oxf) 96:578–588. https://doi.org/10.1111/cen.14574

104. Bestepe N, Ozdemir D, Polat B, et al (2021) The role of bilateral inferior petrosal sinus sampling in determining the preoperative localization of ACTH-secreting pituitary microadenomas in Cushing’s disease: Experience of a tertiary center. Clin Neurol Neurosurg 207:106724. https://doi.org/10.1016/j.clineuro.2021.106724

105. Tatsi C, Bompou ME, Flippo C, et al (2021) Paediatric patients with Cushing disease and negative pituitary MRI have a higher risk of nonremission after transsphenoidal surgery. Clin Endocrinol (Oxf) 95:856–862. https://doi.org/10.1111/cen.14560

106. Juthani RG, Reiner AS, Patel AR, et al (2020) Radiographic and clinical outcomes using intraoperative magnetic resonance imaging for transsphenoidal resection of pituitary adenomas. J Neurosurg 1–12. https://doi.org/10.3171/2020.4.JNS20178

107. Walia R, Gupta R, Bhansali A, et al (2020) Molecular Imaging Targeting Corticotropin-releasing Hormone Receptor for Corticotropinoma: A Changing Paradigm. J Clin Endocrinol Metab. https://doi.org/10.1210/clinem/dgaa755

108. Zhu J, Lu L, Yao Y, et al (2020) Long-term follow-up for ectopic ACTH-secreting pituitary adenoma in a single tertiary medical center and a literature review. Pituitary 23:149–159. https://doi.org/10.1007/s11102-019-01017-y

109. Cristante J, Lefournier V, Sturm N, et al (2019) Why we should still treat by neurosurgery patients with Cushing’s disease and a normal or inconclusive pituitary MRI. J Clin Endocrinol Metab. https://doi.org/10.1210/jc.2019-00333

110. Gazioglu N, Canaz H, Camlar M, et al (2019) Neurosurgical treatment of Cushing disease in pediatric patients: case series and review of literature. Childs Nerv Syst ChNS Off J Int Soc Pediatr Neurosurg 35:2127–2132. https://doi.org/10.1007/s00381-018-4013-5

111. Chatain GP, Patronas N, Smirniotopoulos JG, et al (2018) Potential utility of FLAIR in MRI-negative Cushing’s disease. J Neurosurg 129:620–628. https://doi.org/10.3171/2017.4.JNS17234

112. Cohen-Cohen S, Gardner PA, Alves-Belo JT, et al (2018) The medial wall of the cavernous sinus. Part 2: Selective medial wall resection in 50 pituitary adenoma patients. J Neurosurg 131:131–140. https://doi.org/10.3171/2018.5.JNS18595

113. De Sousa SMC, McCormack AI, McGrath S, Torpy DJ (2017) Prolactin correction for adequacy of petrosal sinus cannulation may diminish diagnostic accuracy in Cushing’s disease. Clin Endocrinol (Oxf) 87:515–522. https://doi.org/10.1111/cen.13401

114. Erfe JM, Perry A, McClaskey J, et al (2018) Long-term outcomes of tissue-based ACTH-antibody assay-guided transsphenoidal resection of pituitary adenomas in Cushing disease. J Neurosurg 129:629–641. https://doi.org/10.3171/2017.3.JNS162245

115. Lang M, Habboub G, Moon D, et al (2018) Comparison of Constructive Interference in Steady-State and T1-Weighted MRI Sequence at Detecting Pituitary Adenomas in Cushing’s Disease Patients. J Neurol Surg Part B Skull Base 79:593–598. https://doi.org/10.1055/s-0038-1642032

116. Lee JYK, Cho SS, Zeh R, et al (2018) Folate receptor overexpression can be visualized in real time during pituitary adenoma endoscopic transsphenoidal surgery with near-infrared imaging. J Neurosurg 129:390–403. https://doi.org/10.3171/2017.2.JNS163191

117. Valassi E, Franz H, Brue T, et al (2018) Preoperative medical treatment in Cushing’s syndrome: frequency of use and its impact on postoperative assessment: data from ERCUSYN. Eur J Endocrinol 178:399–409. https://doi.org/10.1530/EJE-17-0997

118. Zaidi HA, Reyes KDL, Barkhoudarian G, et al (2016) The utility of high-resolution intraoperative MRI in endoscopic transsphenoidal surgery for pituitary macroadenomas: early experience in the Advanced Multimodality Image Guided Operating suite. Neurosurg Focus 40:E18. https://doi.org/10.3171/2016.1.FOCUS15515

119. Masopust V, Netuka D, Beneš V, et al (2017) Magnetic resonance imaging and histology correlation in Cushing’s disease. Neurol Neurochir Pol 51:45–52. https://doi.org/10.1016/j.pjnns.2016.10.005

120. Witek P, Zieliński G, Szamotulska K, et al (2016) Clinicopathological predictive factors in the early remission of corticotroph pituitary macroadenomas in a tertiary referral centre. Eur J Endocrinol 174:539–549. https://doi.org/10.1530/EJE-15-1226

121. Afshari A, Ardeshirpour Y, Lodish MB, et al (2015) Facial Plethora: Modern Technology for Quantifying an Ancient Clinical Sign and Its Use in Cushing Syndrome. J Clin Endocrinol Metab 100:3928–3933. https://doi.org/10.1210/jc.2015-2497

122. Chittiboina P, Montgomery BK, Millo C, et al (2015) High-resolution18F-fluorodeoxyglucose positron emission tomography and magnetic resonance imaging for pituitary adenoma detection in Cushing disease. J Neurosurg 122:791–797. https://doi.org/10.3171/2014.10.JNS14911

123. Sun H, Yedinak C, Ozpinar A, et al (2015) Preoperative Lateralization Modalities for Cushing Disease: Is Dynamic Magnetic Resonance Imaging or Cavernous Sinus Sampling More Predictive of Intraoperative Findings? J Neurol Surg Part B Skull Base 76:218–224. https://doi.org/10.1055/s-0034-1543970

124. Potts MB, Shah JK, Molinaro AM, et al (2014) Cavernous and inferior petrosal sinus sampling and dynamic magnetic resonance imaging in the preoperative evaluation of Cushing’s disease. J Neurooncol 116:593–600. https://doi.org/10.1007/s11060-013-1342-9

125. Burkhardt T, Schmidt NO, Vettorazzi E, et al (2013) DHEA(S)—a novel marker in Cushing’s disease. Acta Neurochir (Wien) 155:479–484. https://doi.org/10.1007/s00701-012-1596-6

126. Kasaliwal R, Sankhe SS, Lila AR, et al (2013) Volume interpolated 3D-spoiled gradient echo sequence is better than dynamic contrast spin echo sequence for MRI detection of corticotropin secreting pituitary microadenomas. Clin Endocrinol (Oxf) 78:825–830. https://doi.org/10.1111/cen.12069

127. Tanei T, Nagatani T, Nakahara N, et al (2013) Use of High-Field Intraoperative Magnetic Resonance Imaging During Endoscopic Transsphenoidal Surgery for Functioning Pituitary Microadenomas and Small Adenomas Located in the Intrasellar Region. Neurol Med Chir (Tokyo) 53:501–510. https://doi.org/10.2176/nmc.53.501

128. Marić A, Kruljac I, Čerina V, et al (2012) Endocrinological outcomes of pure endoscopic transsphenoidal surgery: a Croatian Referral Pituitary Center experience. Croat Med J 53:224–233. https://doi.org/10.3325/cmj.2012.53.224

129. Sheth SA, Mian MK, Neal J, et al (2012) Transsphenoidal surgery for cushing disease after nondiagnostic inferior petrosal sinus sampling. Neurosurgery 71:14–22. https://doi.org/10.1227/NEU.0b013e31824f8e2e

130. Witek P, Zielinski G (2012) Predictive value of preoperative magnetic resonance imaging of the pituitary for surgical cure in Cushing’s disease. Turk Neurosurg 22:747–752. https://doi.org/10.5137/1019-5149.JTN.6199-12.2

131. Witek P, Zieliński G, Maksymowicz M, Zgliczyński W (2012) The relationship between efficacy of surgical treatment of Cushing disease and pathological – immunohistochemical and ultrastructural – confirmation of corticotroph tumour presence. Neurol Neurochir Pol 46:37–46. https://doi.org/10.5114/ninp.2012.27177

132. Hofstetter CP, Shin BJ, Mubita L, et al (2011) Endoscopic endonasal transsphenoidal surgery for functional pituitary adenomas. Neurosurg Focus 30:E10. https://doi.org/10.3171/2011.1.FOCUS10317

133. Lim JS, Lee SK, Kim SH, et al (2011) Intraoperative multiple-staged resection and tumor tissue identification using frozen sections provide the best result for the accurate localization and complete resection of tumors in Cushing’s disease. Endocrine 40:452–461. https://doi.org/10.1007/s12020-011-9499-5

134. Shah NS, George J, Acharya SV, et al (2011) Cushing Disease in Children and Adolescents: Twenty Years’ Experience in A Tertiary Care Center in India. Endocr Pract 17:369–376. https://doi.org/10.4158/EP10143.OR

135. Shi X, Sun Q, Bian L, et al (2011) Assessment of bilateral inferior petrosal sinus sampling in the diagnosis and surgical treatment of the ACTH-dependent Cushing’s syndrome: a comparison with other tests. Neuro Endocrinol Lett 32:865–873

136. Vitaz TW, Inkabi KE, Carrubba CJ (2011) Intraoperative MRI for transphenoidal procedures: Short-term outcome for 100 consecutive cases. Clin Neurol Neurosurg 113:731–735. https://doi.org/10.1016/j.clineuro.2011.07.025

137. Dias RP, Kumaran A, Chan LF, et al (2010) Diagnosis, management and therapeutic outcome in prepubertal Cushing’s disease. Eur J Endocrinol 162:603–609. https://doi.org/10.1530/EJE-09-0509

138. Oliveira RS de, Castro M de, Antonini SRR, et al (2010) Surgical management of pediatric Cushing’s disease: an analysis of 15 consecutive cases at a specialized neurosurgical center. Arq Bras Endocrinol Metabol 54:17–23. https://doi.org/10.1590/s0004-27302010000100004

139. Alzahrani AS, Farhat R, Al-Arifi A, et al (2009) The diagnostic value of fused positron emission tomography/computed tomography in the localization of adrenocorticotropin-secreting pituitary adenoma in Cushing’s disease. Pituitary 12:309–314. https://doi.org/10.1007/s11102-009-0180-4

140. Choe J-H, Lee K-S, Jeun S-S, et al (2008) Endocrine outcome of endoscopic endonasal transsphenoidal surgery in functioning pituitary adenomas. J Korean Neurosurg Soc 44:151–155. https://doi.org/10.3340/jkns.2008.44.3.151

141. Gazioglu N, Ulu MO, Ozlen F, et al (2008) Management of Cushing’s disease using cavernous sinus sampling: Effectiveness in tumor lateralization. Clin Neurol Neurosurg 110:333–338. https://doi.org/10.1016/j.clineuro.2007.11.008

142. Hofmann BM, Hlavac M, Martinez R, et al (2008) Long-term results after microsurgery for Cushing disease: experience with 426 primary operations over 35 years. J Neurosurg 108:9–18. https://doi.org/10.3171/JNS/2008/108/01/0009

143. Porterfield JR, Thompson GB, Young WF, et al (2008) Surgery for Cushing’s syndrome: an historical review and recent ten-year experience. World J Surg 32:659–677. https://doi.org/10.1007/s00268-007-9387-6

144. Zhang H-W, Sun W, Yang J, et al (2008) Diagnosis and treatment of pituitary microadenoma: report of 80 cases. Neurol Res 30:587–593. https://doi.org/10.1179/174313208X310287

145. Locatelli M, Vance ML, Laws ER (2005) CLINICAL REVIEW: The Strategy of Immediate Reoperation for Transsphenoidal Surgery for Cushing’s Disease. J Clin Endocrinol Metab 90:5478–5482. https://doi.org/10.1210/jc.2004-2436

146. Storr HL, Afshar F, Matson M, et al (2005) Factors influencing cure by transsphenoidal selective adenomectomy in paediatric Cushing’s disease. Eur J Endocrinol 152:825–833. https://doi.org/10.1530/eje.1.01921

147. Thomale U-W, Stover JF, Unterberg AW (2006) The use of neuronavigation in transnasal transsphenoidal pituitary surgery. Neurochirurgie 52:72. https://doi.org/10.1016/S0028-3770(06)71190-0

148. Woo YS, Isidori AM, Wat WZ, et al (2005) Clinical and biochemical characteristics of adrenocorticotropin-secreting macroadenomas. J Clin Endocrinol Metab 90:4963–4969. https://doi.org/10.1210/jc.2005-0070

149. Lienhardt A, Grossman AB, Dacie JE, et al (2001) Relative contributions of inferior petrosal sinus sampling and pituitary imaging in the investigation of children and adolescents with ACTH-dependent Cushing’s syndrome. J Clin Endocrinol Metab 86:5711–5714. https://doi.org/10.1210/jcem.86.12.8086

150. Graham KE, Samuels MH, Nesbit GM, et al (1999) Cavernous Sinus Sampling Is Highly Accurate in Distinguishing Cushing’s Disease from the Ectopic Adrenocorticotropin Syndrome and in Predicting Intrapituitary Tumor Location1. J Clin Endocrinol Metab 84:1602–1610. https://doi.org/10.1210/jcem.84.5.5654

151. Booth GL, Redelmeier DA, Grosman H, et al (1998) Improved diagnostic accuracy of inferior petrosal sinus sampling over imaging for localizing pituitary pathology in patients with Cushing’s disease. J Clin Endocrinol Metab 83:2291–2295. https://doi.org/10.1210/jcem.83.7.4956

152. Cukiert A, Andrioli M, Goldman J, et al (1998) Cavernous sinus invasion by pituitary macroadenomas. Neuroradiological, clinical and surgical correlation. Arq Neuropsiquiatr 56:107–110. https://doi.org/10.1590/s0004-282x1998000100017

153. Mason RB, Nieman LK, Doppman JL, Oldfield EH (1997) Selective excision of adenomas originating in or extending into the pituitary stalk with preservation of pituitary function. J Neurosurg 87:343–351. https://doi.org/10.3171/jns.1997.87.3.0343

154. Doppman JL, Ram Z, Shawker TH, Oldfield EH (1994) Intraoperative US of the pituitary gland. Work in progress. Radiology 192:111–115. https://doi.org/10.1148/radiology.192.1.8208921

155. Tripathi S, Ammini AC, Bhatia R, et al (1994) Cushing’s disease: pituitary imaging. Australas Radiol 38:183–186. https://doi.org/10.1111/j.1440-1673.1994.tb00170.x

156. Escourolle H, Abecassis JP, Bertagna X, et al (1993) Comparison of computerized tomography and magnetic resonance imaging for the examination of the pituitary gland in patients with Cushing’s disease. Clin Endocrinol (Oxf) 39:307–313. https://doi.org/10.1111/j.1365-2265.1993.tb02370.x

157. Buchfelder M, Nistor R, Fahlbusch R, Huk WJ (1993) The accuracy of CT and MR evaluation of the sella turcica for detection of adrenocorticotropic hormone-secreting adenomas in Cushing disease. AJNR Am J Neuroradiol 14:1183–1190

158. Boolell M, Gilford E, Arnott R, et al (1990) An overview of bilateral synchronous inferior petrosal sinus sampling (BSIPSS) in the pre-operative assessment of Cushing’s disease. Aust N Z J Med 20:765–770. https://doi.org/10.1111/j.1445-5994.1990.tb00420.x

159. Merola B, Lombardi G, Lupoli G, et al (1988) The use of intercavernous sinuses phlebography in the diagnosis of ACTH-secreting microadenomas. World J Surg 12:445–447. https://doi.org/10.1007/BF01655415

160. Tyrrell JB, Brooks RM, Fitzgerald PA, et al (1978) Cushing’s disease. Selective trans-sphenoidal resection of pituitary microadenomas. N Engl J Med 298:753–758. https://doi.org/10.1056/NEJM197804062981401

161. Mallari RJ, Thakur JD, Barkhoudarian G, et al (2022) Diagnostic Pitfalls in Cushing Disease: Surgical Remission Rates, Test Thresholds, and Lessons Learned in 105 Patients. J Clin Endocrinol Metab 107:205–218. https://doi.org/10.1210/clinem/dgab659

162. Pappy AL, Savinkina A, Bicknese C, et al (2019) Predictive modeling for pituitary adenomas: single center experience in 501 consecutive patients. Pituitary 22:520–531. https://doi.org/10.1007/s11102-019-00982-8

163. Crock PA, Lüdecke DK, Knappe UJ, Saeger W (2018) A personal series of 100 children operated for Cushing’s disease (CD): optimizing minimally invasive diagnosis and transnasal surgery to achieve nearly 100% remission including reoperations. J Pediatr Endocrinol Metab JPEM 31:1023–1031. https://doi.org/10.1515/jpem-2018-0262

164. Harker P, Feo-Lee O, Giraldo-Grueso M, Puentes JC (2017) Effectiveness of Bilateral Inferior Petrosal Sinuses Sampling in Tumor Lateralization: Intraoperative Findings and Postoperative Results. J Neurol Surg Part B Skull Base 78:506–511. https://doi.org/10.1055/s-0037-1604409

165. Berkmann S, Fandino J, Müller B, et al (2012) Pituitary surgery: experience from a large network in Central Switzerland. Swiss Med Wkly 142:w13680. https://doi.org/10.4414/smw.2012.13680

#### Supplementary Table 5. Quality Assessment

Q1. Was the study question or objective clearly stated?
Q2. Was the study population clearly and fully described, including a case definition?
Q3. Were the cases consecutive?
Q4. Were the subjects comparable?
Q5. Was the intervention clearly described?
Q6. Were the outcome measures clearly defined, valid, reliable, and implemented consistently across all study participants?
Q7. Was the length of follow-up adequate?
Q8. Were the statistical methods well-described?
Q9. Were the results well-described?

| Author | Year | Q1 | Q2 | Q3 | Q4 | Q5 | Q6 | Q7 | Q8 | Q9 |
| --- | --- | --- | --- | --- | --- | --- | --- | --- | --- | --- |
| Dai | 2022 | Yes | Yes | Yes | No | Yes | Yes | Not Reported | Not Applicable | Possibly |
| Mallari | 2022 | Yes | Yes | Yes | Yes | No | Yes | Yes | Not Reported | Not Applicable |
| Wu | 2022 | Yes | Yes | Possibly | Yes | No | Yes | Not Reported | Not Applicable | Possibly |
| Kang | 2022 | Yes | Yes | Possibly | Yes | No | Possibly | Yes | Not Applicable | Possibly |
| Scherer | 2022 | Yes | Possibly | Possibly | No | No | Possibly | No | Not Applicable | Possibly |
| Tatsi | 2021 | Yes | Yes | Yes | Yes | No | Yes | Not Reported | Not Applicable | Possibly |
| Bestepe | 2021 | Yes | Yes | Yes | Yes | Yes | Yes | Not Reported | Not Applicable | Possibly |
| Moszczyńska | 2021 | Yes | Yes | Yes | Yes | Possibly | Yes | No | Not Applicable | Yes |
| Berkmann | 2021 | Yes | Yes | Possibly | Possibly | No | Possibly | Yes | Not Applicable | Yes |
| Guaraldi | 2020 | Yes | Yes | Yes | Possibly | Yes | Yes | Yes | Yes | Yes |
| Strange | 2020 | Yes | Possibly | Possibly | Possibly | Not Applicable | Possibly | Yes | Yes | Yes |
| Serban | 2020 | Yes | Possibly | Yes | Yes | Yes | Yes | Yes | Yes | Yes |
| Bora | 2020 | Yes | Yes | Possibly | Not Reported | Yes | Yes | Possibly | Yes | Yes |
| Zhu J. | 2020 | Yes | Not Applicable | Yes | Possibly | Yes | Not Applicable | Possibly | Yes | Yes |
| Parksook W.W. | 2020 | Yes | Yes | Not Reported | Possibly | Yes | Yes | Yes | Yes | Yes |
| Juthani R.G. | 2020 | Yes | No | Yes | Not Reported | Yes | No | Not Reported | Yes | Possibly |
| Walia | 2020 | Yes | Possibly | Possibly | Not Reported | Yes | No | Not Reported | No | Yes |
| Chen | 2019 | Yes | Yes | Yes | Yes | Yes | Yes | Yes | Yes | Yes |
| Nadezhdina | 2019 | Yes | Yes | Yes | Yes | Yes | Yes | Yes | Yes | Yes |
| Pappy | 2019 | Yes | Possibly | Yes | Yes | Yes | Yes | Possibly | Yes | Yes |
| Todeschini | 2019 | Possibly | No | Yes | Possibly | Yes | Possibly | Yes | No | Possibly |
| Cristante | 2019 | Yes | Possibly | Yes | Yes | Possibly | Yes | No | Yes | Yes |
| Broersen | 2019 | Yes | Yes | Yes | Possibly | Yes | No | Yes | Yes | Yes |
| Gazioglu N. | 2019 | Possibly | Possibly | Yes | No | Yes | No | Yes | No | Possibly |
| Liu Y. | 2019 | Yes | Yes | Not Reported | Yes | Yes | Yes | Yes | Yes | Yes |
| Palejwala | 2018 | Yes | Yes | Yes | Possibly | Yes | Yes | Yes | Yes | Yes |
| Cohen-Cohen | 2018 | Yes | Yes | Possibly | Yes | Yes | Yes | Yes | No | Yes |
| Crock | 2018 | No | Yes | Possibly | Possibly | Yes | Yes | Not Reported | Not Reported | Possibly |
| Brichard | 2018 | Yes | Possibly | Possibly | Yes | Yes | Yes | Yes | Yes | Yes |
| Qiao | 2018 | Yes | Yes | Yes | Yes | Possibly | Yes | Yes | Yes | Yes |
| Zaidi | 2018 | Yes | Yes | Yes | Possibly | Yes | No | Yes | Possibly | Possibly |
| Mayberg | 2018 | Yes | Yes | Yes | Yes | Yes | Yes | Yes | Yes | Yes |
| Chatain | 2018 | Yes | Yes | Yes | Yes | Yes | Yes | Not Reported | Yes | Yes |
| Erfe | 2018 | Yes | Yes | Possibly | Yes | Yes | Yes | Yes | Yes | Yes |
| Valassi E. | 2018 | Yes | Possibly | Not Reported | Possibly | Yes | Possibly | Possibly | Yes | Yes |
| Ironside N. | 2018 | Possibly | Yes | Yes | Yes | Yes | Yes | Possibly | Yes | Yes |
| Mortini P. | 2018 | Possibly | Possibly | Yes | Possibly | No | Yes | Yes | Yes | Yes |
| Selek A. | 2018 | Yes | Yes | Not Reported | Yes | Possibly | Yes | Yes | Yes | Yes |
| De Sousa S.M.C. | 2018 | Yes | Possibly | Yes | Possibly | Possibly | No | Not Reported | Not Applicable | Yes |
| Mastorakos P. | 2018 | Yes | Possibly | Not Reported | Yes | Yes | Yes | Not Reported | Yes | Yes |
| Lee J.Y.K | 2018 | Yes | Yes | Not Reported | Possibly | Yes | Yes | No | Possibly | Yes |
| Lang M. | 2018 | Yes | Yes | Not Reported | Possibly | Possibly | Yes | Not Applicable | Not Applicable | Yes |
| Harker | 2017 | Yes | Yes | Yes | Yes | Yes | Yes | Possibly | Yes | Yes |
| Bansal | 2017 | Possibly | Yes | Yes | Yes | Yes | Yes | Possibly | Yes | Yes |
| Keskin | 2017 | Possibly | Yes | Yes | Possibly | Yes | Yes | Yes | Yes | Yes |
| Cebula | 2017 | Yes | Yes | Yes | Yes | Yes | Yes | Yes | Yes | Yes |
| Johnston | 2017 | Yes | Yes | Yes | Possibly | Yes | Yes | Yes | Yes | Yes |
| Masopust | 2017 | Yes | Possibly | Possibly | Possibly | Yes | Possibly | Possibly | No | Possibly |
| Powell | 2017 | Possibly | Possibly | Possibly | Possibly | Yes | Yes | Possibly | Yes | Yes |
| Shin | 2017 | Yes | Yes | Yes | Yes | Yes | Yes | Yes | Yes | Yes |
| Machado | 2016 | Yes | Yes | Possibly | Possibly | No | Yes | Yes | Yes | Yes |
| Witek | 2016 | Yes | Yes | Yes | Yes | Yes | Yes | No | Yes | Yes |
| Shirvani | 2016 | Yes | Yes | Yes | Possibly | Yes | Yes | Yes | No | Yes |
| Sarkar | 2016 | Possibly | Yes | Yes | Possibly | Yes | Yes | Yes | Yes | Yes |
| Chandler | 2016 | Yes | Possibly | Yes | Yes | Yes | Yes | Yes | Yes | Yes |
| Solak | 2016 | Yes | Yes | Yes | Yes | Yes | Yes | Yes | Yes | Yes |
| Chen Y. | 2016 | Yes | Yes | Yes | Yes | Possibly | Yes | Not Reported | Yes | Possibly |
| Yamada | 2015 | Yes | Yes | Possibly | Possibly | Yes | Yes | Yes | Yes | Yes |
| Afshari | 2015 | Yes | No | Yes | Possibly | Yes | Yes | Not Reported | Yes | Yes |
| Sun | 2015 | Yes | Possibly | Yes | Yes | Yes | Yes | Possibly | Yes | Yes |
| Amlashi | 2015 | Yes | Yes | Yes | Yes | Yes | Yes | Yes | Yes | Yes |
| Kuo | 2015 | Yes | Yes | Yes | Yes | Yes | Yes | Yes | Yes | Yes |
| Chittiboina P | 2015 | Yes | Yes | Yes | Possibly | Yes | Possibly | Not Reported | Yes | Yes |
| Mehta | 2014 | Yes | Yes | Yes | Yes | Yes | Yes | Yes | Yes | Yes |
| Dimopoulou | 2014 | Yes | Yes | Yes | Yes | Yes | Yes | Yes | Yes | Yes |
| Storr | 2014 | Yes | Yes | Possibly | Yes | Yes | Yes | Yes | Not Applicable | Yes |
| Potts M.B. | 2014 | Yes | Possibly | Yes | Yes | Yes | Possibly | Possibly | Possibly | Yes |
| Paluzzi A. | 2014 | Yes | Yes | Yes | Possibly | Yes | Possibly | Yes | Not Reported | Yes |
| Berker M. | 2014 | Yes | Yes | Yes | Possibly | Yes | Yes | Yes | No | Yes |
| Tanei | 2013 | Yes | Yes | Yes | Yes | Yes | Yes | Yes | Yes | Yes |
| Wagenmakers | 2013 | Possibly | Yes | Yes | Possibly | Yes | Yes | Yes | Yes | Yes |
| Alahmadi | 2013 | Yes | Yes | Yes | Possibly | Yes | Yes | Yes | Yes | Yes |
| Lonser | 2013 | Possibly | Yes | Yes | Yes | Yes | Yes | Yes | Yes | Yes |
| Alexandraki | 2013 | Yes | Possibly | Possibly | Yes | Yes | Possibly | Yes | Yes | Yes |
| Burkhardt | 2013 | Yes | Possibly | Yes | Possibly | Yes | Yes | Not Reported | Yes | Yes |
| Hameed | 2013 | Yes | Possibly | Yes | Possibly | No | Possibly | Yes | Yes | Yes |
| Starke | 2013 | Yes | Possibly | Possibly | No | Yes | Yes | Yes | Yes | Yes |
| Kasaliwal | 2013 | Yes | Possibly | Yes | Yes | Yes | Yes | Not Reported | Not Reported | Yes |
| Lampropoulos | 2013 | Yes | No | Yes | Possibly | Yes | Yes | Yes | Yes | Yes |
| Witek | 2012 | Yes | Possibly | Yes | Possibly | Yes | Not Reported | Yes | Yes | Yes |
| Berkmann | 2012 | No | Possibly | Possibly | No | Yes | No | Yes | Possibly | Possibly |
| Kim | 2012 | Yes | Possibly | Yes | Yes | Possibly | Possibly | Yes | Yes | Yes |
| Witek | 2012 | Yes | Possibly | Yes | Possibly | Possibly | Yes | Yes | Yes | Yes |
| Sheth | 2012 | Yes | Possibly | Yes | Possibly | No | Yes | Yes | Yes | Possibly |
| Sun | 2012 | Possibly | Yes | Yes | Yes | Yes | Yes | Yes | Yes | Yes |
| Honegger | 2012 | Possibly | Possibly | Yes | Yes | Yes | Yes | Yes | No | Possibly |
| Lonser | 2012 | Possibly | No | Yes | No | Possibly | No | Yes | No | Yes |
| Ciric | 2012 | Yes | No | Possibly | Possibly | Possibly | Yes | Yes | Yes | Yes |
| Shi | 2011 | Yes | Yes | Yes | No | No | Yes | Yes | Yes | Yes |
| Vitaz | 2011 | Yes | No | Yes | Not Reported | Possibly | No | Not Reported | Not Reported | Possibly |
| Lim | 2011 | Yes | No | Not Reported | No | Yes | Yes | Yes | Not Applicable | Yes |
| Hofstetter | 2011 | Yes | Possibly | Yes | Possibly | Possibly | Yes | Yes | Yes | Yes |
| Storr | 2011 | Yes | Possibly | Possibly | No | Possibly | Yes | Not Reported | Yes | Yes |
| Shah | 2011 | Yes | Possibly | Possibly | Possibly | No | Yes | Yes | Not Applicable | Yes |
| Knappe | 2011 | Yes | No | Yes | Possibly | Possibly | Possibly | Possibly | Not Applicable | Yes |
| Fan C. | 2011 | Yes | Yes | Not Reported | Yes | Possibly | Possibly | Yes | No | Yes |
| Sheth S. | 2011 | Possibly | Possibly | Not Reported | Possibly | Yes | Yes | Yes | Yes | Yes |
| Oliveira | 2010 | Yes | No | Yes | Possibly | Possibly | Yes | Yes | Not Applicable | Yes |
| Dias | 2010 | Possibly | Possibly | Possibly | Possibly | Yes | Yes | Yes | Not Applicable | Yes |
| Jagannathan | 2009 | Yes | Yes | Possibly | Possibly | Possibly | Yes | Yes | Not Applicable | Yes |
| Wagenmakers | 2009 | Yes | No | Yes | Possibly | Yes | Yes | Yes | Yes | Yes |
| Alzahrani A.S. | 2009 | Yes | Yes | Possibly | Possibly | Yes | Possibly | Not Reported | Possibly | Yes |
| Choe | 2008 | Possibly | Yes | Not Reported | Not Reported | Possibly | Yes | Not Reported | Not Applicable | Yes |
| Prevedello | 2008 | Yes | Yes | Possibly | Yes | Possibly | Yes | Yes | Yes | Yes |
| Jehle | 2008 | Yes | Yes | Yes | Yes | Yes | Yes | Yes | Yes | Yes |
| Zhang | 2008 | Yes | No | Possibly | Not Reported | Possibly | Yes | Not Reported | Not Applicable | Yes |
| Atkinson | 2008 | Yes | No | Yes | Yes | No | Possibly | No | Yes | Yes |
| Gazioglu | 2008 | Yes | Yes | Yes | Possibly | Yes | Yes | Not Reported | Not Applicable | Yes |
| Porterfield | 2008 | Possibly | No | Yes | Not Reported | No | No | Not Reported | Not Applicable | Yes |
| Hofmann | 2008 | Yes | Possibly | Possibly | Yes | Yes | Yes | Yes | Not Applicable | Yes |
| Rollin | 2007 | Yes | Possibly | Possibly | Yes | Possibly | Yes | Yes | Yes | Yes |
| Dehdashti | 2007 | Yes | Possibly | Yes | Possibly | Yes | Yes | Yes | Not Applicable | Yes |
| Acebes | 2007 | Yes | Yes | Yes | Yes | Yes | Possibly | Yes | Yes | Yes |
| Das | 2007 | Yes | Possibly | Yes | Possibly | Possibly | Yes | Yes | Not Applicable | Yes |
| Testa | 2007 | Yes | Possibly | Yes | Possibly | Yes | Yes | Yes | Yes | Yes |
| Hofmann | 2006 | Yes | Possibly | Yes | Yes | Yes | Possibly | Yes | Possibly | Yes |
| Esposito | 2006 | Yes | Possibly | Yes | Yes | Yes | Yes | Yes | Yes | Yes |
| Kanter | 2005 | Yes | Yes | Yes | Possibly | Yes | Yes | Yes | Possibly | Yes |
| Joshi | 2005 | Yes | No | Yes | Possibly | Possibly | Yes | Possibly | Not Applicable | Yes |
| Locatelli | 2005 | Yes | Possibly | Possibly | Possibly | Possibly | Yes | Possibly | Not Applicable | Possibly |
| Storr | 2005 | Yes | Possibly | Possibly | Possibly | Possibly | Yes | Possibly | Yes | Yes |
| Batista | 2005 | Yes | No | Yes | Possibly | Possibly | Possibly | Not Reported | Yes | Yes |
| Woo | 2005 | Yes | Possibly | Possibly | Possibly | No | Yes | Not Reported | Yes | Yes |
| Thomale UW | 2005 | Yes | Yes | Possibly | Possibly | Possibly | Possibly | No | Not Reported | Possibly |
| Salenave | 2004 | Yes | Possibly | Yes | Possibly | Possibly | Yes | Possibly | Yes | Yes |
| Mehrazin M. | 2004 | Yes | Yes | Yes | Possibly | Yes | No | Yes | Not Reported | No |
| Zhao | 2003 | Possibly | Possibly | Possibly | No | Possibly | Possibly | Yes | Not Applicable | Yes |
| Shimon | 2002 | Yes | No | Possibly | Possibly | Possibly | Yes | Possibly | Not Applicable | Yes |
| Kristof | 2002 | Yes | Yes | Yes | Possibly | No | Yes | Possibly | Yes | Yes |
| Rees | 2002 | Possibly | Yes | Yes | No | Yes | Yes | Possibly | Yes | Yes |
| Yap | 2002 | Yes | No | No | No | No | Yes | Possibly | Yes | Yes |
| Lienhardt | 2001 | Possibly | Possibly | Possibly | No | Possibly | Yes | Possibly | Not Applicable | Yes |
| Vallette-Kasic | 2000 | Yes | Possibly | Yes | No | Yes | Yes | Yes | Yes | Yes |
| Ohnishi | 2000 | Possibly | Possibly | Yes | No | Yes | No | Possibly | Not Applicable | Yes |
| Sheehan | 2000 | Possibly | No | Possibly | Possibly | No | Possibly | Possibly | Yes | Yes |
| Semple | 2000 | Yes | Possibly | Possibly | Yes | Possibly | Yes | Possibly | Not Applicable | Yes |
| Gsponer | 1999 | Yes | Possibly | Yes | Possibly | Possibly | No | Not Reported | No | Yes |
| Graham | 1999 | Yes | Possibly | Yes | Yes | Yes | Possibly | Yes | Yes | Yes |
| Flitsch J | 1999 | Yes | Yes | Not Reported | Possibly | Yes | Possibly | No | Yes | Yes |
| Booth | 1998 | Yes | Yes | Yes | Yes | Yes | Possibly | Not Reported | Possibly | Yes |
| Cukiert A | 1998 | Possibly | No | Not Reported | No | Possibly | No | No | Not Reported | No |
| Devoe | 1997 | Possibly | Yes | Yes | Yes | Yes | Possibly | Yes | Not Reported | Yes |
| Mason | 1997 | Possibly | Possibly | Yes | Possibly | Possibly | Yes | No | Not Applicable | Yes |
| Oliverio | 1996 | Yes | Yes | Yes | Yes | Yes | Possibly | Not Reported | Not Reported | Yes |
| Knappe | 1996 | Possibly | Yes | Not Reported | Yes | Possibly | Possibly | Yes | No | Yes |
| Weber | 1995 | Possibly | Yes | Not Reported | Possibly | Possibly | Yes | Yes | No | Yes |
| Magiakou | 1994 | Possibly | Yes | Not Reported | Possibly | Not Applicable | Yes | Yes | No | Yes |
| Tripathi | 1994 | Yes | Yes | Not Reported | Possibly | Possibly | Possibly | Not Reported | Not Applicable | Yes |
| Dyer | 1994 | No | No | Not Reported | Possibly | Possibly | No | Yes | Not Reported | Possibly |
| Doppman J.L. | 1994 | Yes | Possibly | Not Reported | Possibly | Yes | Possibly | Not Reported | Not Reported | Yes |
| Doppman J.L. | 1994 | Possibly | Possibly | Yes | Not Reported | Yes | No | Possibly | Not Applicable | No |
| Escourolle | 1993 | Yes | Possibly | Not Reported | Possibly | Yes | No | Not Reported | Yes | Yes |
| Buchfelder | 1992 | Yes | No | Yes | Not Reported | Yes | Yes | Not Reported | No | Yes |
| Robert | 1991 | No | No | Yes | Possibly | Possibly | No | Yes | Not Reported | Possibly |
| Boolell | 1990 | Yes | No | Not Reported | Possibly | No | Not Reported | Not Reported | Not Applicable | Possibly |
| Friedman | 1989 | Yes | Possibly | Possibly | Possibly | No | Yes | No | Not Applicable | Yes |
| Mampalam | 1988 | Possibly | Possibly | Possibly | No | Yes | Possibly | Yes | Not Applicable | Yes |
| Merola | 1988 | Yes | Possibly |  | Not Reported | Possibly | No | Not Reported | Not Applicable | No |
| Wajchenberg | 1979 | No | Yes | Not Reported | Yes | Possibly | Possibly | Possibly | No | Yes |

#### Supplementary Table 6. All remission models for Remission

|  | Model | BIC | AIC | AICc | ll | dev |
| --- | --- | --- | --- | --- | --- | --- |
| 1 | PrevTSS+Macro+Undetect+CSI+log(RemCortisolThreshold)+Octreotide | 675.45 | 643.8 | 644.5 | -312.9 | 625.8 |
| 2 | PrevTSS+Macro+Undetect+CSI+log(RemCortisolThreshold)+GRE | 675.86 | 644.2 | 644.9 | -313.1 | 626.2 |
| 3 | PrevTSS+Macro+Undetect+CSI+log(RemCortisolThreshold) | 676.37 | 648.2 | 648.8 | -316.1 | 632.2 |
| 4 | PrevTSS+Macro+Undetect+CSI+log(RemCortisolThreshold)+GRE+DMRI | 677.29 | 642.1 | 643.0 | -311.1 | 622.1 |
| 5 | PrevTSS+MaleProp+Macro+Undetect+CSI+log(RemCortisolThreshold)+Octreotide | 677.33 | 642.2 | 643.1 | -311.1 | 622.2 |
| 6 | PrevTSS+MaleProp+Macro+Undetect+CSI+log(RemCortisolThreshold)+GRE | 677.34 | 642.2 | 643.1 | -311.1 | 622.2 |
| 7 | PrevTSS+Macro+Undetect+CSI+log(RemCortisolThreshold)+USS+Octreotide | 677.35 | 642.2 | 643.1 | -311.1 | 622.2 |
| 8 | PrevTSS+MaleProp+Macro+Undetect+CSI+log(RemCortisolThreshold) | 677.88 | 646.2 | 646.9 | -314.1 | 628.2 |
| … | … | … | … | … | … | … |
| … | … | … | … | … | … | … |
| … | … | … | … | … | … | … |
| 26104 | Undetect+Age+CT+USS+iMRI+PET+PreCT+Octreotide+IntraopFluorescence | 764.42 | 722.3 | 723.6 | -349.2 | 698.3 |
| 26105 | Undetect+Age+CT+USS+iMRI+PET+PreCT+IntraopFluorescence | 764.74 | 726.1 | 727.2 | -352.0 | 704.1 |
| 26106 | MaleProp+Age+CT+USS+iMRI+PET+PreCT+IntraopFluorescence | 764.91 | 726.3 | 727.4 | -352.1 | 704.3 |
| 26107 | Age+CT+iMRI+PET+PreCT+IntraopFluorescence | 765.01 | 733.3 | 734.1 | -357.7 | 715.3 |
| 26108 | Age+CT+iMRI+PET+PreCT+Octreotide+IntraopFluorescence | 765.06 | 729.9 | 730.8 | -354.9 | 709.9 |
| 26109 | MaleProp+Age+CT+USS+iMRI+PET+PreCT+Octreotide+IntraopFluorescence | 765.31 | 723.2 | 724.5 | -349.6 | 699.2 |
| 26110 | Age+CT+USS+iMRI+PET+PreCT+IntraopFluorescence | 766.79 | 731.6 | 732.5 | -355.8 | 711.6 |
| 26111 | Age+CT+USS+iMRI+PET+PreCT+Octreotide+IntraopFluorescence | 766.83 | 728.2 | 729.3 | -353.1 | 706.2 |

#### Supplementary Table 7. Remission rates of the Octreotide best model

| term | estimate | std.error | statistic | df | p.value |
| --- | --- | --- | --- | --- | --- |
| intercept | 1.4041 | 0.1745 | 8.0473 | 74.2079 | 0.0000 |
| PrevTSS | -0.7418 | 0.2554 | -2.9041 | 23.6685 | 0.0079 |
| Macro | -0.4644 | 0.2822 | -1.6455 | 19.0284 | 0.1163 |
| Undetect | -0.6999 | 0.1573 | -4.4484 | 69.4374 | 0.0000 |
| CSI | -1.5615 | 0.5595 | -2.7907 | 22.1833 | 0.0106 |
| log(RemCortisolThreshold) | 0.3197 | 0.1138 | 2.8086 | 63.2497 | 0.0066 |
| Octreotide | -2.5843 | 1.3139 | -1.9669 | 224.9920 | 0.0504 |

#### Supplementary Table 8. Baseline models only for Remission

|  | Model | BIC | AIC | AICc | ll | dev |
| --- | --- | --- | --- | --- | --- | --- |
| 3 | PrevTSS+Macro+Undetect+CSI+log(RemCortisolThreshold) | 676.37 | 648.2 | 648.8 | -316.1 | 632.2 |
| 8 | PrevTSS+MaleProp+Macro+Undetect+CSI+log(RemCortisolThreshold) | 677.88 | 646.2 | 646.9 | -314.1 | 628.2 |
| 27 | PrevTSS+MaleProp+Macro+Undetect+CSI+log(RemCortisolThreshold)+Age | 679.61 | 644.4 | 645.4 | -312.2 | 624.4 |
| 30 | PrevTSS+Macro+Undetect+CSI+log(RemCortisolThreshold)+Age | 679.70 | 648.0 | 648.8 | -315.0 | 630.0 |
| 36 | PrevTSS+Macro+Undetect+CSI+RemCortisolThreshold | 679.99 | 651.8 | 652.4 | -317.9 | 635.8 |
| 43 | PrevTSS+Undetect+CSI+log(RemCortisolThreshold) | 680.40 | 655.7 | 656.2 | -320.8 | 641.7 |
| 52 | PrevTSS+MaleProp+Macro+Undetect+CSI+RemCortisolThreshold | 680.80 | 649.1 | 649.9 | -315.6 | 631.1 |
| 107 | PrevTSS+MaleProp+Undetect+CSI+log(RemCortisolThreshold) | 682.46 | 654.3 | 654.8 | -319.1 | 638.3 |
| … | … | … | … | … | … | … |
| … | … | … | … | … | … | … |
| … | … | … | … | … | … | … |
| 22386 | MaleProp+RemCortisolThreshold+Age | 743.63 | 722.4 | 722.8 | -355.2 | 710.4 |
| 22450 | MaleProp+Undetect | 743.89 | 726.2 | 726.4 | -358.1 | 716.2 |
| 22721 | RemCortisolThreshold+Age | 744.80 | 727.1 | 727.4 | -358.6 | 717.1 |
| 22823 | MaleProp | 745.16 | 731.0 | 731.2 | -361.5 | 723.0 |
| 22889 | MaleProp+Undetect+Age | 745.45 | 724.2 | 724.6 | -356.1 | 712.2 |
| 23229 | Undetect+Age | 746.87 | 729.2 | 729.4 | -359.6 | 719.2 |
| 23280 | MaleProp+Age | 747.05 | 729.4 | 729.6 | -359.7 | 719.4 |
| 23821 | Age | 748.84 | 734.7 | 734.8 | -363.3 | 726.7 |

#### Supplementary Table 9. Imaging Types for Remission

|  | Model | BIC | AIC | AICc | ll | dev |
| --- | --- | --- | --- | --- | --- | --- |
| 1 | PrevTSS+Macro+Undetect+CSI+log(RemCortisolThreshold)+Octreotide | 675.45 | 643.8 | 644.5 | -312.9 | 625.8 |
| 3 | PrevTSS+Macro+Undetect+CSI+log(RemCortisolThreshold) | 676.37 | 648.2 | 648.8 | -316.1 | 632.2 |
| 5 | PrevTSS+MaleProp+Macro+Undetect+CSI+log(RemCortisolThreshold)+Octreotide | 677.33 | 642.2 | 643.1 | -311.1 | 622.2 |
| 7 | PrevTSS+Macro+Undetect+CSI+log(RemCortisolThreshold)+USS+Octreotide | 677.35 | 642.2 | 643.1 | -311.1 | 622.2 |
| 9 | PrevTSS+Macro+Undetect+CSI+log(RemCortisolThreshold)+USS | 678.25 | 646.6 | 647.3 | -314.3 | 628.6 |
| 10 | PrevTSS+Macro+Undetect+CSI+log(RemCortisolThreshold)+PET+Octreotide | 678.46 | 643.3 | 644.2 | -311.6 | 623.3 |
| 11 | PrevTSS+Macro+Undetect+CSI+log(RemCortisolThreshold)+Octreotide+IntraopFluorescence | 678.55 | 643.4 | 644.3 | -311.7 | 623.4 |
| 12 | PrevTSS+Macro+Undetect+CSI+log(RemCortisolThreshold)+Age+Octreotide | 678.69 | 643.5 | 644.4 | -311.8 | 623.5 |
| … | … | … | … | … | … | … |
| … | … | … | … | … | … | … |
| … | … | … | … | … | … | … |
| 26104 | Undetect+Age+CT+USS+iMRI+PET+PreCT+Octreotide+IntraopFluorescence | 764.42 | 722.3 | 723.6 | -349.2 | 698.3 |
| 26105 | Undetect+Age+CT+USS+iMRI+PET+PreCT+IntraopFluorescence | 764.74 | 726.1 | 727.2 | -352.0 | 704.1 |
| 26106 | MaleProp+Age+CT+USS+iMRI+PET+PreCT+IntraopFluorescence | 764.91 | 726.3 | 727.4 | -352.1 | 704.3 |
| 26107 | Age+CT+iMRI+PET+PreCT+IntraopFluorescence | 765.01 | 733.3 | 734.1 | -357.7 | 715.3 |
| 26108 | Age+CT+iMRI+PET+PreCT+Octreotide+IntraopFluorescence | 765.06 | 729.9 | 730.8 | -354.9 | 709.9 |
| 26109 | MaleProp+Age+CT+USS+iMRI+PET+PreCT+Octreotide+IntraopFluorescence | 765.31 | 723.2 | 724.5 | -349.6 | 699.2 |
| 26110 | Age+CT+USS+iMRI+PET+PreCT+IntraopFluorescence | 766.79 | 731.6 | 732.5 | -355.8 | 711.6 |
| 26111 | Age+CT+USS+iMRI+PET+PreCT+Octreotide+IntraopFluorescence | 766.83 | 728.2 | 729.3 | -353.1 | 706.2 |

#### Supplementary Table 10. MRI Sequences for Remission

|  | Model | BIC | AIC | AICc | ll | dev |
| --- | --- | --- | --- | --- | --- | --- |
| 2 | PrevTSS+Macro+Undetect+CSI+log(RemCortisolThreshold)+GRE | 675.86 | 644.2 | 644.9 | -313.1 | 626.2 |
| 3 | PrevTSS+Macro+Undetect+CSI+log(RemCortisolThreshold) | 676.37 | 648.2 | 648.8 | -316.1 | 632.2 |
| 4 | PrevTSS+Macro+Undetect+CSI+log(RemCortisolThreshold)+GRE+DMRI | 677.29 | 642.1 | 643.0 | -311.1 | 622.1 |
| 6 | PrevTSS+MaleProp+Macro+Undetect+CSI+log(RemCortisolThreshold)+GRE | 677.34 | 642.2 | 643.1 | -311.1 | 622.2 |
| 14 | PrevTSS+MaleProp+Macro+Undetect+CSI+log(RemCortisolThreshold)+GRE+DMRI | 678.86 | 640.2 | 641.3 | -309.1 | 618.2 |
| 15 | PrevTSS+Macro+Undetect+CSI+RemCortisolThreshold+GRE | 678.95 | 647.3 | 648.0 | -314.6 | 629.3 |
| 19 | PrevTSS+Macro+Undetect+CSI+log(RemCortisolThreshold)+DMRI | 679.16 | 647.5 | 648.2 | -314.7 | 629.5 |
| 21 | PrevTSS+Macro+Undetect+CSI+log(RemCortisolThreshold)+Age+GRE | 679.30 | 644.1 | 645.1 | -312.1 | 624.1 |
| … | … | … | … | … | … | … |
| … | … | … | … | … | … | … |
| … | … | … | … | … | … | … |
| 24679 | MaleProp+Undetect+Age+DMRI+X3T | 751.94 | 723.7 | 724.3 | -353.9 | 707.7 |
| 24779 | MaleProp+Age+GRE+DMRI+X3T | 752.35 | 724.2 | 724.7 | -354.1 | 708.2 |
| 24794 | Undetect+Age+GRE+DMRI+X3T | 752.42 | 724.2 | 724.8 | -354.1 | 708.2 |
| 24842 | Age+DMRI | 752.60 | 734.9 | 735.2 | -362.5 | 724.9 |
| 25018 | MaleProp+Age+DMRI+X3T | 753.34 | 728.6 | 729.1 | -357.3 | 714.6 |
| 25037 | Age+GRE+DMRI+X3T | 753.44 | 728.7 | 729.2 | -357.4 | 714.7 |
| 25053 | Undetect+Age+DMRI+X3T | 753.50 | 728.8 | 729.3 | -357.4 | 714.8 |
| 25430 | Age+DMRI+X3T | 755.28 | 734.1 | 734.4 | -361.0 | 722.1 |

#### Supplementary Table 11. Year models for Remission

|  | Model | BIC | AIC | AICc | ll | dev |
| --- | --- | --- | --- | --- | --- | --- |
| 3 | PrevTSS+Macro+Undetect+CSI+log(RemCortisolThreshold) | 676.37 | 648.2 | 648.8 | -316.1 | 632.2 |
| 18 | PrevTSS+Macro+Undetect+CSI+log(RemCortisolThreshold)+Year | 679.08 | 647.4 | 648.1 | -314.7 | 629.4 |
| 38 | PrevTSS+MaleProp+Macro+Undetect+CSI+log(RemCortisolThreshold)+Year | 680.21 | 645.0 | 646.0 | -312.5 | 625.0 |
| 113 | PrevTSS+MaleProp+Macro+Undetect+CSI+log(RemCortisolThreshold)+Age+Year | 682.50 | 643.8 | 645.0 | -310.9 | 621.8 |
| 114 | PrevTSS+Undetect+CSI+log(RemCortisolThreshold)+Year | 682.50 | 654.3 | 654.9 | -319.1 | 638.3 |
| 122 | PrevTSS+Macro+Undetect+CSI+log(RemCortisolThreshold)+Age+Year | 682.65 | 647.5 | 648.4 | -313.7 | 627.5 |
| 148 | PrevTSS+Macro+Undetect+CSI+RemCortisolThreshold+Year | 683.02 | 651.3 | 652.1 | -316.7 | 633.3 |
| 177 | PrevTSS+MaleProp+Macro+Undetect+CSI+RemCortisolThreshold+Year | 683.42 | 648.2 | 649.2 | -314.1 | 628.2 |
| … | … | … | … | … | … | … |
| … | … | … | … | … | … | … |
| … | … | … | … | … | … | … |
| 23389 | MaleProp+RemCortisolThreshold+Age+Year | 747.40 | 722.7 | 723.2 | -354.3 | 708.7 |
| 23742 | RemCortisolThreshold+Age+Year | 748.56 | 727.4 | 727.7 | -357.7 | 715.4 |
| 23841 | MaleProp+Year | 748.91 | 731.2 | 731.5 | -360.6 | 721.2 |
| 23850 | Year | 748.94 | 734.8 | 734.9 | -363.4 | 726.8 |
| 23903 | MaleProp+Undetect+Age+Year | 749.13 | 724.4 | 724.9 | -355.2 | 710.4 |
| 24291 | Undetect+Age+Year | 750.54 | 729.3 | 729.7 | -358.7 | 717.3 |
| 24368 | MaleProp+Age+Year | 750.80 | 729.6 | 729.9 | -358.8 | 717.6 |
| 24833 | Age+Year | 752.58 | 734.9 | 735.1 | -362.4 | 724.9 |

#### Supplementary Table 12. All remission models for recurrence

|  | Model | BIC | AIC | AICc | ll | dev |
| --- | --- | --- | --- | --- | --- | --- |
| 1 | Macro+AvgFU+GRE | 431.44 | 413.9 | 414.5 | -200.9 | 401.9 |
| 2 | PrevTSS+Macro+AvgFU+GRE | 432.02 | 411.6 | 412.5 | -198.8 | 397.6 |
| 3 | Macro+RemCortisolThreshold+AvgFU+GRE | 432.63 | 412.2 | 413.1 | -199.1 | 398.2 |
| 4 | PrevTSS+Macro+RemCortisolThreshold+AvgFU+GRE | 433.31 | 410.0 | 411.1 | -197.0 | 394.0 |
| 5 | Macro+log(RemCortisolThreshold)+AvgFU+GRE | 433.43 | 413.0 | 413.9 | -199.5 | 399.0 |
| 6 | Macro+AvgFU+GRE+DMRI | 433.62 | 413.2 | 414.0 | -199.6 | 399.2 |
| 7 | Macro+CSI+AvgFU+GRE | 433.64 | 413.2 | 414.1 | -199.6 | 399.2 |
| 8 | CSI+AvgFU+GRE | 433.68 | 416.1 | 416.8 | -202.1 | 404.1 |
| … | … | … | … | … | … | … |
| … | … | … | … | … | … | … |
| … | … | … | … | … | … | … |
| 52216 | PrevTSS+MaleProp+Macro+Undetect+CSI+log(RemCortisolThreshold)+Age+CT+USS+iMRI+PET+PreCT | 463.41 | 420.5 | 424.8 | -195.3 | 390.5 |
| 52217 | PrevTSS+MaleProp+Macro+Undetect+CSI+log(RemCortisolThreshold)+Age+CT+USS+iMRI+PET+PreCT+Octreotide | 463.41 | 420.5 | 424.8 | -195.3 | 390.5 |
| 52218 | PrevTSS+MaleProp+Macro+Undetect+CSI+log(RemCortisolThreshold)+Age+CT+USS+iMRI+PET+PreCT+IntraopFluorescence | 463.41 | 420.5 | 424.8 | -195.3 | 390.5 |
| 52219 | PrevTSS+MaleProp+Macro+Undetect+CSI+log(RemCortisolThreshold)+Age+CT+USS+iMRI+PET+PreCT+Octreotide+IntraopFluorescence | 463.41 | 420.5 | 424.8 | -195.3 | 390.5 |
| 52220 | PrevTSS+MaleProp+Undetect+log(RemCortisolThreshold)+Age+CT+USS+iMRI+PET+PreCT | 463.53 | 426.2 | 429.3 | -200.1 | 400.2 |
| 52221 | PrevTSS+MaleProp+Undetect+log(RemCortisolThreshold)+Age+CT+USS+iMRI+PET+PreCT+Octreotide | 463.53 | 426.2 | 429.3 | -200.1 | 400.2 |
| 52222 | PrevTSS+MaleProp+Undetect+log(RemCortisolThreshold)+Age+CT+USS+iMRI+PET+PreCT+IntraopFluorescence | 463.53 | 426.2 | 429.3 | -200.1 | 400.2 |
| 52223 | PrevTSS+MaleProp+Undetect+log(RemCortisolThreshold)+Age+CT+USS+iMRI+PET+PreCT+Octreotide+IntraopFluorescence | 463.53 | 426.2 | 429.3 | -200.1 | 400.2 |

#### Supplementary Table 13. Baseline models only for recurrence

|  | Model | BIC | AIC | AICc | ll | dev |
| --- | --- | --- | --- | --- | --- | --- |
| 9 | Macro+AvgFU | 433.83 | 419.2 | 419.6 | -204.6 | 409.2 |
| 23 | PrevTSS+Macro+AvgFU | 434.65 | 417.1 | 417.7 | -202.5 | 405.1 |
| 42 | Macro+RemCortisolThreshold+AvgFU | 435.31 | 417.7 | 418.4 | -202.9 | 405.7 |
| 51 | CSI+AvgFU | 435.40 | 420.7 | 421.2 | -205.4 | 410.7 |
| 66 | Macro+log(RemCortisolThreshold)+AvgFU | 435.88 | 418.3 | 419.0 | -203.2 | 406.3 |
| 71 | Macro+CSI+AvgFU | 435.92 | 418.4 | 419.0 | -203.2 | 406.4 |
| 92 | PrevTSS+CSI+AvgFU | 436.09 | 418.5 | 419.2 | -203.3 | 406.5 |
| 103 | PrevTSS+Macro+RemCortisolThreshold+AvgFU | 436.24 | 415.8 | 416.7 | -200.9 | 401.8 |
| … | … | … | … | … | … | … |
| … | … | … | … | … | … | … |
| … | … | … | … | … | … | … |
| 36836 | PrevTSS+MaleProp+Undetect+Age | 452.04 | 431.6 | 432.5 | -208.8 | 417.6 |
| 37078 | PrevTSS+MaleProp+Undetect+CSI+RemCortisolThreshold+Age | 452.10 | 426.0 | 427.4 | -204.0 | 408.0 |
| 37845 | PrevTSS+MaleProp+Macro+Undetect+CSI+RemCortisolThreshold+Age | 452.31 | 423.3 | 425.1 | -201.7 | 403.3 |
| 38489 | PrevTSS+MaleProp+Undetect+RemCortisolThreshold+Age | 452.51 | 429.2 | 430.3 | -206.6 | 413.2 |
| 38957 | MaleProp+Undetect+log(RemCortisolThreshold)+Age | 452.64 | 432.2 | 433.1 | -209.1 | 418.2 |
| 39975 | PrevTSS+MaleProp+Undetect+CSI+log(RemCortisolThreshold)+Age | 452.96 | 426.8 | 428.3 | -204.4 | 408.8 |
| 40547 | PrevTSS+MaleProp+Macro+Undetect+CSI+log(RemCortisolThreshold)+Age | 453.12 | 424.1 | 425.9 | -202.1 | 404.1 |
| 41250 | PrevTSS+MaleProp+Undetect+log(RemCortisolThreshold)+Age | 453.34 | 430.0 | 431.2 | -207.0 | 414.0 |

#### Supplementary Table 14. Imaging Types in Recurrence

|  | Model | BIC | AIC | AICc | ll | dev |
| --- | --- | --- | --- | --- | --- | --- |
| 9 | Macro+AvgFU | 433.83 | 419.2 | 419.6 | -204.6 | 409.2 |
| 10 | Macro+AvgFU+IntraopFluorescence | 433.83 | 419.2 | 419.6 | -204.6 | 409.2 |
| 11 | Macro+AvgFU+Octreotide | 433.83 | 419.2 | 419.6 | -204.6 | 409.2 |
| 12 | Macro+AvgFU+Octreotide+IntraopFluorescence | 433.83 | 419.2 | 419.6 | -204.6 | 409.2 |
| 24 | PrevTSS+Macro+AvgFU+IntraopFluorescence | 434.65 | 417.1 | 417.7 | -202.5 | 405.1 |
| 25 | PrevTSS+Macro+AvgFU+Octreotide | 434.65 | 417.1 | 417.7 | -202.5 | 405.1 |
| 26 | PrevTSS+Macro+AvgFU+Octreotide+IntraopFluorescence | 434.65 | 417.1 | 417.7 | -202.5 | 405.1 |
| 37 | Macro+AvgFU+USS | 435.17 | 417.6 | 418.2 | -202.8 | 405.6 |
| … | … | … | … | … | … | … |
| … | … | … | … | … | … | … |
| … | … | … | … | … | … | … |
| 52216 | PrevTSS+MaleProp+Macro+Undetect+CSI+log(RemCortisolThreshold)+Age+CT+USS+iMRI+PET+PreCT | 463.41 | 420.5 | 424.8 | -195.3 | 390.5 |
| 52217 | PrevTSS+MaleProp+Macro+Undetect+CSI+log(RemCortisolThreshold)+Age+CT+USS+iMRI+PET+PreCT+Octreotide | 463.41 | 420.5 | 424.8 | -195.3 | 390.5 |
| 52218 | PrevTSS+MaleProp+Macro+Undetect+CSI+log(RemCortisolThreshold)+Age+CT+USS+iMRI+PET+PreCT+IntraopFluorescence | 463.41 | 420.5 | 424.8 | -195.3 | 390.5 |
| 52219 | PrevTSS+MaleProp+Macro+Undetect+CSI+log(RemCortisolThreshold)+Age+CT+USS+iMRI+PET+PreCT+Octreotide+IntraopFluorescence | 463.41 | 420.5 | 424.8 | -195.3 | 390.5 |
| 52220 | PrevTSS+MaleProp+Undetect+log(RemCortisolThreshold)+Age+CT+USS+iMRI+PET+PreCT | 463.53 | 426.2 | 429.3 | -200.1 | 400.2 |
| 52221 | PrevTSS+MaleProp+Undetect+log(RemCortisolThreshold)+Age+CT+USS+iMRI+PET+PreCT+Octreotide | 463.53 | 426.2 | 429.3 | -200.1 | 400.2 |
| 52222 | PrevTSS+MaleProp+Undetect+log(RemCortisolThreshold)+Age+CT+USS+iMRI+PET+PreCT+IntraopFluorescence | 463.53 | 426.2 | 429.3 | -200.1 | 400.2 |
| 52223 | PrevTSS+MaleProp+Undetect+log(RemCortisolThreshold)+Age+CT+USS+iMRI+PET+PreCT+Octreotide+IntraopFluorescence | 463.53 | 426.2 | 429.3 | -200.1 | 400.2 |

#### Supplementary Table 15. MRI Sequences in Recurrence

|  | Model | BIC | AIC | AICc | ll | dev |
| --- | --- | --- | --- | --- | --- | --- |
| 1 | Macro+AvgFU+GRE | 431.44 | 413.9 | 414.5 | -200.9 | 401.9 |
| 2 | PrevTSS+Macro+AvgFU+GRE | 432.02 | 411.6 | 412.5 | -198.8 | 397.6 |
| 3 | Macro+RemCortisolThreshold+AvgFU+GRE | 432.63 | 412.2 | 413.1 | -199.1 | 398.2 |
| 4 | PrevTSS+Macro+RemCortisolThreshold+AvgFU+GRE | 433.31 | 410.0 | 411.1 | -197.0 | 394.0 |
| 5 | Macro+log(RemCortisolThreshold)+AvgFU+GRE | 433.43 | 413.0 | 413.9 | -199.5 | 399.0 |
| 6 | Macro+AvgFU+GRE+DMRI | 433.62 | 413.2 | 414.0 | -199.6 | 399.2 |
| 7 | Macro+CSI+AvgFU+GRE | 433.64 | 413.2 | 414.1 | -199.6 | 399.2 |
| 8 | CSI+AvgFU+GRE | 433.68 | 416.1 | 416.8 | -202.1 | 404.1 |
| … | … | … | … | … | … | … |
| … | … | … | … | … | … | … |
| … | … | … | … | … | … | … |
| 48844 | PrevTSS+MaleProp+Undetect+CSI+log(RemCortisolThreshold)+Age+GRE+DMRI+X3T | 456.53 | 421.9 | 424.6 | -199.0 | 397.9 |
| 48845 | PrevTSS+MaleProp+Macro+Undetect+CSI+RemCortisolThreshold+Age+DMRI+X3T | 456.53 | 421.9 | 424.6 | -199.0 | 397.9 |
| 49286 | PrevTSS+MaleProp+Undetect+RemCortisolThreshold+Age+DMRI+X3T | 456.81 | 427.8 | 429.6 | -203.9 | 407.8 |
| 49395 | PrevTSS+MaleProp+Undetect+log(RemCortisolThreshold)+Age+GRE+DMRI+X3T | 456.88 | 425.1 | 427.3 | -201.5 | 403.1 |
| 49812 | MaleProp+Undetect+log(RemCortisolThreshold)+Age+DMRI+X3T | 457.17 | 431.0 | 432.5 | -206.5 | 413.0 |
| 50053 | PrevTSS+MaleProp+Undetect+CSI+log(RemCortisolThreshold)+Age+DMRI+X3T | 457.38 | 425.6 | 427.8 | -201.8 | 403.6 |
| 50162 | PrevTSS+MaleProp+Macro+Undetect+CSI+log(RemCortisolThreshold)+Age+DMRI+X3T | 457.47 | 422.9 | 425.5 | -199.4 | 398.9 |
| 50463 | PrevTSS+MaleProp+Undetect+log(RemCortisolThreshold)+Age+DMRI+X3T | 457.77 | 428.8 | 430.6 | -204.4 | 408.8 |

#### Supplementary Table 16. Year models for Recurrence

|  | Model | BIC | AIC | AICc | ll | dev |
| --- | --- | --- | --- | --- | --- | --- |
| 9 | Macro+AvgFU | 433.83 | 419.2 | 419.6 | -204.6 | 409.2 |
| 59 | Macro+AvgFU+Year | 435.56 | 418.0 | 418.6 | -203.0 | 406.0 |
| 80 | PrevTSS+Macro+AvgFU+Year | 435.99 | 415.5 | 416.4 | -200.8 | 401.5 |
| 140 | CSI+AvgFU+Year | 436.64 | 419.1 | 419.7 | -203.5 | 407.1 |
| 208 | PrevTSS+CSI+AvgFU+Year | 436.99 | 416.5 | 417.4 | -201.3 | 402.5 |
| 211 | Macro+RemCortisolThreshold+AvgFU+Year | 437.04 | 416.6 | 417.5 | -201.3 | 402.6 |
| 298 | Macro+log(RemCortisolThreshold)+AvgFU+Year | 437.52 | 417.1 | 417.9 | -201.5 | 403.1 |
| 301 | Macro+CSI+AvgFU+Year | 437.54 | 417.1 | 418.0 | -201.5 | 403.1 |
| … | … | … | … | … | … | … |
| … | … | … | … | … | … | … |
| … | … | … | … | … | … | … |
| 39044 | MaleProp+Undetect+RemCortisolThreshold+Age+Year | 452.67 | 429.4 | 430.5 | -206.7 | 413.4 |
| 39233 | PrevTSS+MaleProp+Undetect+CSI+RemCortisolThreshold+Age+Year | 452.73 | 423.7 | 425.5 | -201.9 | 403.7 |
| 40171 | PrevTSS+MaleProp+Macro+Undetect+CSI+RemCortisolThreshold+Age+Year | 453.01 | 421.2 | 423.4 | -199.6 | 399.2 |
| 40584 | PrevTSS+MaleProp+Undetect+RemCortisolThreshold+Age+Year | 453.12 | 427.0 | 428.4 | -204.5 | 409.0 |
| 41204 | MaleProp+Undetect+log(RemCortisolThreshold)+Age+Year | 453.32 | 430.0 | 431.1 | -207.0 | 414.0 |
| 41359 | PrevTSS+MaleProp+Undetect+CSI+log(RemCortisolThreshold)+Age+Year | 453.37 | 424.4 | 426.2 | -202.2 | 404.4 |
| 42153 | PrevTSS+MaleProp+Macro+Undetect+CSI+log(RemCortisolThreshold)+Age+Year | 453.63 | 421.8 | 424.0 | -199.9 | 399.8 |
| 42432 | PrevTSS+MaleProp+Undetect+log(RemCortisolThreshold)+Age+Year | 453.73 | 427.6 | 429.0 | -204.8 | 409.6 |

#### Supplementary Table 17. Imaging Types without Macro for Remission

|  | Model | BIC | AIC | AICc | ll | dev |
| --- | --- | --- | --- | --- | --- | --- |
| 28 | PrevTSS+Undetect+CSI+log(RemCortisolThreshold)+Octreotide | 679.62 | 651.4 | 652.0 | -317.7 | 635.4 |
| 43 | PrevTSS+Undetect+CSI+log(RemCortisolThreshold) | 680.40 | 655.7 | 656.2 | -320.8 | 641.7 |
| 67 | PrevTSS+Undetect+CSI+log(RemCortisolThreshold)+USS+Octreotide | 681.29 | 649.6 | 650.4 | -315.8 | 631.6 |
| 85 | PrevTSS+MaleProp+Undetect+CSI+log(RemCortisolThreshold)+Octreotide | 681.97 | 650.3 | 651.0 | -316.1 | 632.3 |
| 88 | PrevTSS+Undetect+CSI+log(RemCortisolThreshold)+USS | 682.06 | 653.9 | 654.5 | -318.9 | 637.9 |
| 107 | PrevTSS+MaleProp+Undetect+CSI+log(RemCortisolThreshold) | 682.46 | 654.3 | 654.8 | -319.1 | 638.3 |
| 108 | PrevTSS+Undetect+CSI+log(RemCortisolThreshold)+Octreotide+IntraopFluorescence | 682.47 | 650.8 | 651.5 | -316.4 | 632.8 |
| 119 | PrevTSS+Undetect+CSI+log(RemCortisolThreshold)+PET+Octreotide | 682.59 | 650.9 | 651.6 | -316.4 | 632.9 |
| … | … | … | … | … | … | … |
| … | … | … | … | … | … | … |
| … | … | … | … | … | … | … |
| 26104 | Undetect+Age+CT+USS+iMRI+PET+PreCT+Octreotide+IntraopFluorescence | 764.42 | 722.3 | 723.6 | -349.2 | 698.3 |
| 26105 | Undetect+Age+CT+USS+iMRI+PET+PreCT+IntraopFluorescence | 764.74 | 726.1 | 727.2 | -352.0 | 704.1 |
| 26106 | MaleProp+Age+CT+USS+iMRI+PET+PreCT+IntraopFluorescence | 764.91 | 726.3 | 727.4 | -352.1 | 704.3 |
| 26107 | Age+CT+iMRI+PET+PreCT+IntraopFluorescence | 765.01 | 733.3 | 734.1 | -357.7 | 715.3 |
| 26108 | Age+CT+iMRI+PET+PreCT+Octreotide+IntraopFluorescence | 765.06 | 729.9 | 730.8 | -354.9 | 709.9 |
| 26109 | MaleProp+Age+CT+USS+iMRI+PET+PreCT+Octreotide+IntraopFluorescence | 765.31 | 723.2 | 724.5 | -349.6 | 699.2 |
| 26110 | Age+CT+USS+iMRI+PET+PreCT+IntraopFluorescence | 766.79 | 731.6 | 732.5 | -355.8 | 711.6 |
| 26111 | Age+CT+USS+iMRI+PET+PreCT+Octreotide+IntraopFluorescence | 766.83 | 728.2 | 729.3 | -353.1 | 706.2 |

#### Supplementary Table 18. Imaging Types without Undetect for Remission

|  | Model | BIC | AIC | AICc | ll | dev |
| --- | --- | --- | --- | --- | --- | --- |
| 2211 | PrevTSS+CSI+log(RemCortisolThreshold)+Octreotide | 694.50 | 669.8 | 670.3 | -327.9 | 655.8 |
| 2277 | PrevTSS+CSI+log(RemCortisolThreshold) | 694.69 | 673.5 | 673.8 | -330.7 | 661.5 |
| 2547 | PrevTSS+MaleProp+CSI+log(RemCortisolThreshold) | 695.66 | 671.0 | 671.4 | -328.5 | 657.0 |
| 2590 | PrevTSS+MaleProp+CSI+log(RemCortisolThreshold)+Octreotide | 695.82 | 667.6 | 668.2 | -325.8 | 651.6 |
| 2639 | PrevTSS+Macro+CSI+log(RemCortisolThreshold)+Octreotide | 696.13 | 667.9 | 668.5 | -326.0 | 651.9 |
| 2675 | PrevTSS+CSI+log(RemCortisolThreshold)+USS+Octreotide | 696.29 | 668.1 | 668.7 | -326.0 | 652.1 |
| 2685 | PrevTSS+Macro+CSI+log(RemCortisolThreshold) | 696.32 | 671.6 | 672.1 | -328.8 | 657.6 |
| 2718 | PrevTSS+CSI+log(RemCortisolThreshold)+USS | 696.46 | 671.8 | 672.2 | -328.9 | 657.8 |
| … | … | … | … | … | … | … |
| … | … | … | … | … | … | … |
| … | … | … | … | … | … | … |
| 26102 | Age+CT+USS+iMRI+PreCT+Octreotide+IntraopFluorescence | 763.94 | 728.8 | 729.7 | -354.4 | 708.8 |
| 26103 | MaleProp+CT+USS+iMRI+PET+PreCT+Octreotide+IntraopFluorescence | 763.97 | 725.3 | 726.4 | -351.7 | 703.3 |
| 26106 | MaleProp+Age+CT+USS+iMRI+PET+PreCT+IntraopFluorescence | 764.91 | 726.3 | 727.4 | -352.1 | 704.3 |
| 26107 | Age+CT+iMRI+PET+PreCT+IntraopFluorescence | 765.01 | 733.3 | 734.1 | -357.7 | 715.3 |
| 26108 | Age+CT+iMRI+PET+PreCT+Octreotide+IntraopFluorescence | 765.06 | 729.9 | 730.8 | -354.9 | 709.9 |
| 26109 | MaleProp+Age+CT+USS+iMRI+PET+PreCT+Octreotide+IntraopFluorescence | 765.31 | 723.2 | 724.5 | -349.6 | 699.2 |
| 26110 | Age+CT+USS+iMRI+PET+PreCT+IntraopFluorescence | 766.79 | 731.6 | 732.5 | -355.8 | 711.6 |
| 26111 | Age+CT+USS+iMRI+PET+PreCT+Octreotide+IntraopFluorescence | 766.83 | 728.2 | 729.3 | -353.1 | 706.2 |

#### Supplementary Table 19. Imaging Types without Macro or Undetect for Remission

|  | Model | BIC | AIC | AICc | ll | dev |
| --- | --- | --- | --- | --- | --- | --- |
| 2211 | PrevTSS+CSI+log(RemCortisolThreshold)+Octreotide | 694.50 | 669.8 | 670.3 | -327.9 | 655.8 |
| 2277 | PrevTSS+CSI+log(RemCortisolThreshold) | 694.69 | 673.5 | 673.8 | -330.7 | 661.5 |
| 2547 | PrevTSS+MaleProp+CSI+log(RemCortisolThreshold) | 695.66 | 671.0 | 671.4 | -328.5 | 657.0 |
| 2590 | PrevTSS+MaleProp+CSI+log(RemCortisolThreshold)+Octreotide | 695.82 | 667.6 | 668.2 | -325.8 | 651.6 |
| 2675 | PrevTSS+CSI+log(RemCortisolThreshold)+USS+Octreotide | 696.29 | 668.1 | 668.7 | -326.0 | 652.1 |
| 2718 | PrevTSS+CSI+log(RemCortisolThreshold)+USS | 696.46 | 671.8 | 672.2 | -328.9 | 657.8 |
| 2938 | PrevTSS+MaleProp+CSI+log(RemCortisolThreshold)+Age | 697.32 | 669.1 | 669.7 | -326.6 | 653.1 |
| 2972 | PrevTSS+MaleProp+CSI+log(RemCortisolThreshold)+Age+Octreotide | 697.43 | 665.7 | 666.5 | -323.9 | 647.7 |
| … | … | … | … | … | … | … |
| … | … | … | … | … | … | … |
| … | … | … | … | … | … | … |
| 26102 | Age+CT+USS+iMRI+PreCT+Octreotide+IntraopFluorescence | 763.94 | 728.8 | 729.7 | -354.4 | 708.8 |
| 26103 | MaleProp+CT+USS+iMRI+PET+PreCT+Octreotide+IntraopFluorescence | 763.97 | 725.3 | 726.4 | -351.7 | 703.3 |
| 26106 | MaleProp+Age+CT+USS+iMRI+PET+PreCT+IntraopFluorescence | 764.91 | 726.3 | 727.4 | -352.1 | 704.3 |
| 26107 | Age+CT+iMRI+PET+PreCT+IntraopFluorescence | 765.01 | 733.3 | 734.1 | -357.7 | 715.3 |
| 26108 | Age+CT+iMRI+PET+PreCT+Octreotide+IntraopFluorescence | 765.06 | 729.9 | 730.8 | -354.9 | 709.9 |
| 26109 | MaleProp+Age+CT+USS+iMRI+PET+PreCT+Octreotide+IntraopFluorescence | 765.31 | 723.2 | 724.5 | -349.6 | 699.2 |
| 26110 | Age+CT+USS+iMRI+PET+PreCT+IntraopFluorescence | 766.79 | 731.6 | 732.5 | -355.8 | 711.6 |
| 26111 | Age+CT+USS+iMRI+PET+PreCT+Octreotide+IntraopFluorescence | 766.83 | 728.2 | 729.3 | -353.1 | 706.2 |

#### Supplementary Table 20. MRI Sequences without Macro for Remission

|  | Model | BIC | AIC | AICc | ll | dev |
| --- | --- | --- | --- | --- | --- | --- |
| 32 | PrevTSS+Undetect+CSI+log(RemCortisolThreshold)+GRE | 679.75 | 651.5 | 652.1 | -317.8 | 635.5 |
| 43 | PrevTSS+Undetect+CSI+log(RemCortisolThreshold) | 680.40 | 655.7 | 656.2 | -320.8 | 641.7 |
| 60 | PrevTSS+Undetect+CSI+log(RemCortisolThreshold)+GRE+DMRI | 681.09 | 649.4 | 650.1 | -315.7 | 631.4 |
| 78 | PrevTSS+MaleProp+Undetect+CSI+log(RemCortisolThreshold)+GRE | 681.76 | 650.1 | 650.8 | -316.0 | 632.1 |
| 107 | PrevTSS+MaleProp+Undetect+CSI+log(RemCortisolThreshold) | 682.46 | 654.3 | 654.8 | -319.1 | 638.3 |
| 120 | PrevTSS+Undetect+CSI+RemCortisolThreshold+GRE | 682.64 | 654.4 | 655.0 | -319.2 | 638.4 |
| 155 | PrevTSS+Undetect+CSI+log(RemCortisolThreshold)+Age+GRE | 683.09 | 651.4 | 652.1 | -316.7 | 633.4 |
| 157 | PrevTSS+Undetect+CSI+log(RemCortisolThreshold)+DMRI | 683.12 | 654.9 | 655.5 | -319.5 | 638.9 |
| … | … | … | … | … | … | … |
| … | … | … | … | … | … | … |
| … | … | … | … | … | … | … |
| 24679 | MaleProp+Undetect+Age+DMRI+X3T | 751.94 | 723.7 | 724.3 | -353.9 | 707.7 |
| 24779 | MaleProp+Age+GRE+DMRI+X3T | 752.35 | 724.2 | 724.7 | -354.1 | 708.2 |
| 24794 | Undetect+Age+GRE+DMRI+X3T | 752.42 | 724.2 | 724.8 | -354.1 | 708.2 |
| 24842 | Age+DMRI | 752.60 | 734.9 | 735.2 | -362.5 | 724.9 |
| 25018 | MaleProp+Age+DMRI+X3T | 753.34 | 728.6 | 729.1 | -357.3 | 714.6 |
| 25037 | Age+GRE+DMRI+X3T | 753.44 | 728.7 | 729.2 | -357.4 | 714.7 |
| 25053 | Undetect+Age+DMRI+X3T | 753.50 | 728.8 | 729.3 | -357.4 | 714.8 |
| 25430 | Age+DMRI+X3T | 755.28 | 734.1 | 734.4 | -361.0 | 722.1 |

#### Supplementary Table 21. MRI Sequences without Undetect for Remission

|  | Model | BIC | AIC | AICc | ll | dev |
| --- | --- | --- | --- | --- | --- | --- |
| 1788 | PrevTSS+CSI+log(RemCortisolThreshold)+GRE | 692.77 | 668.1 | 668.5 | -327.0 | 654.1 |
| 2038 | PrevTSS+MaleProp+CSI+log(RemCortisolThreshold)+GRE | 693.77 | 665.6 | 666.2 | -324.8 | 649.6 |
| 2172 | PrevTSS+Macro+CSI+log(RemCortisolThreshold)+GRE | 694.32 | 666.1 | 666.7 | -325.1 | 650.1 |
| 2277 | PrevTSS+CSI+log(RemCortisolThreshold) | 694.69 | 673.5 | 673.8 | -330.7 | 661.5 |
| 2285 | PrevTSS+CSI+log(RemCortisolThreshold)+GRE+DMRI | 694.70 | 666.5 | 667.1 | -325.2 | 650.5 |
| 2363 | PrevTSS+MaleProp+Macro+CSI+log(RemCortisolThreshold)+GRE | 694.99 | 663.3 | 664.1 | -322.7 | 645.3 |
| 2416 | PrevTSS+CSI+RemCortisolThreshold+GRE | 695.19 | 670.5 | 670.9 | -328.2 | 656.5 |
| 2502 | PrevTSS+MaleProp+CSI+RemCortisolThreshold+GRE | 695.51 | 667.3 | 667.9 | -325.7 | 651.3 |
| … | … | … | … | … | … | … |
| … | … | … | … | … | … | … |
| … | … | … | … | … | … | … |
| 24551 | Age+X3T | 751.44 | 733.8 | 734.0 | -361.9 | 723.8 |
| 24613 | MaleProp+DMRI+X3T | 751.65 | 730.4 | 730.8 | -359.2 | 718.4 |
| 24615 | DMRI+X3T | 751.66 | 734.0 | 734.2 | -362.0 | 724.0 |
| 24779 | MaleProp+Age+GRE+DMRI+X3T | 752.35 | 724.2 | 724.7 | -354.1 | 708.2 |
| 24842 | Age+DMRI | 752.60 | 734.9 | 735.2 | -362.5 | 724.9 |
| 25018 | MaleProp+Age+DMRI+X3T | 753.34 | 728.6 | 729.1 | -357.3 | 714.6 |
| 25037 | Age+GRE+DMRI+X3T | 753.44 | 728.7 | 729.2 | -357.4 | 714.7 |
| 25430 | Age+DMRI+X3T | 755.28 | 734.1 | 734.4 | -361.0 | 722.1 |

#### Supplementary Table 22. MRI Sequences without Macro or Undetect for Remission

|  | Model | BIC | AIC | AICc | ll | dev |
| --- | --- | --- | --- | --- | --- | --- |
| 1788 | PrevTSS+CSI+log(RemCortisolThreshold)+GRE | 692.77 | 668.1 | 668.5 | -327.0 | 654.1 |
| 2038 | PrevTSS+MaleProp+CSI+log(RemCortisolThreshold)+GRE | 693.77 | 665.6 | 666.2 | -324.8 | 649.6 |
| 2277 | PrevTSS+CSI+log(RemCortisolThreshold) | 694.69 | 673.5 | 673.8 | -330.7 | 661.5 |
| 2285 | PrevTSS+CSI+log(RemCortisolThreshold)+GRE+DMRI | 694.70 | 666.5 | 667.1 | -325.2 | 650.5 |
| 2416 | PrevTSS+CSI+RemCortisolThreshold+GRE | 695.19 | 670.5 | 670.9 | -328.2 | 656.5 |
| 2502 | PrevTSS+MaleProp+CSI+RemCortisolThreshold+GRE | 695.51 | 667.3 | 667.9 | -325.7 | 651.3 |
| 2547 | PrevTSS+MaleProp+CSI+log(RemCortisolThreshold) | 695.66 | 671.0 | 671.4 | -328.5 | 657.0 |
| 2569 | PrevTSS+MaleProp+CSI+log(RemCortisolThreshold)+GRE+DMRI | 695.76 | 664.1 | 664.8 | -323.0 | 646.1 |
| … | … | … | … | … | … | … |
| … | … | … | … | … | … | … |
| … | … | … | … | … | … | … |
| 24551 | Age+X3T | 751.44 | 733.8 | 734.0 | -361.9 | 723.8 |
| 24613 | MaleProp+DMRI+X3T | 751.65 | 730.4 | 730.8 | -359.2 | 718.4 |
| 24615 | DMRI+X3T | 751.66 | 734.0 | 734.2 | -362.0 | 724.0 |
| 24779 | MaleProp+Age+GRE+DMRI+X3T | 752.35 | 724.2 | 724.7 | -354.1 | 708.2 |
| 24842 | Age+DMRI | 752.60 | 734.9 | 735.2 | -362.5 | 724.9 |
| 25018 | MaleProp+Age+DMRI+X3T | 753.34 | 728.6 | 729.1 | -357.3 | 714.6 |
| 25037 | Age+GRE+DMRI+X3T | 753.44 | 728.7 | 729.2 | -357.4 | 714.7 |
| 25430 | Age+DMRI+X3T | 755.28 | 734.1 | 734.4 | -361.0 | 722.1 |

#### Supplementary Table 23. Year without Macro for Remission

|  | Model | BIC | AIC | AICc | ll | dev |
| --- | --- | --- | --- | --- | --- | --- |
| 43 | PrevTSS+Undetect+CSI+log(RemCortisolThreshold) | 680.40 | 655.7 | 656.2 | -320.8 | 641.7 |
| 107 | PrevTSS+MaleProp+Undetect+CSI+log(RemCortisolThreshold) | 682.46 | 654.3 | 654.8 | -319.1 | 638.3 |
| 114 | PrevTSS+Undetect+CSI+log(RemCortisolThreshold)+Year | 682.50 | 654.3 | 654.9 | -319.1 | 638.3 |
| 185 | PrevTSS+Undetect+CSI+log(RemCortisolThreshold)+Age | 683.56 | 655.4 | 655.9 | -319.7 | 639.4 |
| 203 | PrevTSS+Undetect+CSI+RemCortisolThreshold | 683.84 | 659.1 | 659.6 | -322.6 | 645.1 |
| 213 | PrevTSS+MaleProp+Undetect+CSI+log(RemCortisolThreshold)+Age | 683.96 | 652.3 | 653.0 | -317.1 | 634.3 |
| 226 | PrevTSS+MaleProp+Undetect+CSI+log(RemCortisolThreshold)+Year | 684.15 | 652.5 | 653.2 | -317.2 | 634.5 |
| 331 | PrevTSS+MaleProp+Undetect+CSI+RemCortisolThreshold | 685.30 | 657.1 | 657.7 | -320.5 | 641.1 |
| … | … | … | … | … | … | … |
| … | … | … | … | … | … | … |
| … | … | … | … | … | … | … |
| 23742 | RemCortisolThreshold+Age+Year | 748.56 | 727.4 | 727.7 | -357.7 | 715.4 |
| 23821 | Age | 748.84 | 734.7 | 734.8 | -363.3 | 726.7 |
| 23841 | MaleProp+Year | 748.91 | 731.2 | 731.5 | -360.6 | 721.2 |
| 23850 | Year | 748.94 | 734.8 | 734.9 | -363.4 | 726.8 |
| 23903 | MaleProp+Undetect+Age+Year | 749.13 | 724.4 | 724.9 | -355.2 | 710.4 |
| 24291 | Undetect+Age+Year | 750.54 | 729.3 | 729.7 | -358.7 | 717.3 |
| 24368 | MaleProp+Age+Year | 750.80 | 729.6 | 729.9 | -358.8 | 717.6 |
| 24833 | Age+Year | 752.58 | 734.9 | 735.1 | -362.4 | 724.9 |

#### Supplementary Table 24. Year without Undetect for Remission

|  | Model | BIC | AIC | AICc | ll | dev |
| --- | --- | --- | --- | --- | --- | --- |
| 2277 | PrevTSS+CSI+log(RemCortisolThreshold) | 694.69 | 673.5 | 673.8 | -330.7 | 661.5 |
| 2547 | PrevTSS+MaleProp+CSI+log(RemCortisolThreshold) | 695.66 | 671.0 | 671.4 | -328.5 | 657.0 |
| 2685 | PrevTSS+Macro+CSI+log(RemCortisolThreshold) | 696.32 | 671.6 | 672.1 | -328.8 | 657.6 |
| 2845 | PrevTSS+MaleProp+Macro+CSI+log(RemCortisolThreshold) | 696.95 | 668.7 | 669.3 | -326.4 | 652.7 |
| 2938 | PrevTSS+MaleProp+CSI+log(RemCortisolThreshold)+Age | 697.32 | 669.1 | 669.7 | -326.6 | 653.1 |
| 3075 | PrevTSS+CSI+RemCortisolThreshold | 697.73 | 676.5 | 676.9 | -332.3 | 664.5 |
| 3155 | PrevTSS+MaleProp+CSI+RemCortisolThreshold | 698.00 | 673.3 | 673.7 | -329.6 | 659.3 |
| 3202 | PrevTSS+CSI+log(RemCortisolThreshold)+Age | 698.13 | 673.4 | 673.9 | -329.7 | 659.4 |
| … | … | … | … | … | … | … |
| … | … | … | … | … | … | … |
| … | … | … | … | … | … | … |
| 23280 | MaleProp+Age | 747.05 | 729.4 | 729.6 | -359.7 | 719.4 |
| 23389 | MaleProp+RemCortisolThreshold+Age+Year | 747.40 | 722.7 | 723.2 | -354.3 | 708.7 |
| 23742 | RemCortisolThreshold+Age+Year | 748.56 | 727.4 | 727.7 | -357.7 | 715.4 |
| 23821 | Age | 748.84 | 734.7 | 734.8 | -363.3 | 726.7 |
| 23841 | MaleProp+Year | 748.91 | 731.2 | 731.5 | -360.6 | 721.2 |
| 23850 | Year | 748.94 | 734.8 | 734.9 | -363.4 | 726.8 |
| 24368 | MaleProp+Age+Year | 750.80 | 729.6 | 729.9 | -358.8 | 717.6 |
| 24833 | Age+Year | 752.58 | 734.9 | 735.1 | -362.4 | 724.9 |

#### Supplementary Table 25. Year without Macro or Undetect for Remission

|  | Model | BIC | AIC | AICc | ll | dev |
| --- | --- | --- | --- | --- | --- | --- |
| 2277 | PrevTSS+CSI+log(RemCortisolThreshold) | 694.69 | 673.5 | 673.8 | -330.7 | 661.5 |
| 2547 | PrevTSS+MaleProp+CSI+log(RemCortisolThreshold) | 695.66 | 671.0 | 671.4 | -328.5 | 657.0 |
| 2938 | PrevTSS+MaleProp+CSI+log(RemCortisolThreshold)+Age | 697.32 | 669.1 | 669.7 | -326.6 | 653.1 |
| 3075 | PrevTSS+CSI+RemCortisolThreshold | 697.73 | 676.5 | 676.9 | -332.3 | 664.5 |
| 3155 | PrevTSS+MaleProp+CSI+RemCortisolThreshold | 698.00 | 673.3 | 673.7 | -329.6 | 659.3 |
| 3202 | PrevTSS+CSI+log(RemCortisolThreshold)+Age | 698.13 | 673.4 | 673.9 | -329.7 | 659.4 |
| 3217 | PrevTSS+CSI+log(RemCortisolThreshold)+Year | 698.20 | 673.5 | 674.0 | -329.7 | 659.5 |
| 3440 | PrevTSS+MaleProp+CSI+log(RemCortisolThreshold)+Year | 698.93 | 670.7 | 671.3 | -327.4 | 654.7 |
| … | … | … | … | … | … | … |
| … | … | … | … | … | … | … |
| … | … | … | … | … | … | … |
| 23280 | MaleProp+Age | 747.05 | 729.4 | 729.6 | -359.7 | 719.4 |
| 23389 | MaleProp+RemCortisolThreshold+Age+Year | 747.40 | 722.7 | 723.2 | -354.3 | 708.7 |
| 23742 | RemCortisolThreshold+Age+Year | 748.56 | 727.4 | 727.7 | -357.7 | 715.4 |
| 23821 | Age | 748.84 | 734.7 | 734.8 | -363.3 | 726.7 |
| 23841 | MaleProp+Year | 748.91 | 731.2 | 731.5 | -360.6 | 721.2 |
| 23850 | Year | 748.94 | 734.8 | 734.9 | -363.4 | 726.8 |
| 24368 | MaleProp+Age+Year | 750.80 | 729.6 | 729.9 | -358.8 | 717.6 |
| 24833 | Age+Year | 752.58 | 734.9 | 735.1 | -362.4 | 724.9 |

#### Supplementary Table 26. Imaging Types without Macro for Recurrence

|  | Model | BIC | AIC | AICc | ll | dev |
| --- | --- | --- | --- | --- | --- | --- |
| 51 | CSI+AvgFU | 435.40 | 420.7 | 421.2 | -205.4 | 410.7 |
| 52 | CSI+AvgFU+IntraopFluorescence | 435.40 | 420.7 | 421.2 | -205.4 | 410.7 |
| 53 | CSI+AvgFU+Octreotide | 435.40 | 420.7 | 421.2 | -205.4 | 410.7 |
| 54 | CSI+AvgFU+Octreotide+IntraopFluorescence | 435.40 | 420.7 | 421.2 | -205.4 | 410.7 |
| 92 | PrevTSS+CSI+AvgFU | 436.09 | 418.5 | 419.2 | -203.3 | 406.5 |
| 93 | PrevTSS+CSI+AvgFU+IntraopFluorescence | 436.09 | 418.5 | 419.2 | -203.3 | 406.5 |
| 94 | PrevTSS+CSI+AvgFU+Octreotide | 436.09 | 418.5 | 419.2 | -203.3 | 406.5 |
| 95 | PrevTSS+CSI+AvgFU+Octreotide+IntraopFluorescence | 436.09 | 418.5 | 419.2 | -203.3 | 406.5 |
| … | … | … | … | … | … | … |
| … | … | … | … | … | … | … |
| … | … | … | … | … | … | … |
| 52212 | PrevTSS+MaleProp+Undetect+CSI+log(RemCortisolThreshold)+Age+CT+USS+iMRI+PET+PreCT | 463.17 | 423.0 | 426.7 | -197.5 | 395.0 |
| 52213 | PrevTSS+MaleProp+Undetect+CSI+log(RemCortisolThreshold)+Age+CT+USS+iMRI+PET+PreCT+Octreotide | 463.17 | 423.0 | 426.7 | -197.5 | 395.0 |
| 52214 | PrevTSS+MaleProp+Undetect+CSI+log(RemCortisolThreshold)+Age+CT+USS+iMRI+PET+PreCT+IntraopFluorescence | 463.17 | 423.0 | 426.7 | -197.5 | 395.0 |
| 52215 | PrevTSS+MaleProp+Undetect+CSI+log(RemCortisolThreshold)+Age+CT+USS+iMRI+PET+PreCT+Octreotide+IntraopFluorescence | 463.17 | 423.0 | 426.7 | -197.5 | 395.0 |
| 52220 | PrevTSS+MaleProp+Undetect+log(RemCortisolThreshold)+Age+CT+USS+iMRI+PET+PreCT | 463.53 | 426.2 | 429.3 | -200.1 | 400.2 |
| 52221 | PrevTSS+MaleProp+Undetect+log(RemCortisolThreshold)+Age+CT+USS+iMRI+PET+PreCT+Octreotide | 463.53 | 426.2 | 429.3 | -200.1 | 400.2 |
| 52222 | PrevTSS+MaleProp+Undetect+log(RemCortisolThreshold)+Age+CT+USS+iMRI+PET+PreCT+IntraopFluorescence | 463.53 | 426.2 | 429.3 | -200.1 | 400.2 |
| 52223 | PrevTSS+MaleProp+Undetect+log(RemCortisolThreshold)+Age+CT+USS+iMRI+PET+PreCT+Octreotide+IntraopFluorescence | 463.53 | 426.2 | 429.3 | -200.1 | 400.2 |

#### Supplementary Table 27. Imaging Types without Undetect for Recurrence

|  | Model | BIC | AIC | AICc | ll | dev |
| --- | --- | --- | --- | --- | --- | --- |
| 9 | Macro+AvgFU | 433.83 | 419.2 | 419.6 | -204.6 | 409.2 |
| 10 | Macro+AvgFU+IntraopFluorescence | 433.83 | 419.2 | 419.6 | -204.6 | 409.2 |
| 11 | Macro+AvgFU+Octreotide | 433.83 | 419.2 | 419.6 | -204.6 | 409.2 |
| 12 | Macro+AvgFU+Octreotide+IntraopFluorescence | 433.83 | 419.2 | 419.6 | -204.6 | 409.2 |
| 23 | PrevTSS+Macro+AvgFU | 434.65 | 417.1 | 417.7 | -202.5 | 405.1 |
| 24 | PrevTSS+Macro+AvgFU+IntraopFluorescence | 434.65 | 417.1 | 417.7 | -202.5 | 405.1 |
| 25 | PrevTSS+Macro+AvgFU+Octreotide | 434.65 | 417.1 | 417.7 | -202.5 | 405.1 |
| 26 | PrevTSS+Macro+AvgFU+Octreotide+IntraopFluorescence | 434.65 | 417.1 | 417.7 | -202.5 | 405.1 |
| … | … | … | … | … | … | … |
| … | … | … | … | … | … | … |
| … | … | … | … | … | … | … |
| 52008 | PrevTSS+MaleProp+log(RemCortisolThreshold)+Age+CT+USS+iMRI+PET+PreCT | 460.68 | 426.1 | 428.7 | -201.0 | 402.1 |
| 52009 | PrevTSS+MaleProp+log(RemCortisolThreshold)+Age+CT+USS+iMRI+PET+PreCT+Octreotide | 460.68 | 426.1 | 428.7 | -201.0 | 402.1 |
| 52010 | PrevTSS+MaleProp+log(RemCortisolThreshold)+Age+CT+USS+iMRI+PET+PreCT+IntraopFluorescence | 460.68 | 426.1 | 428.7 | -201.0 | 402.1 |
| 52011 | PrevTSS+MaleProp+log(RemCortisolThreshold)+Age+CT+USS+iMRI+PET+PreCT+Octreotide+IntraopFluorescence | 460.68 | 426.1 | 428.7 | -201.0 | 402.1 |
| 52024 | PrevTSS+MaleProp+Macro+CSI+log(RemCortisolThreshold)+Age+CT+USS+iMRI+PET+PreCT | 460.76 | 420.6 | 424.3 | -196.3 | 392.6 |
| 52025 | PrevTSS+MaleProp+Macro+CSI+log(RemCortisolThreshold)+Age+CT+USS+iMRI+PET+PreCT+Octreotide | 460.76 | 420.6 | 424.3 | -196.3 | 392.6 |
| 52026 | PrevTSS+MaleProp+Macro+CSI+log(RemCortisolThreshold)+Age+CT+USS+iMRI+PET+PreCT+IntraopFluorescence | 460.76 | 420.6 | 424.3 | -196.3 | 392.6 |
| 52027 | PrevTSS+MaleProp+Macro+CSI+log(RemCortisolThreshold)+Age+CT+USS+iMRI+PET+PreCT+Octreotide+IntraopFluorescence | 460.76 | 420.6 | 424.3 | -196.3 | 392.6 |

#### Supplementary Table 28. Imaging Types without Macro or Undetect for Recurrence

|  | Model | BIC | AIC | AICc | ll | dev |
| --- | --- | --- | --- | --- | --- | --- |
| 51 | CSI+AvgFU | 435.40 | 420.7 | 421.2 | -205.4 | 410.7 |
| 52 | CSI+AvgFU+IntraopFluorescence | 435.40 | 420.7 | 421.2 | -205.4 | 410.7 |
| 53 | CSI+AvgFU+Octreotide | 435.40 | 420.7 | 421.2 | -205.4 | 410.7 |
| 54 | CSI+AvgFU+Octreotide+IntraopFluorescence | 435.40 | 420.7 | 421.2 | -205.4 | 410.7 |
| 92 | PrevTSS+CSI+AvgFU | 436.09 | 418.5 | 419.2 | -203.3 | 406.5 |
| 93 | PrevTSS+CSI+AvgFU+IntraopFluorescence | 436.09 | 418.5 | 419.2 | -203.3 | 406.5 |
| 94 | PrevTSS+CSI+AvgFU+Octreotide | 436.09 | 418.5 | 419.2 | -203.3 | 406.5 |
| 95 | PrevTSS+CSI+AvgFU+Octreotide+IntraopFluorescence | 436.09 | 418.5 | 419.2 | -203.3 | 406.5 |
| … | … | … | … | … | … | … |
| … | … | … | … | … | … | … |
| … | … | … | … | … | … | … |
| 51940 | PrevTSS+MaleProp+CSI+log(RemCortisolThreshold)+Age+CT+USS+iMRI+PET+PreCT | 460.34 | 423.0 | 426.1 | -198.5 | 397.0 |
| 51941 | PrevTSS+MaleProp+CSI+log(RemCortisolThreshold)+Age+CT+USS+iMRI+PET+PreCT+Octreotide | 460.34 | 423.0 | 426.1 | -198.5 | 397.0 |
| 51942 | PrevTSS+MaleProp+CSI+log(RemCortisolThreshold)+Age+CT+USS+iMRI+PET+PreCT+IntraopFluorescence | 460.34 | 423.0 | 426.1 | -198.5 | 397.0 |
| 51943 | PrevTSS+MaleProp+CSI+log(RemCortisolThreshold)+Age+CT+USS+iMRI+PET+PreCT+Octreotide+IntraopFluorescence | 460.34 | 423.0 | 426.1 | -198.5 | 397.0 |
| 52008 | PrevTSS+MaleProp+log(RemCortisolThreshold)+Age+CT+USS+iMRI+PET+PreCT | 460.68 | 426.1 | 428.7 | -201.0 | 402.1 |
| 52009 | PrevTSS+MaleProp+log(RemCortisolThreshold)+Age+CT+USS+iMRI+PET+PreCT+Octreotide | 460.68 | 426.1 | 428.7 | -201.0 | 402.1 |
| 52010 | PrevTSS+MaleProp+log(RemCortisolThreshold)+Age+CT+USS+iMRI+PET+PreCT+IntraopFluorescence | 460.68 | 426.1 | 428.7 | -201.0 | 402.1 |
| 52011 | PrevTSS+MaleProp+log(RemCortisolThreshold)+Age+CT+USS+iMRI+PET+PreCT+Octreotide+IntraopFluorescence | 460.68 | 426.1 | 428.7 | -201.0 | 402.1 |

#### Supplementary Table 29. MRI Sequences without Macro for Recurrence

|  | Model | BIC | AIC | AICc | ll | dev |
| --- | --- | --- | --- | --- | --- | --- |
| 8 | CSI+AvgFU+GRE | 433.68 | 416.1 | 416.8 | -202.1 | 404.1 |
| 14 | PrevTSS+CSI+AvgFU+GRE | 434.04 | 413.6 | 414.5 | -199.8 | 399.6 |
| 27 | CSI+RemCortisolThreshold+AvgFU+GRE | 434.68 | 414.2 | 415.1 | -200.1 | 400.2 |
| 31 | AvgFU+GRE | 434.83 | 420.2 | 420.6 | -205.1 | 410.2 |
| 32 | PrevTSS+AvgFU+GRE | 434.96 | 417.4 | 418.0 | -202.7 | 405.4 |
| 36 | PrevTSS+CSI+RemCortisolThreshold+AvgFU+GRE | 435.10 | 411.8 | 412.9 | -197.9 | 395.8 |
| 51 | CSI+AvgFU | 435.40 | 420.7 | 421.2 | -205.4 | 410.7 |
| 56 | CSI+AvgFU+GRE+DMRI | 435.46 | 415.0 | 415.9 | -200.5 | 401.0 |
| … | … | … | … | … | … | … |
| … | … | … | … | … | … | … |
| … | … | … | … | … | … | … |
| 48822 | MaleProp+Undetect+CSI+log(RemCortisolThreshold)+Age+DMRI+X3T | 456.51 | 427.5 | 429.3 | -203.8 | 407.5 |
| 48839 | PrevTSS+MaleProp+Undetect+Age+DMRI+X3T | 456.52 | 430.4 | 431.8 | -206.2 | 412.4 |
| 48844 | PrevTSS+MaleProp+Undetect+CSI+log(RemCortisolThreshold)+Age+GRE+DMRI+X3T | 456.53 | 421.9 | 424.6 | -199.0 | 397.9 |
| 49286 | PrevTSS+MaleProp+Undetect+RemCortisolThreshold+Age+DMRI+X3T | 456.81 | 427.8 | 429.6 | -203.9 | 407.8 |
| 49395 | PrevTSS+MaleProp+Undetect+log(RemCortisolThreshold)+Age+GRE+DMRI+X3T | 456.88 | 425.1 | 427.3 | -201.5 | 403.1 |
| 49812 | MaleProp+Undetect+log(RemCortisolThreshold)+Age+DMRI+X3T | 457.17 | 431.0 | 432.5 | -206.5 | 413.0 |
| 50053 | PrevTSS+MaleProp+Undetect+CSI+log(RemCortisolThreshold)+Age+DMRI+X3T | 457.38 | 425.6 | 427.8 | -201.8 | 403.6 |
| 50463 | PrevTSS+MaleProp+Undetect+log(RemCortisolThreshold)+Age+DMRI+X3T | 457.77 | 428.8 | 430.6 | -204.4 | 408.8 |

#### Supplementary Table 30. MRI Sequences without Undetect for Recurrence

|  | Model | BIC | AIC | AICc | ll | dev |
| --- | --- | --- | --- | --- | --- | --- |
| 1 | Macro+AvgFU+GRE | 431.44 | 413.9 | 414.5 | -200.9 | 401.9 |
| 2 | PrevTSS+Macro+AvgFU+GRE | 432.02 | 411.6 | 412.5 | -198.8 | 397.6 |
| 3 | Macro+RemCortisolThreshold+AvgFU+GRE | 432.63 | 412.2 | 413.1 | -199.1 | 398.2 |
| 4 | PrevTSS+Macro+RemCortisolThreshold+AvgFU+GRE | 433.31 | 410.0 | 411.1 | -197.0 | 394.0 |
| 5 | Macro+log(RemCortisolThreshold)+AvgFU+GRE | 433.43 | 413.0 | 413.9 | -199.5 | 399.0 |
| 6 | Macro+AvgFU+GRE+DMRI | 433.62 | 413.2 | 414.0 | -199.6 | 399.2 |
| 7 | Macro+CSI+AvgFU+GRE | 433.64 | 413.2 | 414.1 | -199.6 | 399.2 |
| 8 | CSI+AvgFU+GRE | 433.68 | 416.1 | 416.8 | -202.1 | 404.1 |
| … | … | … | … | … | … | … |
| … | … | … | … | … | … | … |
| … | … | … | … | … | … | … |
| 42799 | MaleProp+Macro+CSI+log(RemCortisolThreshold)+Age+DMRI+X3T | 453.86 | 424.9 | 426.7 | -202.4 | 404.9 |
| 42950 | PrevTSS+MaleProp+RemCortisolThreshold+Age+DMRI+X3T | 453.91 | 427.8 | 429.2 | -204.9 | 409.8 |
| 43241 | PrevTSS+MaleProp+log(RemCortisolThreshold)+Age+GRE+DMRI+X3T | 454.01 | 425.0 | 426.8 | -202.5 | 405.0 |
| 43300 | PrevTSS+MaleProp+Macro+CSI+RemCortisolThreshold+Age+DMRI+X3T | 454.03 | 422.2 | 424.4 | -200.1 | 400.2 |
| 43715 | MaleProp+log(RemCortisolThreshold)+Age+DMRI+X3T | 454.17 | 430.9 | 432.0 | -207.4 | 414.9 |
| 44880 | PrevTSS+MaleProp+CSI+log(RemCortisolThreshold)+Age+DMRI+X3T | 454.60 | 425.6 | 427.4 | -202.8 | 405.6 |
| 45577 | PrevTSS+MaleProp+log(RemCortisolThreshold)+Age+DMRI+X3T | 454.86 | 428.7 | 430.2 | -205.4 | 410.7 |
| 45860 | PrevTSS+MaleProp+Macro+CSI+log(RemCortisolThreshold)+Age+DMRI+X3T | 454.98 | 423.2 | 425.4 | -200.6 | 401.2 |

#### Supplementary Table 31. MRI Sequences without Macro or Undetect for Recurrence

|  | Model | BIC | AIC | AICc | ll | dev |
| --- | --- | --- | --- | --- | --- | --- |
| 8 | CSI+AvgFU+GRE | 433.68 | 416.1 | 416.8 | -202.1 | 404.1 |
| 14 | PrevTSS+CSI+AvgFU+GRE | 434.04 | 413.6 | 414.5 | -199.8 | 399.6 |
| 27 | CSI+RemCortisolThreshold+AvgFU+GRE | 434.68 | 414.2 | 415.1 | -200.1 | 400.2 |
| 31 | AvgFU+GRE | 434.83 | 420.2 | 420.6 | -205.1 | 410.2 |
| 32 | PrevTSS+AvgFU+GRE | 434.96 | 417.4 | 418.0 | -202.7 | 405.4 |
| 36 | PrevTSS+CSI+RemCortisolThreshold+AvgFU+GRE | 435.10 | 411.8 | 412.9 | -197.9 | 395.8 |
| 51 | CSI+AvgFU | 435.40 | 420.7 | 421.2 | -205.4 | 410.7 |
| 56 | CSI+AvgFU+GRE+DMRI | 435.46 | 415.0 | 415.9 | -200.5 | 401.0 |
| … | … | … | … | … | … | … |
| … | … | … | … | … | … | … |
| … | … | … | … | … | … | … |
| 42066 | PrevTSS+MaleProp+Age+DMRI+X3T | 453.60 | 430.3 | 431.4 | -207.1 | 414.3 |
| 42317 | PrevTSS+MaleProp+CSI+log(RemCortisolThreshold)+Age+GRE+DMRI+X3T | 453.69 | 421.9 | 424.1 | -199.9 | 399.9 |
| 42501 | MaleProp+CSI+log(RemCortisolThreshold)+Age+DMRI+X3T | 453.75 | 427.6 | 429.0 | -204.8 | 409.6 |
| 42950 | PrevTSS+MaleProp+RemCortisolThreshold+Age+DMRI+X3T | 453.91 | 427.8 | 429.2 | -204.9 | 409.8 |
| 43241 | PrevTSS+MaleProp+log(RemCortisolThreshold)+Age+GRE+DMRI+X3T | 454.01 | 425.0 | 426.8 | -202.5 | 405.0 |
| 43715 | MaleProp+log(RemCortisolThreshold)+Age+DMRI+X3T | 454.17 | 430.9 | 432.0 | -207.4 | 414.9 |
| 44880 | PrevTSS+MaleProp+CSI+log(RemCortisolThreshold)+Age+DMRI+X3T | 454.60 | 425.6 | 427.4 | -202.8 | 405.6 |
| 45577 | PrevTSS+MaleProp+log(RemCortisolThreshold)+Age+DMRI+X3T | 454.86 | 428.7 | 430.2 | -205.4 | 410.7 |

#### Supplementary Table 32. Year without Macro for Recurrence

|  | Model | BIC | AIC | AICc | ll | dev |
| --- | --- | --- | --- | --- | --- | --- |
| 51 | CSI+AvgFU | 435.40 | 420.7 | 421.2 | -205.4 | 410.7 |
| 92 | PrevTSS+CSI+AvgFU | 436.09 | 418.5 | 419.2 | -203.3 | 406.5 |
| 140 | CSI+AvgFU+Year | 436.64 | 419.1 | 419.7 | -203.5 | 407.1 |
| 147 | AvgFU | 436.69 | 424.9 | 425.2 | -208.5 | 416.9 |
| 152 | CSI+RemCortisolThreshold+AvgFU | 436.70 | 419.1 | 419.8 | -203.6 | 407.1 |
| 208 | PrevTSS+CSI+AvgFU+Year | 436.99 | 416.5 | 417.4 | -201.3 | 402.5 |
| 214 | PrevTSS+AvgFU | 437.11 | 422.4 | 422.9 | -206.2 | 412.4 |
| 272 | CSI+log(RemCortisolThreshold)+AvgFU | 437.43 | 419.9 | 420.5 | -203.9 | 407.9 |
| … | … | … | … | … | … | … |
| … | … | … | … | … | … | … |
| … | … | … | … | … | … | … |
| 39044 | MaleProp+Undetect+RemCortisolThreshold+Age+Year | 452.67 | 429.4 | 430.5 | -206.7 | 413.4 |
| 39233 | PrevTSS+MaleProp+Undetect+CSI+RemCortisolThreshold+Age+Year | 452.73 | 423.7 | 425.5 | -201.9 | 403.7 |
| 39975 | PrevTSS+MaleProp+Undetect+CSI+log(RemCortisolThreshold)+Age | 452.96 | 426.8 | 428.3 | -204.4 | 408.8 |
| 40584 | PrevTSS+MaleProp+Undetect+RemCortisolThreshold+Age+Year | 453.12 | 427.0 | 428.4 | -204.5 | 409.0 |
| 41204 | MaleProp+Undetect+log(RemCortisolThreshold)+Age+Year | 453.32 | 430.0 | 431.1 | -207.0 | 414.0 |
| 41250 | PrevTSS+MaleProp+Undetect+log(RemCortisolThreshold)+Age | 453.34 | 430.0 | 431.2 | -207.0 | 414.0 |
| 41359 | PrevTSS+MaleProp+Undetect+CSI+log(RemCortisolThreshold)+Age+Year | 453.37 | 424.4 | 426.2 | -202.2 | 404.4 |
| 42432 | PrevTSS+MaleProp+Undetect+log(RemCortisolThreshold)+Age+Year | 453.73 | 427.6 | 429.0 | -204.8 | 409.6 |

#### Supplementary Table 33. Year without Undetect for Recurrence for Recurrence

|  | Model | BIC | AIC | AICc | ll | dev |
| --- | --- | --- | --- | --- | --- | --- |
| 9 | Macro+AvgFU | 433.83 | 419.2 | 419.6 | -204.6 | 409.2 |
| 23 | PrevTSS+Macro+AvgFU | 434.65 | 417.1 | 417.7 | -202.5 | 405.1 |
| 42 | Macro+RemCortisolThreshold+AvgFU | 435.31 | 417.7 | 418.4 | -202.9 | 405.7 |
| 51 | CSI+AvgFU | 435.40 | 420.7 | 421.2 | -205.4 | 410.7 |
| 59 | Macro+AvgFU+Year | 435.56 | 418.0 | 418.6 | -203.0 | 406.0 |
| 66 | Macro+log(RemCortisolThreshold)+AvgFU | 435.88 | 418.3 | 419.0 | -203.2 | 406.3 |
| 71 | Macro+CSI+AvgFU | 435.92 | 418.4 | 419.0 | -203.2 | 406.4 |
| 80 | PrevTSS+Macro+AvgFU+Year | 435.99 | 415.5 | 416.4 | -200.8 | 401.5 |
| … | … | … | … | … | … | … |
| … | … | … | … | … | … | … |
| … | … | … | … | … | … | … |
| 30240 | MaleProp+log(RemCortisolThreshold)+Age+Year | 450.30 | 429.9 | 430.7 | -207.9 | 415.9 |
| 30529 | PrevTSS+MaleProp+log(RemCortisolThreshold)+Age | 450.38 | 429.9 | 430.8 | -208.0 | 415.9 |
| 31558 | PrevTSS+MaleProp+Macro+CSI+log(RemCortisolThreshold)+Age | 450.63 | 424.5 | 425.9 | -203.2 | 406.5 |
| 31694 | MaleProp+Macro+CSI+log(RemCortisolThreshold)+Age+Year | 450.67 | 424.5 | 426.0 | -203.3 | 406.5 |
| 31835 | PrevTSS+MaleProp+log(RemCortisolThreshold)+Age+Year | 450.71 | 427.4 | 428.5 | -205.7 | 411.4 |
| 32146 | PrevTSS+MaleProp+CSI+log(RemCortisolThreshold)+Age+Year | 450.79 | 424.6 | 426.1 | -203.3 | 406.6 |
| 32545 | PrevTSS+MaleProp+Macro+CSI+RemCortisolThreshold+Age+Year | 450.89 | 421.9 | 423.7 | -201.0 | 401.9 |
| 35007 | PrevTSS+MaleProp+Macro+CSI+log(RemCortisolThreshold)+Age+Year | 451.54 | 422.6 | 424.4 | -201.3 | 402.6 |

#### Supplementary Table 34. Year without Macro or Undetect for Recurrence

|  | Model | BIC | AIC | AICc | ll | dev |
| --- | --- | --- | --- | --- | --- | --- |
| 51 | CSI+AvgFU | 435.40 | 420.7 | 421.2 | -205.4 | 410.7 |
| 92 | PrevTSS+CSI+AvgFU | 436.09 | 418.5 | 419.2 | -203.3 | 406.5 |
| 140 | CSI+AvgFU+Year | 436.64 | 419.1 | 419.7 | -203.5 | 407.1 |
| 147 | AvgFU | 436.69 | 424.9 | 425.2 | -208.5 | 416.9 |
| 152 | CSI+RemCortisolThreshold+AvgFU | 436.70 | 419.1 | 419.8 | -203.6 | 407.1 |
| 208 | PrevTSS+CSI+AvgFU+Year | 436.99 | 416.5 | 417.4 | -201.3 | 402.5 |
| 214 | PrevTSS+AvgFU | 437.11 | 422.4 | 422.9 | -206.2 | 412.4 |
| 272 | CSI+log(RemCortisolThreshold)+AvgFU | 437.43 | 419.9 | 420.5 | -203.9 | 407.9 |
| … | … | … | … | … | … | … |
| … | … | … | … | … | … | … |
| … | … | … | … | … | … | … |
| 29400 | PrevTSS+MaleProp+RemCortisolThreshold+Age+Year | 450.09 | 426.8 | 427.9 | -205.4 | 410.8 |
| 29470 | MaleProp+CSI+log(RemCortisolThreshold)+Age+Year | 450.11 | 426.8 | 427.9 | -205.4 | 410.8 |
| 29519 | PrevTSS+MaleProp+CSI+RemCortisolThreshold+Age+Year | 450.12 | 424.0 | 425.4 | -203.0 | 406.0 |
| 29570 | PrevTSS+MaleProp+CSI+log(RemCortisolThreshold)+Age | 450.15 | 426.8 | 428.0 | -205.4 | 410.8 |
| 30240 | MaleProp+log(RemCortisolThreshold)+Age+Year | 450.30 | 429.9 | 430.7 | -207.9 | 415.9 |
| 30529 | PrevTSS+MaleProp+log(RemCortisolThreshold)+Age | 450.38 | 429.9 | 430.8 | -208.0 | 415.9 |
| 31835 | PrevTSS+MaleProp+log(RemCortisolThreshold)+Age+Year | 450.71 | 427.4 | 428.5 | -205.7 | 411.4 |
| 32146 | PrevTSS+MaleProp+CSI+log(RemCortisolThreshold)+Age+Year | 450.79 | 424.6 | 426.1 | -203.3 | 406.6 |
