# Supplementary figures and images for: The clinical outcomes of imaging modalities for surgical management Cushing’s disease – A systematic review and meta-analysis
Source: Front Endocrinol (Lausanne). 2023 Jan 13;13:1090144. doi: 10.3389/fendo.2022.1090144 (PMC9880448; doi:10.3389/fendo.2022.1090144)

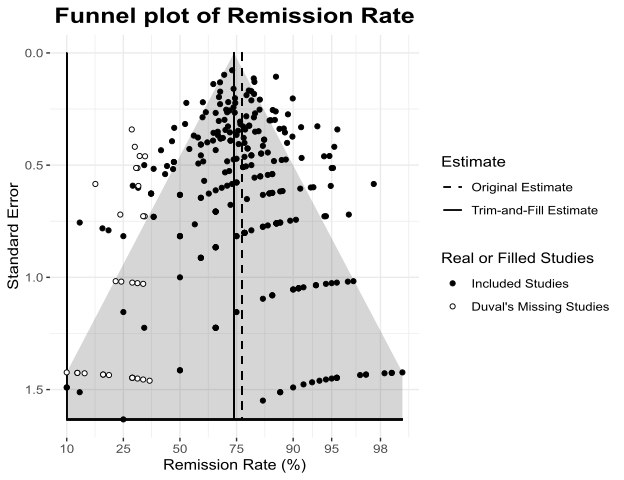

Supplement: Supplementary Figure 1 — Funnel plot of univariable remission with Duval’s trim-and-fill. [file Image_1.tiff]

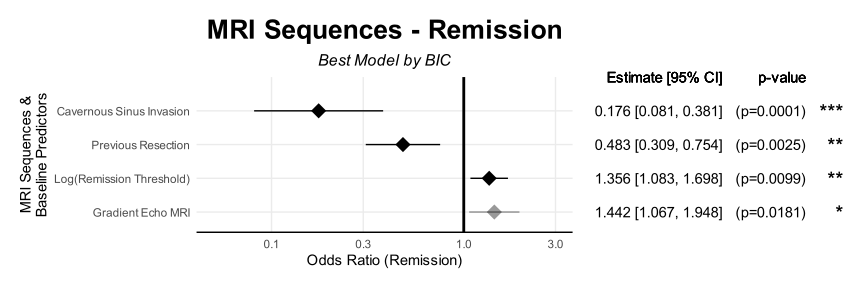

Supplement: Supplementary Figure 2 — Effect of removing macroadenoma from the MRI sequences model. Note that GRE is now a stronger predictor of remission. [file Image_2.tiff]

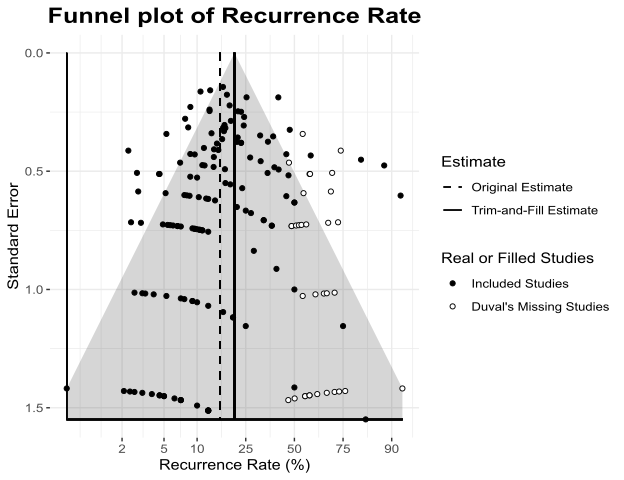

Supplement: Supplementary Figure 3 — Funnel plot of univariable recurrence with Duval’s trim-and-fill. [file Image_3.tiff]

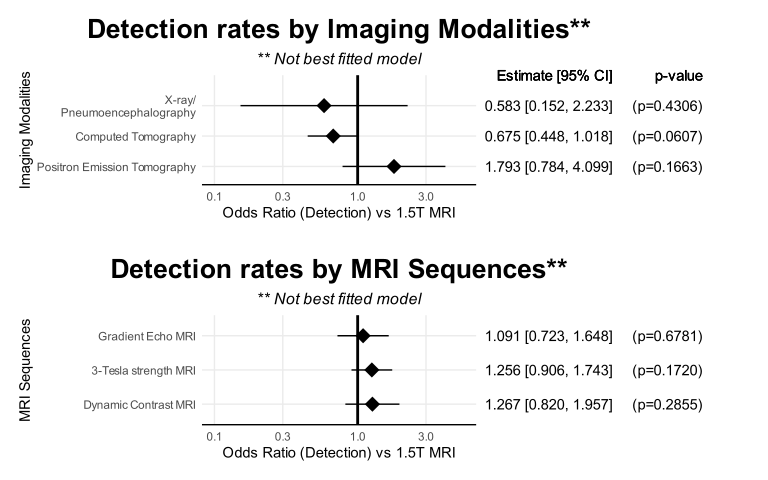

Supplement: Supplementary Figure 4 — Detection rates by imaging modalities and MRI sequences.Detection rates of (A) Imaging modalities and (B) MRI sequences. Models incorporating all imaging modalities and MRI sequences shown regardless of performance (BIC) as none were significantly associated with changes in detection. The point denotes the estimated effect size, and the line denotes the 95% confidence interval of that estimate. [file Image_4.tiff]
